# One-Pot Metal-Free Synthesis of Diarylamines from Aromatic Aldehydes and Anilines

**DOI:** 10.1021/acs.joc.5c01253

**Published:** 2025-07-23

**Authors:** Piotr Szcześniak, Bartłomiej Furman

**Affiliations:** 154690 Institute of Organic Chemistry, Polish Academy of Sciences, Kasprzaka 44/52, 01-224 Warsaw, Poland

## Abstract

A novel and environmentally friendly method has been developed for the synthesis of a broad range of diarylamines from inexpensive and readily available starting materials, such as aromatic aldehydes and amines. The process follows a one-pot strategy in which imine formation is succeeded by an oxidative rearrangement analogous to the Meinwald reaction and concludes with a light-induced deformylation step. This efficient transformation proceeds in a single vessel with a high atom economy under mild, metal-free conditions. The methodology was further demonstrated as a key step in the synthesis of phentolamine, a reversible, nonselective α-adrenergic blocker used in the treatment of hypertensive emergencies.

## Graphical abstract

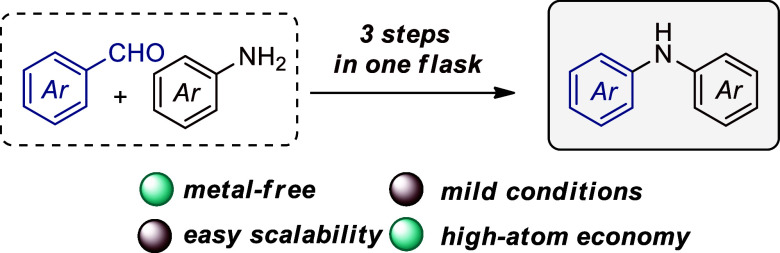

## Introduction

Diarylamines represent a significant class of organic compounds widely employed in pharmaceuticals, agrochemicals, dyes, and electroluminescent materials and as ligands in transition metal catalysis.^1^ The predominant method for synthesis of diarylamines involves transition-metal-catalyzed coupling reactions between anilines and functionalized arenes, such as boronic acids or aryl halides, promoted by either copper (Cu) or palladium (Pd) catalysts (Scheme 1a). Notable examples include the Ullmann–Goldberg^2^ and Chan–Evans–Lam^3^ reactions under Cu-catalysis, as well as the Buchwald–Hartwig^4^ reaction under Pd-catalysis. Despite their versatility, these methods require transmetalation with metal catalysts sensitive to air and moisture. The toxicity concerns associated with heavy metals and the challenges related to the removal of residual metal contaminants from products remain important considerations, particularly in pharmaceutical applications, where even trace amounts of heavy metals are often deemed undesirable. Consequently, recent research has focused on developing transition-metal-free synthetic strategies that employ inexpensive and readily available starting materials (Scheme 1b). For instance, the Csákÿ group reported the direct synthesis of diphenylamines from nitrosoarenes and boronic acids.^5^ Visible-light- and triphenylphosphine-mediated intermolecular reductive amination between nitroarenes and boronic acids, developed by Baitalik and Jana, serves as a mild-condition method for the preparation of diarylamines.^6^ The Larock group presented the facile, transition-metal-free *N*-arylation of amines by the reaction of silylaryltriflates with a variety of amines promoted by CsF.^7^ The metal-free synthesis of diarylamines via the direct amination of phenols using aminating reagents was reported by Wu and co-workers.^8^ The synthesis of symmetrical diarylamines via PPA-mediated amination of arenes with 2-nitropropane was developed by Aksenov and Rubin.^9^ In turn, a protocol for the *one-pot* synthesis of diarylamines via the Smiles rearrangement under microwave irradiation was developed by Zuo and co-workers.^10^ Iodine-catalyzed cross-dehydrogenative aromatization of cyclohexenones and anilines using DMSO and O_2_ as oxidants to synthesize diarylamines was reported by the Pan group.^11^ Very recently, the Greaney group presented diarylamine synthesis via the desulfinylative Smiles rearrangement.^12^

**Scheme 1:**
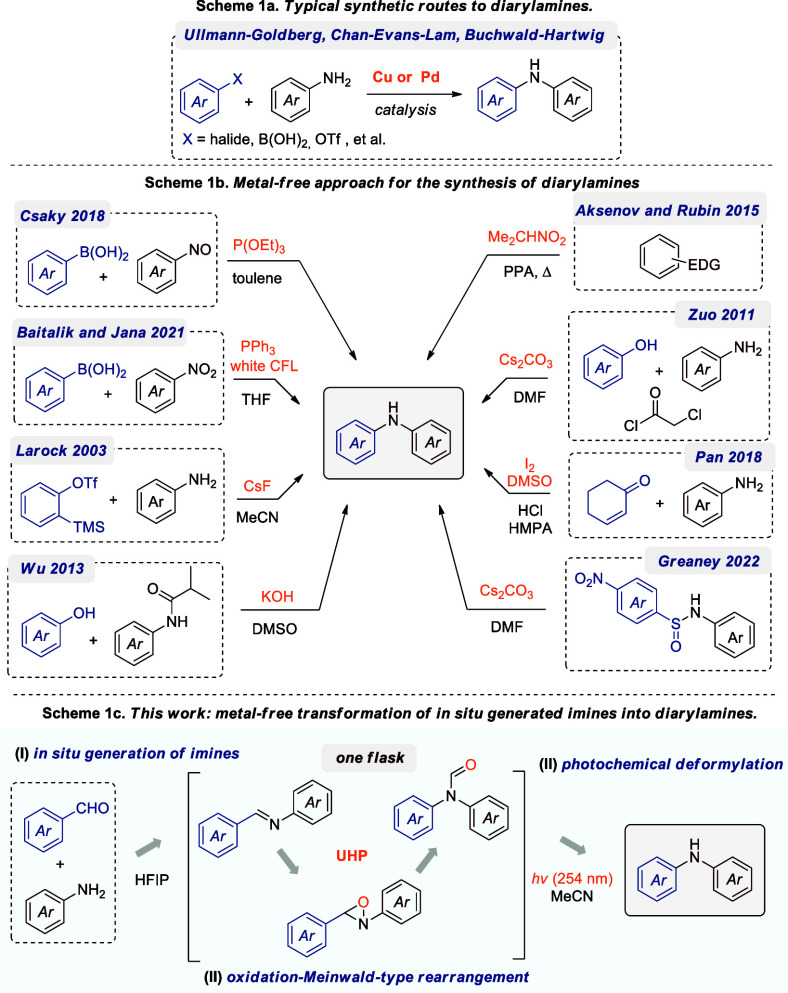
Background and Study Synopsis

The main limitation of the presented strategies is the limited availability of starting materials. Given the importance of diarylamines, we propose here a comprehensive green protocol for the synthesis of diarylamines from simple, inexpensive, and readily available starting materials such as aromatic aldehydes and amines. The strategies outlined in Scheme 1c involve three steps: imine formation, oxidation/Meinwald rearrangement, and photochemical deformylation reaction. This sequential process is carried out in a single flask, eliminating the need for the difficult isolation of intermediates. The proposed methodology is based on the protocol for the direct synthesis of *N,N*-disubstituted formamides via the oxidation of imines using an HFIP/UHP system,^13^ in conjunction with our interest in Fries-type photochemical rearrangement.^14^ The combination of these two concepts (the simple transformation of imines to formamides and subsequent light-promoted homolytic cleavage of the amide bond) provides an efficient and environmentally sustainable approach to accessing a wide range of symmetrical and asymmetrical diarylamines.

## Results and Discussion

The research commenced by optimizing the conditions for the transformation of in situ-generated aldimine from aniline and benzaldehyde into *N*,*N*-diphenylformamide. The main emphasis was placed on the identification of the optimal oxidizing agent: either H_2_O_2_ (30% v/v) or UHP (urea-hydrogen peroxide), (Table 1, entry 1 vs 2) and fluorinated solvent: HFIP (1,1,1,3,3,3-hexafluoroispropanol) or TFE (2,2,2-trifluoroethanol) (Table 1, entry 1 vs 3). Subsequently, the optimization of conditions for the light-induced deformylation of *N*,*N*-diphenylformamide was investigated, including the selection of the optimal source of light, solvent, and concentration (Table 1, entries 4–12).

**Table 1:**
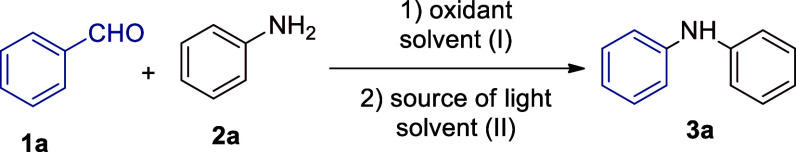
Optimization of the Process Conditions^a^

entry	oxidant	solvent I	light	solvent II	yield (%)^b^
1	UHP	HFIP	UV-C	MeOH	38
2	H_2_O_2_ (30%)	HFIP	UV-C	MeOH	25
3	UHP	TFE			
4	UHP	HFIP	UV-C	*i*-PrOH	34
5	UHP	HFIP	UV-C	EtOH	38
6	UHP	HFIP	UV-C	THF	38
7	UHP	HFIP	UV-C	MeCN	43
8	UHP	HFIP	UV-C	MeCN	45^c^
9	UHP	HFIP	UV-C	MeCN	35^d^
10	UHP	HFIP	UV-C	MeCN	38^e^
11	UHP	HFIP	UV-B	MeCN	0
12	UHP	HFIP	UV-A	MeCN	0

a Reaction conditions: benzaldehyde (0.25 mmol), aniline (0.25 mmol), HFIP (0.25 mL), 60 °C, 2.5 h; then, the oxidant (0.5 mmol) was added, and the reaction was left overnight at 45 °C; next, after removing HFIP, degassed solvent (II) (4 mL, *C* = 0.06) was added under argon, and the reaction was irradiated in quartz glass in a self-made photoreactor with eight UV-C lamps (9W) (see the SI).

b Determined by GC.

c 2 mL of MeCN was used (*C* = 0.03).

d 8 mL of MeCN was used (*C* = 0.12).

e Nondegassed MeCN was used.

The motivation for the study was to identify compatible conditions for both reactions, making the process operationally simple single-flask protocol without the need for isolating intermediate products. Following an extensive screening of the reaction conditions, we identified the optimal procedure for the preparation of diphenylamine **3a** (Scheme 2), which involves a combination of three steps. The first step, *generation of imine*, requires heating at 60 °C for 2.5 h an equimolar solution of benzaldehyde **1a** and aniline **2a** in HFIP (1 mmol/mL) in a quartz vial. The second step, *oxaziridine formation and subsequent Meinwald-type rearrangement*, needs the addition of UHP (2 equiv) and stirring overnight at 45 °C. The last and third step, *photochemical deformylation*, demands HFIP evaporation, addition of degassed MeCN (0.03 mmol/mL), and then UV-C lamp irradiation (8x 9W, λ_max_ 254 nm) for 2 h. The evaporation of the solvent and purification by flash column chromatography allow us to obtain pure diphenylamine **3a** (43% yield for model substrates, 75% per step).

**Scheme 2:**
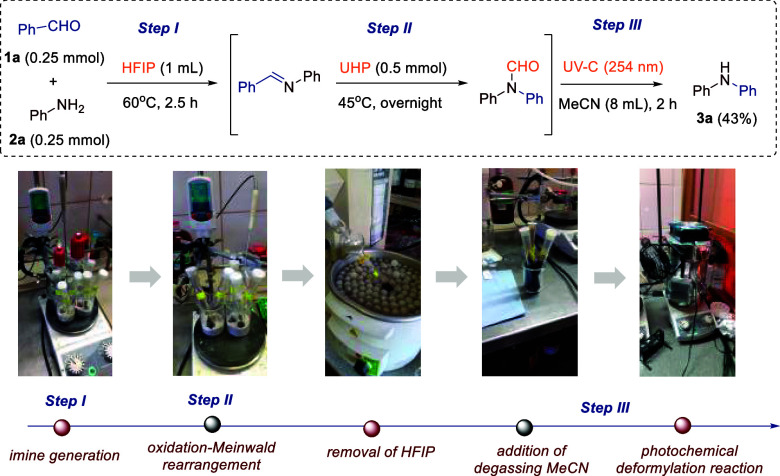
Developed Process

We next began to explore the generality of the protocol by testing various substituted aldehydes **1b–** **s** as coupling partners with anilines (Table 2). In general, the system tolerates aliphatic substituted rings at all positions **3c**–**e**, **3k**, **3m**, and **3n**. Similar success was achieved with halogenated rings **3g** and **3h**, which can be challenging to synthesize using transition metal catalysis. Additionally, the scope encompassed substrates featuring both electron-rich rings **3b**, **3l**, **3o**, and **3s** and relatively electron-poor ones **3i** and **3j**, although substrates with electron-deficient rings led to desired products with a low yield. Furthermore, the reaction was found to be effective for extended aryl systems, such as the *N*-naphthyl example **3p** and **3r**, and the heterocyclic compound **3s**. In further studies, we explored the reactivity of variously substituted anilines as coupling partners with benzaldehyde **1a**. However, regardless of the type and position of the substituent in the ring, the protocol was unsuccessful (see the SI). Except for examples **3x**–**z**, in most cases, we observed decomposition and hydrolysis products of aldimines. These results are in line with literature precedents.^13,15^ Using the developed method, several known important synthetic intermediates were prepared **3b, 3t–x** that have been employed for the synthesis of the following: *c-*myc inhibitor, which is useful for the treatment of colorectal cancer;^16^ natural alkaloid *Murrafoline A,* which shows antimicrobial and cytotoxic activities;^17^ *Ralinepag,* a selective prostacyclin receptor agonist developed for the treatment of pulmonary arterial hypertension;^18^ *Isogirinimbine,* a natural alkaloid exhibiting antioxidant and antimicrobial activities;^19^ and *flufenamic acid,* a nonsteroidal anti-inflammatory drug, used to reduce inflammation and pain.^20^

**Table 2:**
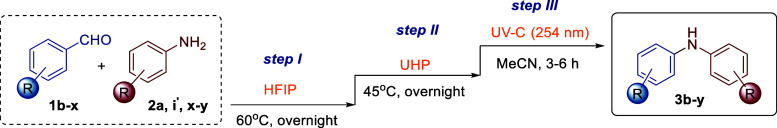

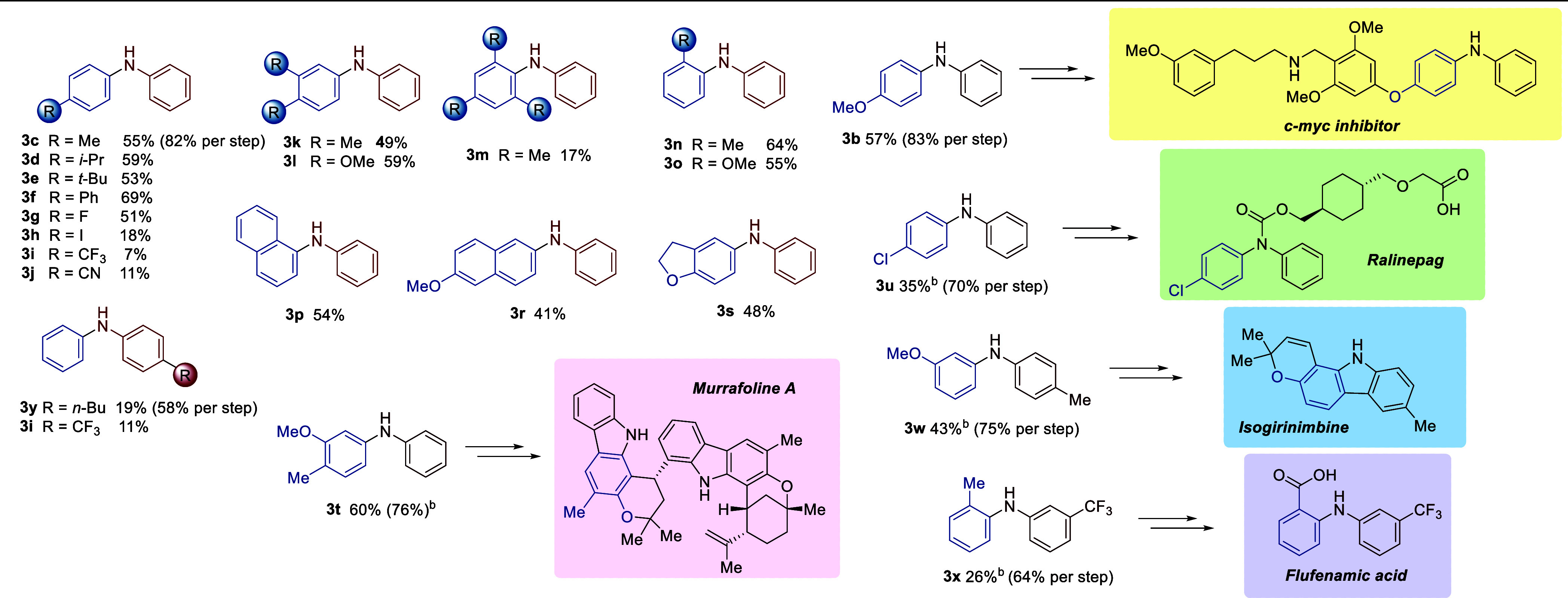
Study of the Reaction Scope^a^

a Reaction conditions: aldehyde (1.0 mmol), amine (1.0 mmol), HFIP (1.0 mL), 60 °C, overnight; next, UHP (2.0 mmol), 45 °C, overnight; next, degassed MeCN (8 mL, *C* = 0.03) irradiation in a quartz glass with eight UV-C lamps (9W).

b Deformylation reaction was performed in an aqueous NaOH solution instead of irradiation.

The developed protocol uses inexpensive and readily available reagents, including p-anisidine (190 EUR per liter, Sigma-Aldrich), aniline (65 EUR per liter, Sigma-Aldrich), and UHP (137 EUR per 500 g, Sigma-Aldrich). The process is highly scalable, maintaining efficiency with a 10-fold increase in the amount of the starting material (Scheme 3). To achieve this scalability, the photoinduced deformylation reaction was carried out in a continuous flow after filtration of solid UHP residues from the diethyl ether solution. In addition, it was demonstrated that the solvents (HFIP and MeCN) can be almost completely recovered by simple distillation and reused for subsequent reaction cycles, which is a significant advantage for potential industrial applications.

**Scheme 3:**
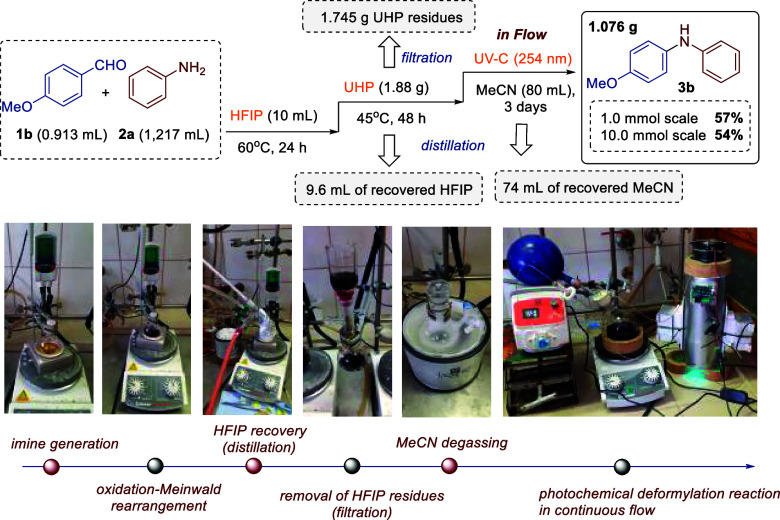
Reaction on a 10.0 mmol Scale

To further illustrate the utility of the developed methodology, we used it as a key step in the total synthesis of *Phentolamine* (Scheme 4). *Phentolamine*, the active ingredient in *Regitine*, is a reversible, nonselective α-adrenergic receptor antagonist used in the treatment of hypertensive emergencies and certain cases of pheochromocytoma. It is also used in the treatment of dermal necrosis caused by the extravasation of vasoconstrictors such as norepinephrine and in dental procedures to accelerate the reversal of local anesthesia.^21^

**Scheme 4:**
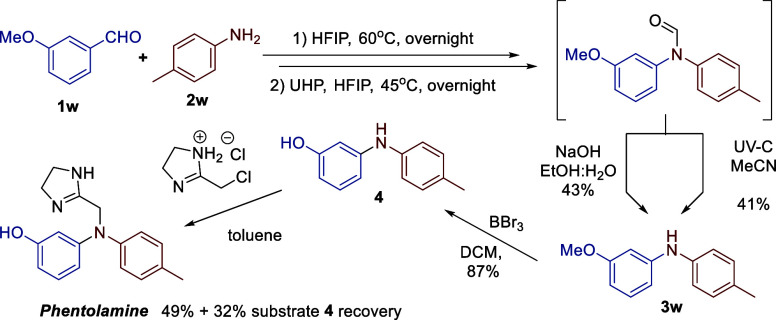
Total Synthesis of Phentolamine

The suitable precursor **3w** for the synthesis of *Phentolamine* was obtained from 3-methoxybenzaldehyde **1w** and p-toluidine **2w**. Two complementary deformylation strategies were demonstrated: photochemical and hydrolysis in the presence of an aqueous NaOH solution. The synthesized diarylamine **3w** was subjected to demethylation followed by *N*-alkylation with commercially available 2-(chloromethyl)-1H-imidazole hydrochloride according to the literature procedure.^5^ This process afforded *Phentolamine* with an efficiency of 49% (Scheme 4).

Based on experimental evidence and literature precedent,^23^ the proposed reaction mechanism (Scheme 5) begins with HFIP-assisted oxygen transfer from H_2_O_2_ ^22^ to imine **A**, resulting in the formation of oxaziridine **B**. This is followed by a Meinwald-type rearrangement,^13^ mediated by HFIP, which converts oxaziridine into amide **C**. In the final step, irradiation of the amide bond with 254 nm light induces homolytic cleavage, generating a nitrogen-centered radical.^23^ This radical is subsequently trapped by the solvent, yielding the desired amine **D**.

**Scheme 5:**
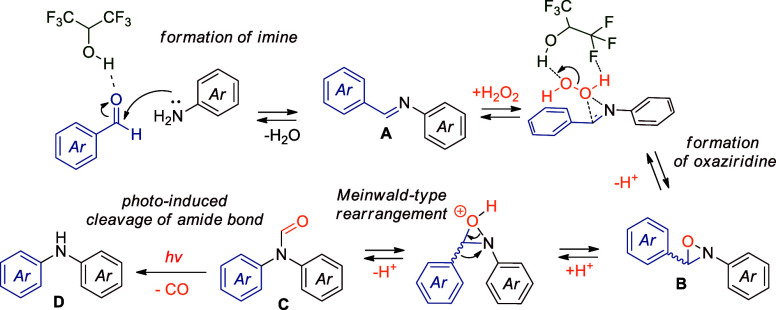
Proposed Reaction Mechanism

## Conclusions

In conclusion, we have developed a novel, metal-free approach for the preparation of diarylamines using simple, inexpensive, and nontoxic reagents: urea-hydrogen peroxide, aromatic aldehydes, and anilines. By avoiding the use of transition metals, this method eliminates concerns related to toxicity, environmental impact, and residual metal contamination in the final products. The combination of cost-efficiency, universal availability of starting materials, and an effective solvent recovery procedure positions this strategy as a highly sustainable and practical alternative to existing methods. Furthermore, the simple reaction conditions and scalability of the process highlight the significant potential for industrial applications. The effectiveness of the developed strategy has been demonstrated through the synthesis of several important precursors used in the total synthesis of active pharmaceutical ingredients and natural products, as well as in the total synthesis of *Phentolamine*, the active ingredient in *Regitine*.

## Supplementary Material



## Data Availability

The data underlying this study are available in the published article and its Supporting Information.

## Notes

General information, optimization studies, safety and hazard considerations, detailed synthetic procedures including scale-up, characterization data, reaction scope investigations including unsuccessful results, literature references, and copies of ^1^H, ^13^C, and ^19^F NMR spectra (PDF)
