# Supplementary material for: One-Pot Metal-Free Synthesis of Diarylamines from Aromatic Aldehydes and Anilines
Source: J Org Chem. 2025 Jul 23;90(36):12633–7. doi: 10.1021/acs.joc.5c01253 (PMC12442086; doi:10.1021/acs.joc.5c01253)

One-Pot Metal-Free Synthesis of Diarylamines from Aromatic Aldehydes and Anilines.

Piotr Szcześniak* and Bartłomiej Furman

Institute of Organic Chemistry, Polish Academy of Sciences

Kasprzaka 44/52, 01-224 Warsaw, Poland

Corresponding authors: piotr.szczesniak@icho.edu.pl

Table of Context:

1. General Information. *S2*

2. Optimization studied leading to diphenylamine **3a**. *S2*

3. Safety and hazard considerations. *S2*

4. General procedure for the synthesis of diarylamines **3b-y** *via* tandem one-pot imine formation followed by an oxidative Meinwald-type rearrangement sequence, and deformylation reaction. *S2-S4*

5. Synthesis of 4-Methoxy-*N*-phenylaniline **3b** in 10 mmol scale. *S4-S6*

6. Characterization Data. *S7-S13*

7. Synthesis of Phentolamine. *S13-S14*

8. Studies on the reaction scope: the reactivity of variously substituted anilines as coupling partners with benzaldehyde, unsuccessful results. *S14*

9. Literature. *S15*

10. Copies of ^{1}H, ^{13}C, ^{19}F NMR spectra. *S16-S42*

1. General information:

^{1}H NMR and ^{13}C NMR spectra were recorded on a Bruker 400 and Varian VNMRS 600 spectrometers. ^{1}H NMR spectra were referenced to: chloroform-*d* (δ = 7.26 ppm), ^{13}C NMR spectra were referenced to: chloroform-*d* (δ = 77.16 ppm). Chemical shifts (δ) were given in ppm and coupling constants (*J*) were given in Hertz (Hz). Multiplicity was indicated as follows: s (singlet), bs (brought singlet), d (doublet), t (triplet), q (quartet), m (multiplet), dd (doublet of doublet). Thin layer chromatography was performed on Merck aluminium sheet Silica Gel 60 F254. Flash column chromatography was carried out using Merck silica gel (230-400 mesh).

2. Safety and hazard considerations:

Urea hydrogen peroxide (UHP, CAS 124-43-6) is a mild solid oxidant but should be handled with care, as it may cause skin and eye irritation and can support combustion. Hexafluoroisopropanol (HFIP, CAS 920-66-1) is a volatile, flammable solvent that may cause irritation to the skin, eyes, and respiratory system. All experiments should be conducted in a well-ventilated fume hood using appropriate personal protective equipment (PPE). Standard laboratory safety procedures for handling oxidants and volatile organic solvents should be strictly followed.

3. Optimization studied leading to diphenylamine 3a.

Yield of model reaction were determined by GC analyses (Table 1) performed on Clarus PerkinElmer 680 gas chromatograph equipped with a split-mode capillary injection system and flame ionization detector using capillary column ZB-5HT Inferno (30 m × 0.25 mm x 0.25 µm, Phenomenex). Chromatography conditions: carrier gas – helium (1mL/min.), 100°C/9 min. – 20°/min. – 280°C/5 min.; injector temperature: 270°C (*const*); detector temperature 200°C. Retention time of the respective compounds are as follow: benzaldehyde **1a** 2.06 min.; aniline **2a** 2.08 min.; *N*-Benzylideneaniline – 6.92 min.; *N*,*N*-diphenylformamide 9.25 min.; diphenylamine **3a** 5.93 min.

4. General procedure for the synthesis of diarylamines 3b-s *via* tandem *one-pot* imine formation followed by an oxidative Meinwald-type rearrangement sequence, and deformylation reaction.

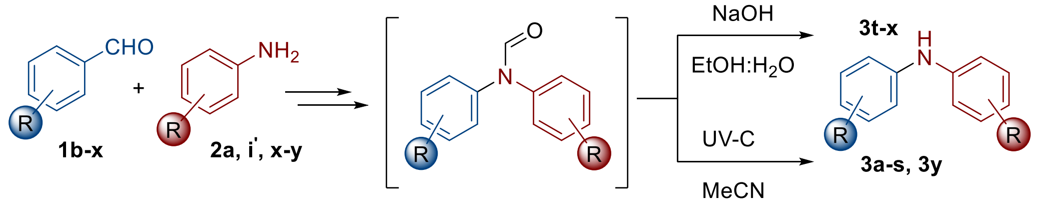

General remarks for photochemical deformylation reaction:

The photochemical deformylation reaction was performed in a Rayonet-type, self-made photoreactor consisting of eight UV-C lamps (Osram, Puritec, HNS, S 9W, λmax 254 nm). (The distance between the reaction vessel and the light source was 1.5 cm) For the detailed plan for the construction of the photoreactor, see ref.[1]

Rayonet-type, self-made photoreactor consisting of the following elements:
cooling system ,temperature control, mixing system test tube racks, quartz vial, 8 x UV-C lamps (*Osram, Puritec, HNS, S 9W*)

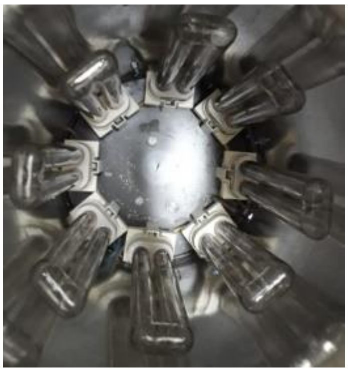

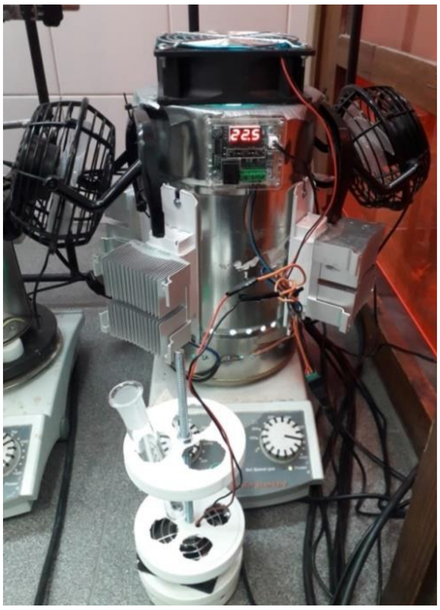

General procedure:

Generation of imine: To a quartz vial, aromatic aldehyde (1.0 mmol), aniline (1.0 mmol), and HFIP (1,1,1,3,3,3-hexafluoroisopropanol) (1.0 mL) were added, and the reaction mixture was stirred overnight at 60°C using heating mantle (Picture S1).

Oxidative Meinwald-type rearrangement sequence: After cooling to room temperature, UHP (urea-hydrogen peroxide) (2.0 equiv., 2.0 mmol, 188 mg) was added, and the reaction was left overnight at 45°C using heating mantle (Picture S2).

Deformylation reaction:

via irradiation: HFIP was removed by rotary evaporation under reduced pressure (Picture 3). The quartz vial was then placed in a glove box, and degassed MeCN (8 mL) was added. The vial was sealed with a septum wrapped in aluminum foil and transferred to a photoreactor (Picture 4). The reaction mixture was irradiated with eight UV-C lamps (9W, 254 nm) at an internal temperature of 25-35°C for 3-6 hours (The distance between the reaction vessel and the light source was 1.5 cm)(Picture S5). After evaporation of the solvent, the residue was preabsorbed onto silica gel and purified by flash chromatography.

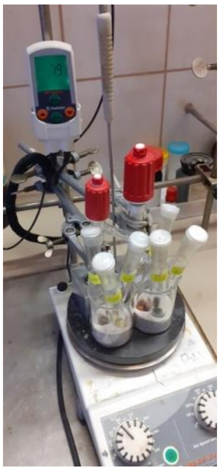

Picture S1.

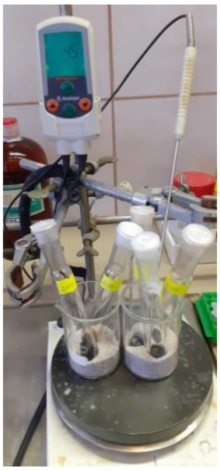

Picture S2.

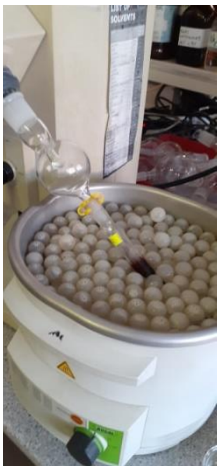

Picture S3.

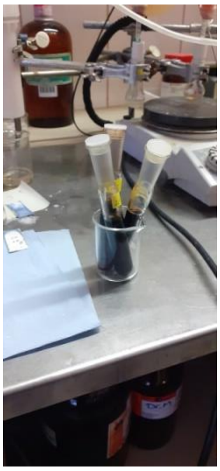

Picture S4.

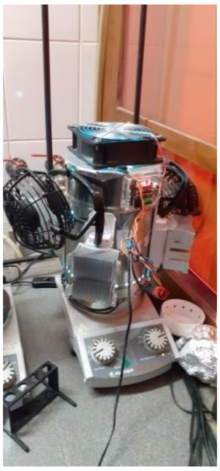

Picture S5.

via hydrolysis: After removal of HFIP by rotary evaporation under reduced pressure, the residue was dissolved in a mixture of EtOH (10.0 mL) and H_2O (1.0 mL). NaOH (8.0 equiv., 8 mmol, 320 mg) was then added, and the reaction mixture was refluxed for 3 h. After cooling to room temperature, EtOH was removed by rotary evaporation under reduced pressure. The residue was diluted with saturated aqueous NH_4Cl solution (10 mL) and AcOEt (10 mL). After phase separation, the aqueous layer was extracted with AcOEt (3×5 mL). The combined organic layers were dried over anhydrous Na_2SO_4. After solvent evaporation, the residue was pre-adsorbed onto silica gel and purified by flash chromatography.

5. Synthesis of 4-Methoxy-*N*-phenylaniline 3b in 10 mmol scale.

General remarks for photochemical deformylation reaction in 10 mmol scale performed in continues flow:

Photochemical deformylation reaction in continues flow was performed in a closed system under an argon atmosphere in self-made flow set consisting of: Rayonet-type, self-made photoreactor (*with four UV-C lamps Osram, Puritec, HNS, S 9W,* λmax 254 nm), UV-transparent FEP tubing of dimensions 0.7 mm i.d. × 1.1 mm o.d, wound on a quartz tube of dimensions 18 cm in length and 4 cm in diameter (*loop length 14 m, capacity 7 mL, number of coils 111*), peristaltic pump (*model LLG-uniPERIPUMP 1*), magnetic stirrer. The distance between the UV-transparent FEP tubing and the light source was 1.0 cm).

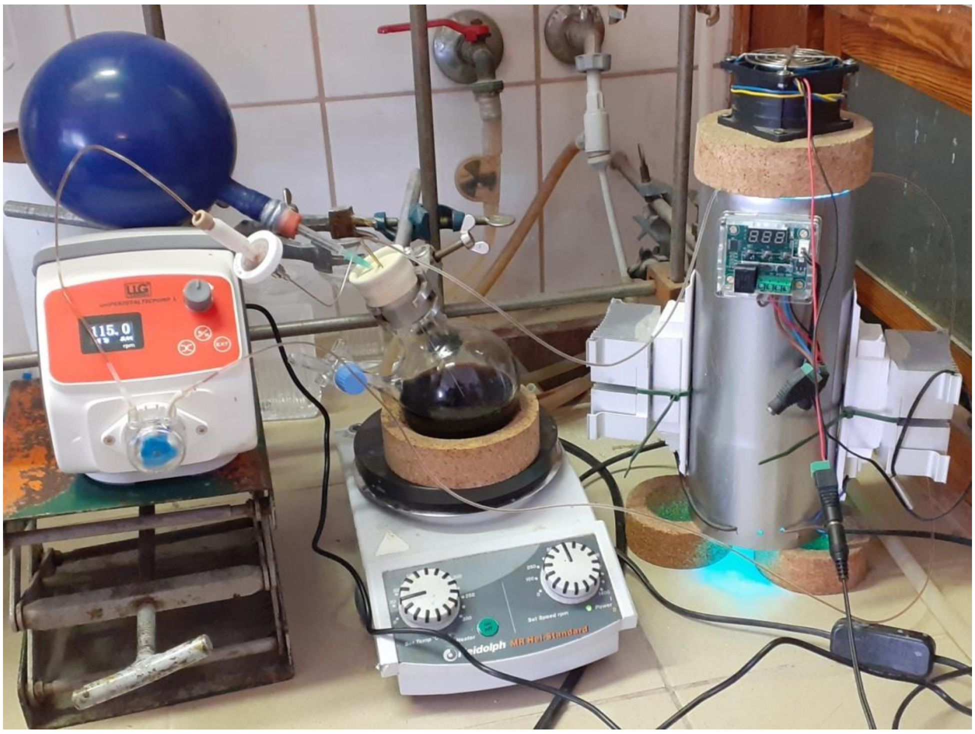

Picture S6. Self-made flow set

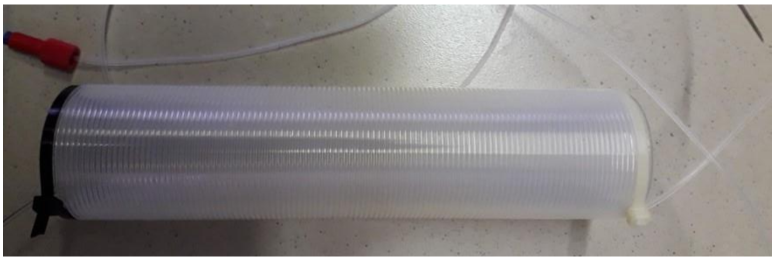

Picture S7. UV-transparent FEP tubing

General procedure:

Generation of imine: To a 50 mL round-bottom flask, 4-methoxybenzaldehyde (10 mmol, 1.217 mL), aniline (10 mmol, 913 µL), and HFIP (1,1,1,3,3,3-hexafluoroisopropanol) (10 mL) were added, and the reaction mixture was stirred overnight at 60°C using heating mantle (Picture S8).

Oxidative Meinwald-type rearrangement sequence: After cooling to room temperature, UHP (urea-hydrogen peroxide) (2.0 equiv., 20 mmol, 1.88 g) was added, and the reaction was left for 2 days at 45°C using heating mantle (Picture S9).

Photochemical deformylation reaction: HFIP was removed by distillation under reduced pressure (33°C, 186 mmHg) (Picture S10). A total of 9.6 mL of HFIP was recovered (Picture

S11). Then, to the crude reaction mixture, Et_2O (40 mL) was added (Picture S12). After stirring for 15 minutes, the resulting precipitate was filtered off using a Schott funnel and washed three times with Et_2O (10 mL each)(Picture S13). A total of 1.745 g of UHP residue was obtained. The combined filtrate was concentrated under reduced pressure on a rotary evaporator. The resulting crude mixture was dissolved in MeCN and transferred to a 250 mL round-bottom Schlenk flask. The solution was then degassed according to the procedure (see ref.[2])(Picture S14 and S15). The flask was connected to the flow set (a filter was installed between the vessel with the solution and the pump) and pumping of the solution was started (5 mL/min). After 5 minutes required to stabilize the flow, the UV-C lamps (4x9W) were switched on simultaneously. The progress of the reaction was monitored by TLC and ^{1}H-NMR. After complete conversion of *N*-(4-methoxyphenyl)-*N*-phenylformamide (3 days), the reaction was stopped by turning off the lamps. The reaction mixture was collected in a flask, the flow set was rinsed with 20 mL of MeCN and 20 mL of MeOH, combined solutions were concentrated, and the crude product was preabsorbed onto silica gel, then purified by column chromatography to yield 1.076 g (54%) of 4-methoxy-*N*-phenylaniline **3b**.

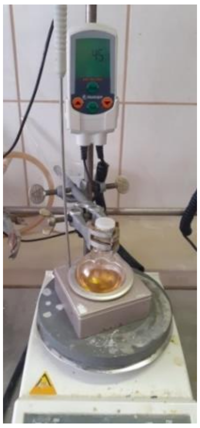

Picture S8.

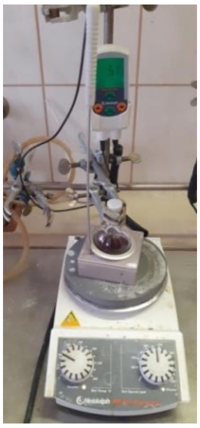

Picture S9.

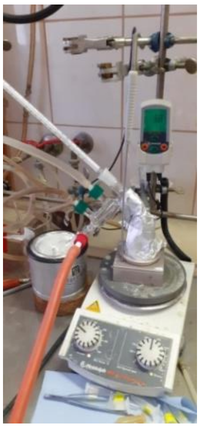

Picture S10.

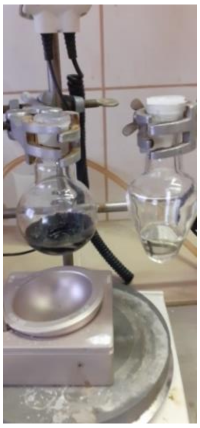

Picture S11.

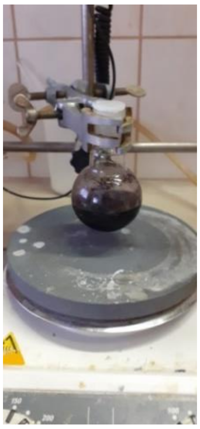

Picture S12.

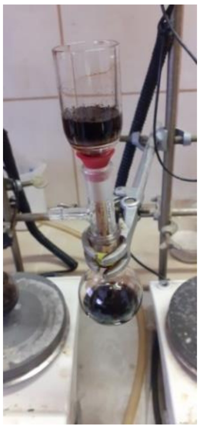

Picture S13.

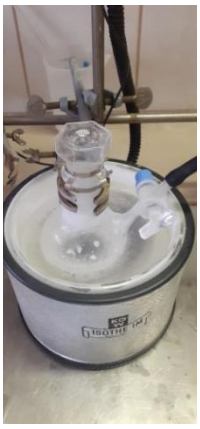

Picture S14.

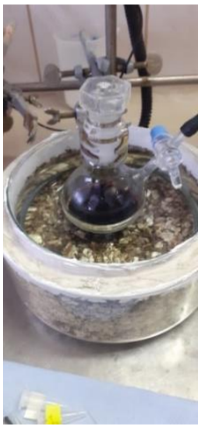

Picture S15.

6. **Characterization Data:**

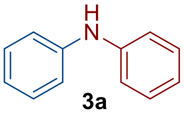

3a

Diphenylamine; 38% (16 mg obtained from 22 µL of aniline and 25 µL of benzaldehyde; white solid; m.p. 53-54°C; ^{1}H NMR (400 MHz, Chloroform-*d*) δ 7.32 – 7.25 (m, 4H), 7.11 – 7.06 (m, 4H), 6.99 – 6.90 (m, 2H), 5.69 (bs, 1H); ^{13}C{1H} NMR (101 MHz, Chloroform-*d*) δ 143.2, 129.4, 121.0, 117.9; The spectroscopic data are in agreement with literature data.[3]

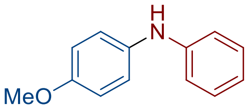

3b

4-Methoxy-*N*-phenylaniline; 57% (28 mg obtained from 22 µL of aniline and 30 µL of 4-methoxybenzaldehyde); colourless oil; ^{1}H NMR (400 MHz, Chloroform-*d*) δ 7.25 – 7.19 (m, 2H), 7.11 – 7.06 (m, 2H), 6.94 – 6.91 (m, 2H), 6.89 – 6.86 (m, 2H), 6.86 – 6.82 (m, 2H), 5.49 (bs, 1H), 3.81 (s, 3H); ^{13}C{1H} NMR (101 MHz, Chloroform-*d*) δ 155.3, 145.2, 135.8, 129.3, 122.2, 119.6, 115.7, 114.7, 55.6; The spectroscopic data are in agreement with literature data.[4]

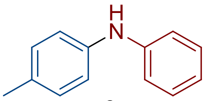

3c

4-Methyl-*N*-phenylaniline; 55% (100 mg obtained from 100 µL of aniline and 118 µL of 4-methylbenzaldehyde); orange oil; ^{1}H NMR (400 MHz, Chloroform-*d*) δ 7.34 – 7.26 (m, 2H), 7.16 – 7.12 (m, 2H), 7.10 – 7.04 (m, 4H), 6.94 (t, *J* = 7.3 Hz, 1H), 5.62 (bs, 1H), 2.37 (s, 3H); ^{13}C{1H} NMR (101 MHz, Chloroform-*d*) δ 144.0, 140.3, 130.9, 129.9, 129.3, 120.3, 119.0, 116.9, 20.7; The spectroscopic data are in agreement with literature data.[4]

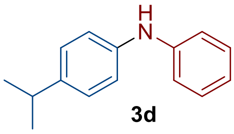

3d

4-Isopropyl-*N*-phenylaniline; 59% (125 mg obtained from 100 µL of aniline and 151 µL of 4-isopropylbenzaldehyde); orange waxy solid; ^{1}H NMR (400 MHz, Chloroform-*d*) δ 7.27 – 7.23 (m, 2H), 7.17 – 7.13 (m, 2H), 7.06 – 7.02 (m, 4H), 6.92 – 6.87 (m, 1H), 5.62 (bs, 1H), 2.88 (hept, *J* = 6.9 Hz, 1H), 1.26 (d, *J* = 6.9 Hz, 6H); ^{13}C{1H} NMR (101 MHz, Chloroform-*d*) δ 143.9,

142.1, 140.6, 129.3, 127.2, 120.4, 118.7, 117.0, 33.4, 24.1. The spectroscopic data are in agreement with literature data.[3]

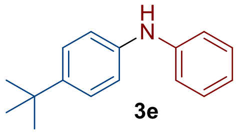

3e

4-(*tert*-butyl)-*N*-phenylaniline; 53% (119 mg obtained from 100 µL of aniline and 167 µL of 4-*tert*-butylbenzaldehyde); orange waxy solid; ^{1}H NMR (400 MHz, Chloroform-*d*) δ 7.37 – 7.32 (m, 2H), 7.32 – 7.24 (m, 2H), 7.10 – 7.05 (m, 4H), 6.93 (t, *J* = 7.3 Hz, 1H), 5.64 (bs, 1H), 1.36 (s, 9H); ^{13}C{1H} NMR (101 MHz, Chloroform-*d*) δ 144.2, 143.8, 140.4, 129.3, 126.2, 120.4, 118.2, 117.2, 34.2, 31.5. The spectroscopic data are in agreement with literature data.[5]

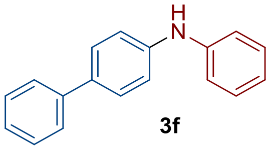

3f

***N*-Phenyl-4-biphenlyamine;** 69% (169 mg obtained from 100 µL of aniline and 167 µL of 4-phenylbenzaldehyde); yellow solid, m.p. 112-113 °C; ^{1}H NMR (400 MHz, Chloroform-*d*) δ 7.72 – 7.67 (m, 2H), 7.64 – 7.60 (m, 2H), 7.52 (t, *J* = 7.7 Hz, 2H), 7.44 – 7.36 (m, 3H), 7.24 – 7.18 (m, 4H), 7.07 (t, *J* = 7.3 Hz, 1H), 5.79 (s, 1H); ^{13}C NMR (101 MHz, Chloroform-*d*) δ 143.0, 142.7, 141.0, 133.8, 129.5, 128.9, 128.1, 126.73, 126.65, 121.4, 118.2, 118.0; The spectroscopic data are in agreement with literature data.[3]

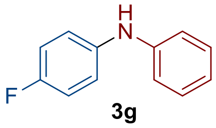

3g

4-Fluoro-*N*-phenylaniline; 51% (95 mg obtained from 100 µL of aniline and 107 µL of 4-fluorobenzaldehyde); orange oil; ^{1}H NMR (400 MHz, Chloroform-*d*) δ 7.31 – 7.25 (m, 2H), 7.08 – 6.92 (m, 7H), 5.57 (bs, 1H); ^{13}C{1H} NMR (101 MHz, Chloroform-*d*) δ 158.1 (d, *J* = 240.0 Hz), 144.0, 139.0 (d, *J* = 2.5 Hz), 129.4, 120.7, 120.6, 116.9, 116.0 (d, *J* = 22.5 Hz); ^{19}F NMR (376 MHz, Chloroform-*d*) δ -121.9; The spectroscopic data are in agreement with literature data.[6]

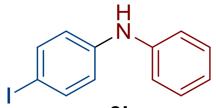

3h

4-Iodo-*N*-phenylaniline; 18% (53 mg obtained from 100 µL of aniline and 232 mg of 4-iodobenzaldehyde); red oil; ^{1}H NMR (400 MHz, Chloroform-*d*) δ 7.54 – 7.49 (m, 2H), 7.30 – 7.26 (m, 2H), 7.08 – 7.03 (m, 2H), 6.99 – 6.94 (m, 1H), 6.86 – 6.79 (m, 2H), 5.67 (bs, 1H); ^{13}C{1H} NMR (101 MHz, $CDCl_3$) δ 143.2, 142.2, 138.1, 129.4, 121.8, 119.3, 118.6, 82.1; The spectroscopic data are in agreement with literature data.[3]

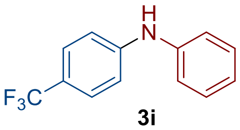

3i

***N*-Phenyl-4-(trifluoromethyl)aniline;** 7% (12 mg, obtained from 100 µL of aniline and 137 µL of 4-(trifluoromethyl)benzaldehyde); yellow oil; ^{1}H NMR (600 MHz, Chloroform-*d*) δ 7.46 (d, *J* = 8.4 Hz, 2H), 7.37 – 7.30 (m, 2H), 7.14 (d, *J* = 7.5 Hz, 2H), 7.09 – 7.02 (m, 3H), 5.90 (bs, 1H); ^{13}C NMR (151 MHz, Chloroform-*d*) δ 146.8, 141.1, 129.5, 126.7 (q, J = 3.9 Hz), 124.6 (q, J = 270.7 Hz), 122.9, 121.7 (q, J = 32.7 Hz), 120.0, 115.3; ^{19}F NMR (376 MHz, Chloroform-*d*) δ -61.5; The spectroscopic data are in agreement with literature data.[6]

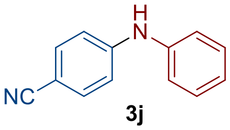

3j

4-(Phenylamino)benzonitrile; 11% (21 mg obtained from 100 µL of aniline and 131 mg of 4-formylbenzonitrile); orange oil; ^{1}H NMR (400 MHz, Chloroform-*d*) δ 7.49 – 7.46 (m, 2H), 7.38 – 7.33 (m, 2H), 7.18 – 7.15 (m, 2H), 7.14 – 7.09 (m, 1H), 7.00 – 6.94 (m, 2H), 6.06 (bs, 1H); ^{13}C{1H} NMR (101 MHz, Chloroform-*d*) δ 148.0, 140.0, 133.8, 129.6, 124.0, 121.3, 119.8, 114.9, 101.6; The spectroscopic data are in agreement with literature data.[3]

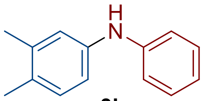

3k

3,4-Dimethyl-*N*-phenylaniline; 49% (97 mg obtained from 100 µL of aniline and 133 ul of 3,4-dimethylbenzaldehyde); red oil; ^{1}H NMR (400 MHz, Chloroform-*d*) δ 7.33 – 7.26 (m, 2H), 7.10 – 7.04 (m, 3H), 6.96 – 6.90 (m, 3H), 5.61 (s, 1H), 2.30 – 2.25 (m, 6H); ^{13}C{1H} NMR (101 MHz, Chloroform-*d*) δ 144.1, 140.7, 137.6, 130.4, 129.3, 120.4, 120.2, 117.0, 116.3, 20.0, 19.0; The spectroscopic data are in agreement with literature data.[7]

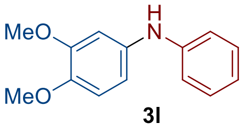

3l

3,4-Dimethoxy-*N*-phenylaniline; 59% (136 mg obtained from 100 μL of aniline and 166 mg of 3,4-dimethoxybenzaldehyde); yellow solid; m.p. 100-101°C; ^{1}H NMR (400 MHz, Chloroform-*d*) δ 7.25 – 7.20 (m, 2H), 6.96 – 6.92 (m, 2H), 6.87 – 6.83 (m, 1H), 6.81 (d, *J* = 8.5 Hz, 1H), 6.72 (d, *J* = 2.5 Hz, 1H), 6.67 (dd, *J* = 8.5, 2.5 Hz, 1H), 5.51 (s, 1H), 3.86 (s, 3H), 3.84 (s, 3H); ^{13}C{1H} NMR (101 MHz, Chloroform-*d*) δ 149.7, 144.9, 144.7, 136.3, 129.3, 119.8, 116.0, 112.23, 112.21, 105.4, 56.3, 55.9; The spectroscopic data are in agreement with literature data.[8]

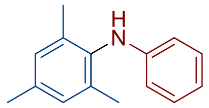

3m

2,4,6-Trimethyl-*N*-phenylaniline; 17% (35 mg obtained from 100 μL of aniline and 148 ul of 2,4,6-trimethylbenzaldehyde); orange oil; ^{1}H NMR (400 MHz, Chloroform-*d*) δ 7.19 – 7.11 (m, 2H), 6.96 – 6.93 (m, 2H), 6.73 (t, *J* = 7.3 Hz, 1H), 6.53 – 6.46 (m, 2H), 5.09 (bs, 1H), 2.32 (s, 3H), 2.19 (s, 6H); ^{13}C{1H} NMR (101 MHz, Chloroform-*d*) δ 146.7, 136.0, 135.5, 135.4, 129.2, 129.1, 117.9, 113.3, 20.9, 18.2; The spectroscopic data are in agreement with literature data.[4]

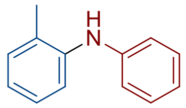

3n

2-Methyl-*N*-phenylaniline; 64% (117 mg obtained from 100 μL of aniline and 116 μL of 2-methylbenzaldehyde); orange oil; ^{1}H NMR (400 MHz, Chloroform-*d*) δ 7.34 – 7.24 (m, 4H), 7.23 – 7.17 (m, 1H), 7.03 – 6.92 (m, 4H), 5.41 (bs, 1H), 2.31 (s, 3H); ^{13}{1H} NMR (101 MHz, Chloroform-*d*) δ 144.1, 141.3, 131.0, 129.4, 128.5, 126.8, 122.1, 120.5, 119.0, 117.5, 17.9; The spectroscopic data are in agreement with literature data.[4]

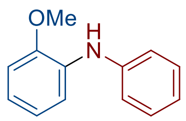

3o

2-Methoxy-*N*-phenylaniline; 55% (110 mg obtained from 100 μL of aniline and 136 mg of 2-methoxybenzaldehyde); orange oil; ^{1}H NMR (400 MHz, Chloroform-*d*) δ 7.42 – 7.34 (m, 3H), 7.25 – 7.22 (m, 2H), 7.05 – 7.00 (m, 1H), 6.99 – 6.94 (m, 3H), 6.24 (bs, 1H), 3.94 (s, 3H); ^{13}C{1H} NMR (101 MHz, Chloroform-*d*) δ 148.4, 142.9, 133.1, 129.4, 121.2, 120.9, 120.0, 118.7, 114.8, 110.7, 55.7; The spectroscopic data are in agreement with literature data.[3]

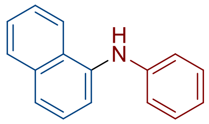

3p

***N*-Phenylnaphthalen-1-amine**; 54% (118 mg obtained from 100 µL of aniline and 136 µL mg of 1-naphthaldehyde); orange oil; ^{1}H NMR (400 MHz, Chloroform-*d*) δ 8.07 – 8.00 (m, 1H), 7.89 (dd, *J* = 7.4, 2.0 Hz, 1H), 7.59 (dd, *J* = 7.7, 1.8 Hz, 1H), 7.50 (pd, *J* = 6.8, 1.6 Hz, 2H), 7.44 – 7.37 (m, 2H), 7.31 – 7.25 (m, 2H), 7.01 (d, *J* = 7.4 Hz, 2H), 6.94 (t, *J* = 7.3 Hz, 1H), 5.94 (bs, 1H); ^{13}C{1H} NMR (101 MHz, Chloroform-*d*) δ 144.8, 138.8, 134.7, 129.4, 128.6, 127.8, 126.1, 126.0, 125.7, 123.0, 121.8, 120.5, 117.4, 115.9; The spectroscopic data are in agreement with literature data.[8]

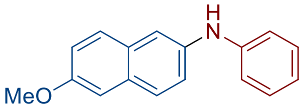

3r

6-Methoxy-*N*-phenylnaphthalen-2-amine; 41% (103 mg obtained from 100 µL of aniline and 186 mg of 6-methoxy-2-naphthaldehyde); orange waxy solid; ^{1}H NMR (400 MHz, Chloroform-*d*) δ 7.67 (d, *J* = 8.7 Hz, 1H), 7.59 (d, *J* = 8.8 Hz, 1H), 7.43 (d, *J* = 2.3 Hz, 1H), 7.33 – 7.27 (m, 2H), 7.24 (dd, *J* = 8.7, 2.4 Hz, 1H), 7.15 – 7.09 (m, 4H), 6.96 (t, *J* = 7.3 Hz, 1H), 5.78 (bs, 1H), 3.92 (s, 3H); ^{13}C{1H} NMR (101 MHz, $CDCl_3$) δ 156.3, 143.7, 138.8, 130.3, 129.9, 129.4, 128.1, 127.9, 121.2, 120.8, 119.1, 117.4, 113.5, 106.1, 55.3; The spectroscopic data are in agreement with literature data.[9]

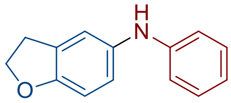

3s

***N*-Phenyl-2,3-dihydrobenzofuran-5-amine**; 48% (101 mg obtained from 100 µL of aniline and 126 µL of 2,3-dihydrobenzofuran-5-carbaldehyde); yellow waxy solid; ^{1}H NMR (400 MHz, Chloroform-*d*) δ 7.24 – 7.18 (m, 2H), 7.03 – 7.02 (m, 1H), 6.91 – 6.86 (m, 3H), 6.85 – 6.80 (m, 1H), 6.74 (d, *J* = 8.4 Hz, 1H), 5.44 (bs, 1H), 4.57 (t, *J* = 8.6 Hz, 2H), 3.19 (t, *J* = 8.7 Hz, 2H); ^{13}C{1H} NMR (101 MHz, Chloroform-*d*) δ 156.1, 145.8, 135.5, 129.3, 128.0, 121.6, 119.3, 119.0, 115.4, 109.5, 71.3, 30.1.

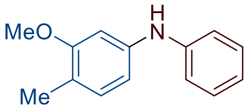

3t

3-Methoxy-4-methyl-*N*-phenylaniline; 76% (162 mg obtained from 90 µL of aniline and 150 mg of 3-methoxy-4-methylbenzaldehyde); orange solid; m.p. 81-82 °C; ^{1}H NMR (400 MHz, Chloroform-*d*) δ 7.35 – 7.28 (m, 2H), 7.13 – 7.05 (m, 3H), 7.00 – 6.89 (m, 1H), 6.73 – 6.57 (m, 2H), 5.68 (bs, 1H), 3.82 (s, 3H), 2.24 (s, 3H); ^{13}C{1H} NMR (101 MHz, Chloroform-*d*) δ 158.4, 144.0, 142.0, 131.0, 129.4, 120.4, 119.8, 117.2, 110.5, 101.8, 55.3, 15.7; The spectroscopic data are in agreement with literature data.[10]

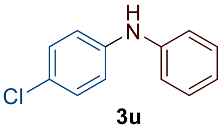

3u

4-Chloro-*N*-phenylaniline; 35%; (71 mg obtained from 91 µL of aniline and 140 mg of 4-chlorobenzaldehyde); waxy solid; ^{1}H NMR (400 MHz, Chloroform-*d*) δ 7.33 – 7.26 (m, 2H), 7.24 – 7.18 (m, 2H), 7.08 – 7.03 (m, 2H), 7.02 – 6.94 (m, 3H), 5.67 (bs, 1H); ^{13}C{1H} NMR (101 MHz, Chloroform-*d*) δ 142.7, 141.9, 129.5, 129.3, 125.5, 121.5, 118.8, 118.1; The spectroscopic data are in agreement with literature data.[11]

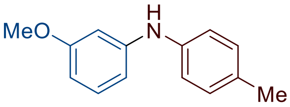

3w

3-Methoxy-*N*-(p-tolyl)aniline; 43% (92 mg obtained from 107 mg of *p*-toluidine and 136 mg of 3-methoxybenzaldehyde); waxy solid; ^{1}H NMR (400 MHz, Chloroform-*d*) δ 7.21 – 7.12 (m, 1H), 7.12 (d, *J* = 8.1 Hz, 2H), 7.04 (d, *J* = 8.4 Hz, 2H), 6.64 – 6.59 (m, 2H), 6.47 (ddd, *J* = 8.1, 2.8, 1.5 Hz, 1H), 5.63 (bs, 1H), 3.79 (s, 3H), 2.34 (s, 3H); ^{13}C{1H} NMR (101 MHz, Chloroform-*d*) δ 160.8, 145.5, 140.0, 131.2, 130.1, 129.9, 119.4, 109.4, 105.5, 102.4, 55.2, 20.7; The spectroscopic data are in agreement with literature data.[12]

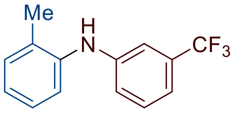

3x

2-Methyl-*N*-(3-(trifluoromethyl)phenyl)aniline; 26% (65 mg obtained from 125 µL of 3-(trifluoromethyl)aniline and 116 µL of 2-methylbenzaldehyde); yellow oil; ^{1}H NMR (400 MHz, Chloroform-*d*) δ 7.38 – 7.31 (m, 1H), 7.29 – 7.18 (m, 3H), 7.16 – 7.10 (m, 2H), 7.10 – 7.02 (m, 2H), 5.53 (s, 1H), 2.28 (s, 3H); ^{13}C{1H} NMR (101 MHz, Chloroform-*d*) δ 145.1, 139.8, 131.3,

130.2, 129.8, 127.0, 123.7, 120.9, 119.1, 116.24 (q, *J* = 3.9 Hz), 112.62 (q, *J* = 3.9 Hz), 17.9; ^{19}F NMR (376 MHz, Chloroform-*d*) δ -62.77; The spectroscopic data are in agreement with literature data.[13]

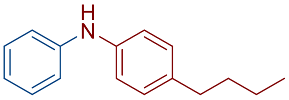

3y

4-Butyl-*N*-phenylaniline; 19% (43 mg obtained from 102 µL of benzaldehyde and 158 µL of 4-butylaniline); orange oil; ^{1}H NMR (400 MHz, Chloroform-*d*) δ 7.27 – 7.21 (m, 2H), 7.11 – 7.07 (m, 2H), 7.04 – 7.00 (m, 4H), 6.88 (tt, *J* = 7.3, 1.2 Hz, 1H), 5.60 (bs, 1H), 2.59 – 2.54 (m, 2H), 1.63 – 1.55 (m, 2H), 1.44 – 1.30 (m, 2H), 0.94 (t, *J* = 7.3 Hz, 3H); $^{13}C\{1H\}$ NMR (101 MHz, Chloroform-*d*) δ 143.9, 140.5, 136.1, 129.3, 129.2, 120.3, 118.7, 117.0, 34.9, 33.8, 22.3, 14.0; The spectroscopic data are in agreement with literature data.[14]

7. Synthesis of Phentolamine.

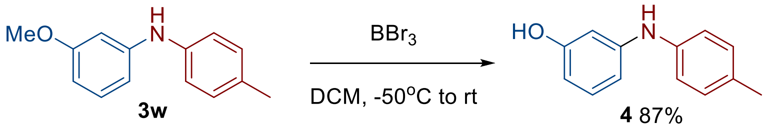

To a solution of compound **3w** (167 mg, 0.78 mmol) in dichloromethane (8 mL) was added slowly a solution of BBr_3 in dichloromethane (1.6 mL, 1.0 M in CH_2Cl_2, 1.6 mmol) at −50°C. The resulting solution was warmed to r.t. over 2 h. Saturated aqueous sodium bicarbonate (10 mL) was added at 0°C. The solution was extracted with dichloromethane (3x10 mL). The combined organic layer was dried over anhydrous Na_2SO_4, filtered, and concentrated in vacuum. The residue was purified by flash chromatography on silica gel to afford 135 mg of 3-(p-tolylamino)phenol **4** (87%) as waxy solid; ^{1}H NMR (400 MHz, Chloroform-*d*) δ 7.15 – 7.09 (m, 3H), 7.02 (d, *J* = 8.1 Hz, 2H), 6.59 (dd, *J* = 8.1, 2.2 Hz, 1H), 6.50 (s, 1H), 6.37 (dd, *J* = 8.0, 2.4 Hz, 1H), 5.53 (bs, 1H), 2.35 (s, 3H); $^{13}C\{1H\}$ NMR (101 MHz, Chloroform-*d*) δ 156.5, 145.8, 139.8, 131.5, 130.4, 129.9, 119.8, 109.3, 107.2, 103.4, 20.8; The spectroscopic data are in agreement with literature data.[15]

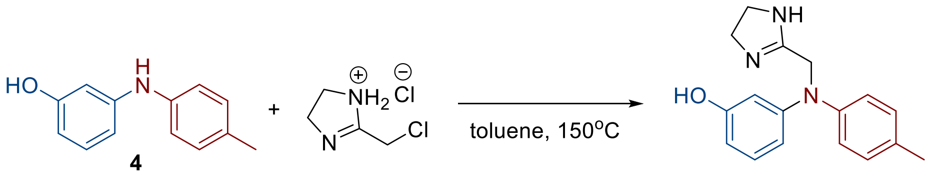

Phentolamine 49% +
32% substrate **4** recovery

A mixture of compound **4** (98 mg, 0.49 mmol) and 2-(chloromethyl)-1H-imidazole hydrochloride (76 mg, 0.49 mmol, 1.0 equiv.) in toluene (2 mL) was heated in a sealed ampule at 150°C for 7 hours. After cooling to 100°C, the reaction mixture was diluted with ethyl acetate (5 mL) and water (5 mL). Following phase separation, the aqueous layer was treated with a 25% aqueous solution of ammonia and stirred for 15 minutes. The resulting mixture was extracted with dichloromethane (3×5 mL). The combined organic layers were dried over anhydrous Na_2SO_4, filtered, and concentrated under reduced pressure. The crude product was purified by flash chromatography on silica gel using a 1:9 mixture of MeOH/DCM containing 1% of 25% aqueous ammonia to afford **phentolamine** (68 mg, 49%) as a white solid; m.p. 172-173°C; ^{1}H NMR (500 MHz, DMSO-d_6) δ 7.13 (d, J = 8.4 Hz, 2H), 7.07 (d, J = 8.4 Hz, 2H), 6.96 (t, J = 8.4 Hz, 1H), 6.30 – 6.24 (m, 3H), 4.35 (s, 2H), 3.47 (s, 4H), 2.27 (s, 3H); ^{13}C{1H} NMR (126 MHz, DMSO-d_6) δ 166.2, 158.0, 149.4, 144.8, 132.3, 129.8, 129.5, 123.6, 108.3, 107.1, 104.7, 79.2, 50.6, 48.4, 20.4. The spectroscopic data are in agreement with literature data.[15]

8. Reactivity of variously substituted anilines as coupling partners with benzaldehyde, unsuccessful results.

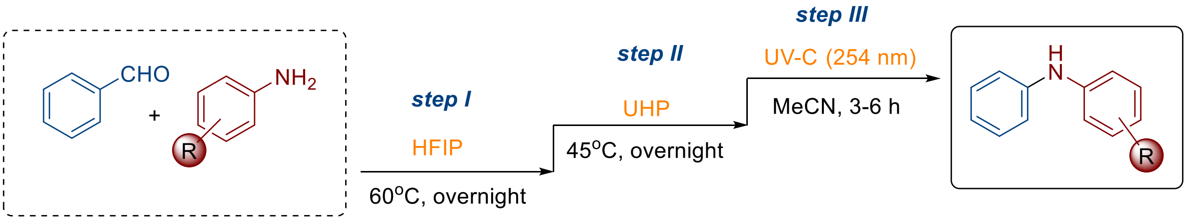

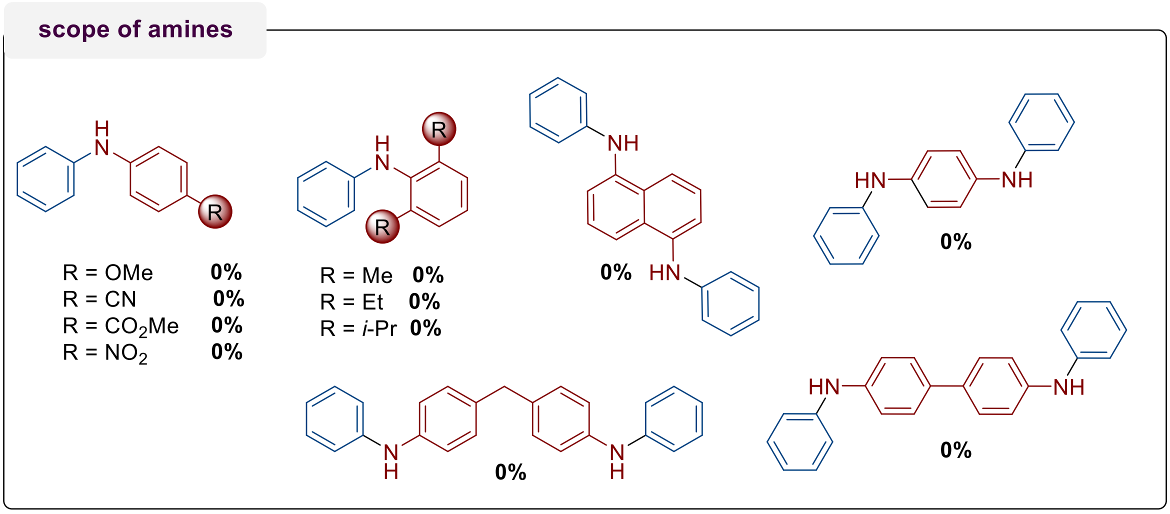

Table S1.

8. Literature:

1. Szcześniak, P.; Furman, B., Photo-Fries-type rearrangement of cyclic enamides. An efficient route to structurally diverse five-membered enaminones. *Chem. Commun.* **2022,** *58*, 1898-1901.
2. schlenklinesurvivalguide.com/freeze-pump-thaw/.
3. Yu, J.-f.; Wang, Y.; Zhang, P.; Wu, J., Direct Amination of Phenols under Metal-Free Conditions. *Synlett* **2013,** *24*, 1448 - 1454.
4. Yang, S.; Yu, X.; Poater, A.; Cavallo, L.; Cazin, C. S. J.; Nolan, S. P.; Szostak, M., Buchwald–Hartwig Amination and C–S/S–H Metathesis of Aryl Sulfides by Selective C–S Cleavage Mediated by Air- and Moisture-Stable [Pd(NHC)(μ-Cl)Cl]2 Precatalysts: Unified Mechanism for Activation of Inert C–S Bonds. *Org. Lett.* **2022,** *24*, 9210-9215.
5. Zhu, C.; Kale, A. P.; Yue, H.; Rueping, M., Redox-Neutral Cross-Coupling Amination with Weak N-Nucleophiles: Arylation of Anilines, Sulfonamides, Sulfoximines, Carbamates, and Imines via Nickelaelectrocatalysis. *JACS Au* **2021,** *1*, 1057-1065.
6. Luo, H.; Wang, G.; Feng, Y.; Zheng, W.; Kong, L.; Ma, Y.; Matsunaga, S.; Lin, L., Photoinduced Nickel-Catalyzed Carbon–Heteroatom Coupling**. *Chem. Eur. J.* **2023,** *29*, e202202385.
7. Rauser, M.; Eckert, R.; Gerbershagen, M.; Niggemann, M., Catalyst-Free Reductive Coupling of Aromatic and Aliphatic Nitro Compounds with Organohalides. *Angew. Chem. Int. Ed.* **2019,** *58*, 6713-6717.
8. Jati, A.; Dey, K.; Nurhuda, M.; Addicoat, M. A.; Banerjee, R.; Maji, B., Dual Metalation in a Two-Dimensional Covalent Organic Framework for Photocatalytic C–N Cross-Coupling Reactions. *J. Am. Chem. Soc.* **2022,** *144*, 7822-7833.
9. Mishra, A. K.; Verma, A.; Biswas, S., Nucleophilic ipso-Substitution of Aryl Methyl Ethers through Aryl C–OMe Bond Cleavage; Access to Functionalized Bisthiophenes. *J. Org. Chem.* **2017,** *82*, 3403-3410.
10. Rizos, S. R.; Ouzounthanasis, K. A.; Koumbis, A. E., Enantiospecific Total Synthesis and Absolute Configuration Assignment of Chabrolobenzoquinone H. *J. Org. Chem.* **2022,** *87*, 1313-1324.
11. Daneshvar, M. R.; Tavakolian, M.; Hosseini-Sarvari, M., Visible-Light-Responsive Nano CuO/ZnO Photocatalyst for Chan–Lam Coupling Reaction and Aerobic C(sp3)–H Bond Oxidation. *Synthesis* **2023,** *55*, 2495-2502.
12. Hajra, A.; Wei, Y.; Yoshikai, N., Palladium-Catalyzed Aerobic Dehydrogenative Aromatization of Cyclohexanone Imines to Arylamines. *Organic Letters* **2012,** *14*, 5488-5491.
13. Wang, H.; Li, F.; Yang, W.; Wang, Y.; Miskevich, A. A.; Loiko, V. A.; Zhang, L.; Tao, S., Impact of Adding N-hexylamine to Nickel Metallophotoredox C–N Coupling to Form Diarylamines. *J. Org. Chem.* **2025,** *90*, 1233-1244.
14. Zielińska, A. A.; Trzaska, P.; Budny, M.; Bosiak, M. J., Kumada–Tamao–Corriu Type Reaction of Aromatic Bromo- and Iodoamines with Grignard Reagents. *J. Org. Chem.* **2023,** *88*, 16167-16175.
15. Roscales, S.; Csákÿ, A. G., Synthesis of Di(hetero)arylamines from Nitrosoarenes and Boronic Acids: A General, Mild, and Transition-Metal-Free Coupling. *Org. Lett.* **2018,** *20*, 1667-1671.

^{1}H NMR (400 MHz, Chloroform-*d*)

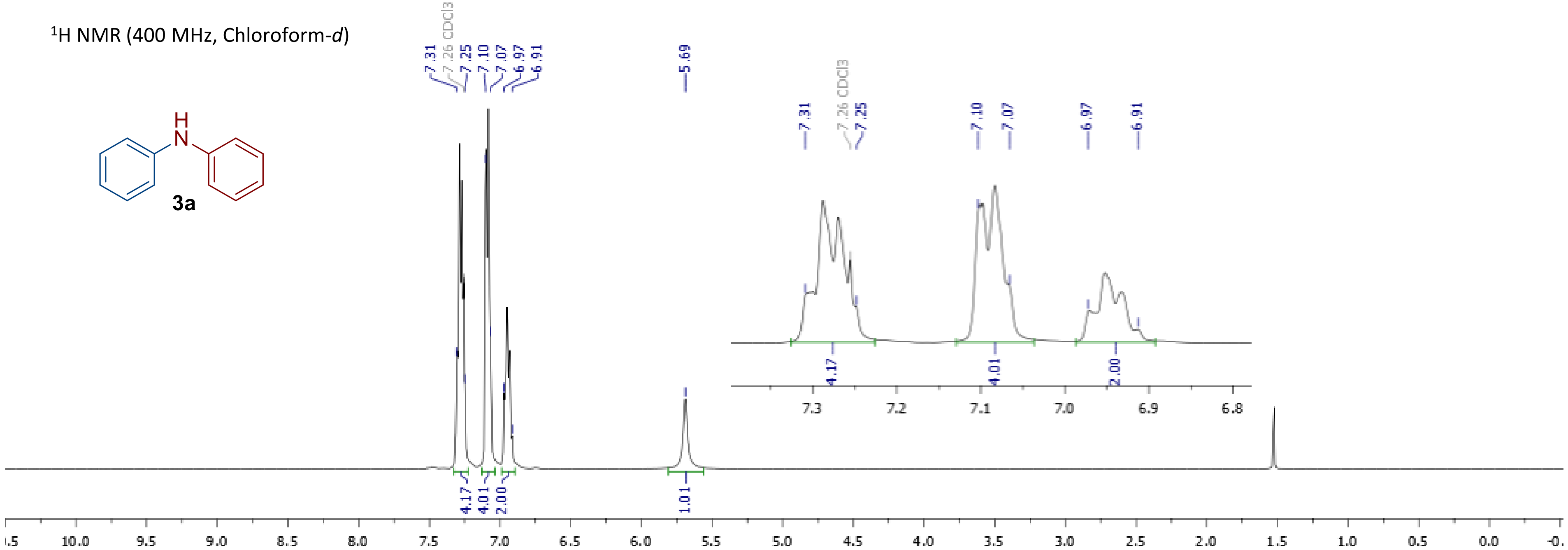

$^{13}C\{1H\}$ NMR (101 MHz, Chloroform-*d*)

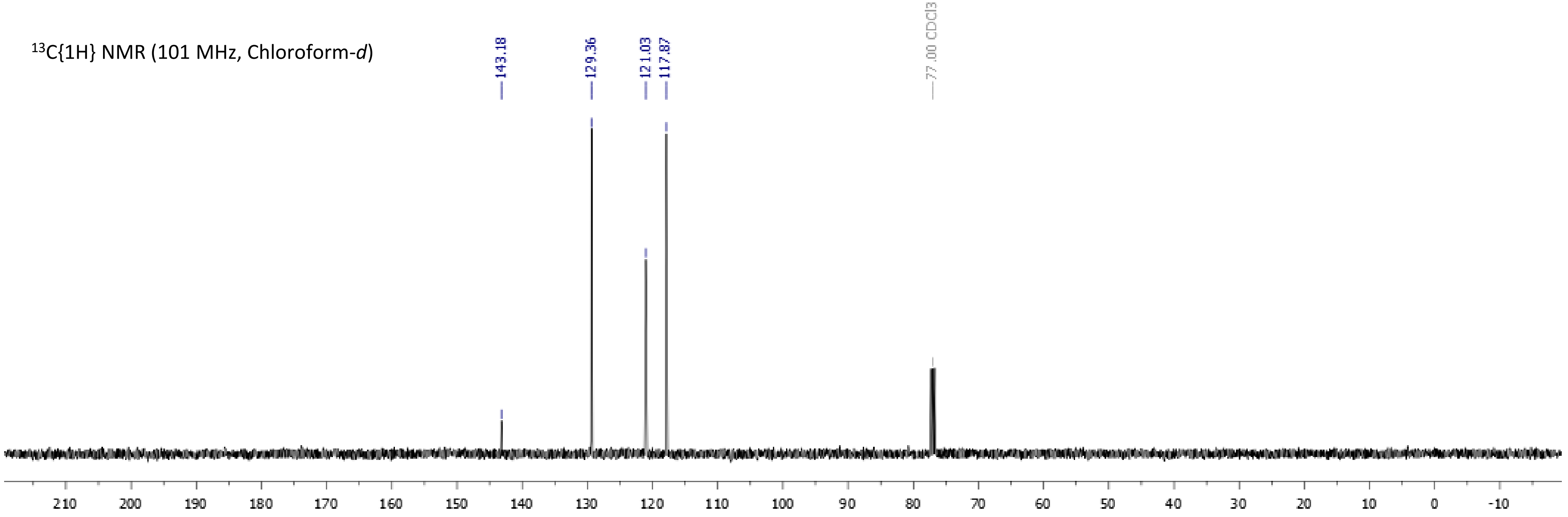

^{1}H NMR (400 MHz, Chloroform-*d*)

3b

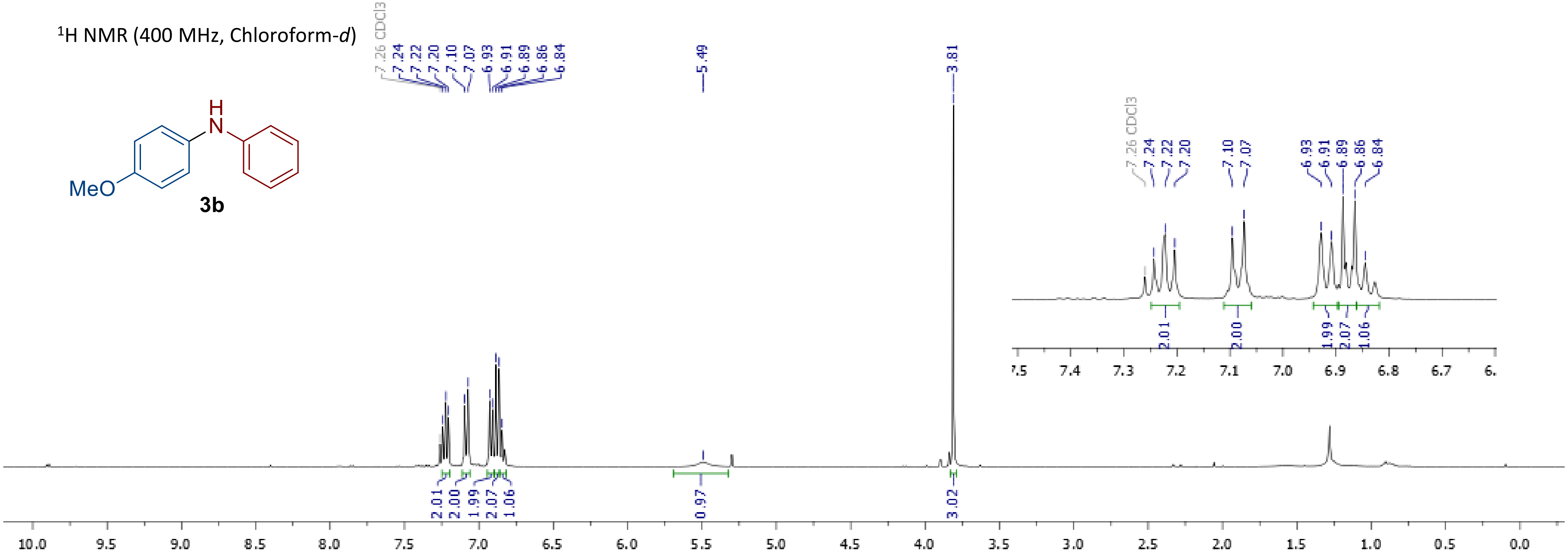

^{13}C{1H} NMR (101 MHz, Chloroform-*d*)

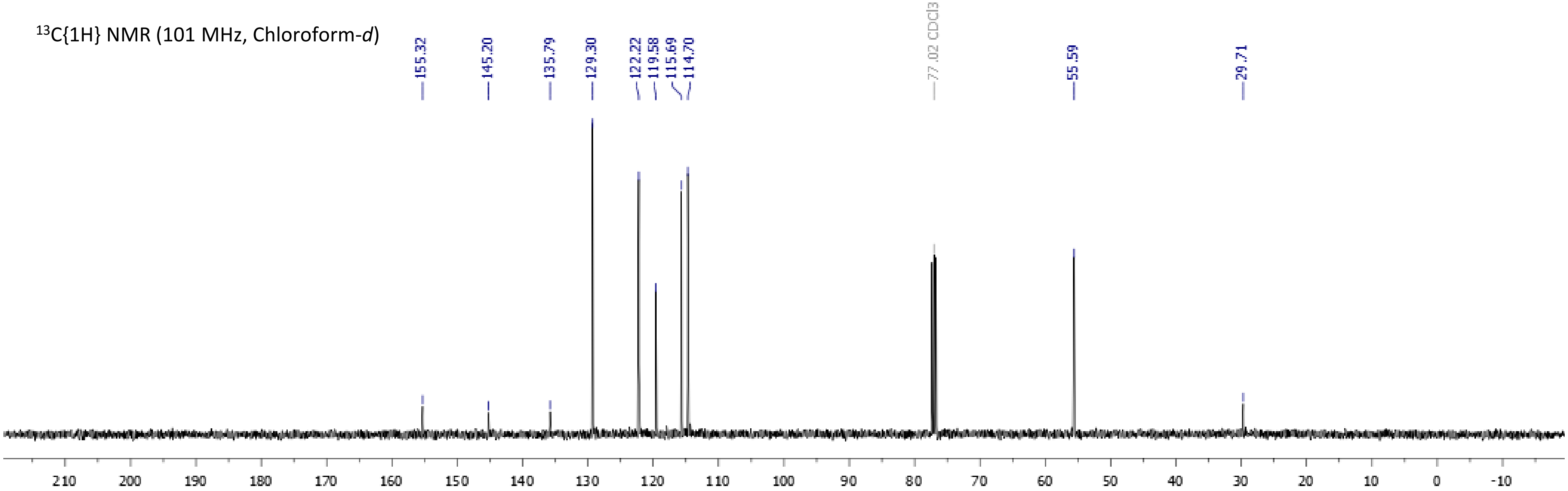

¹H NMR (400 MHz, Chloroform-*d*)

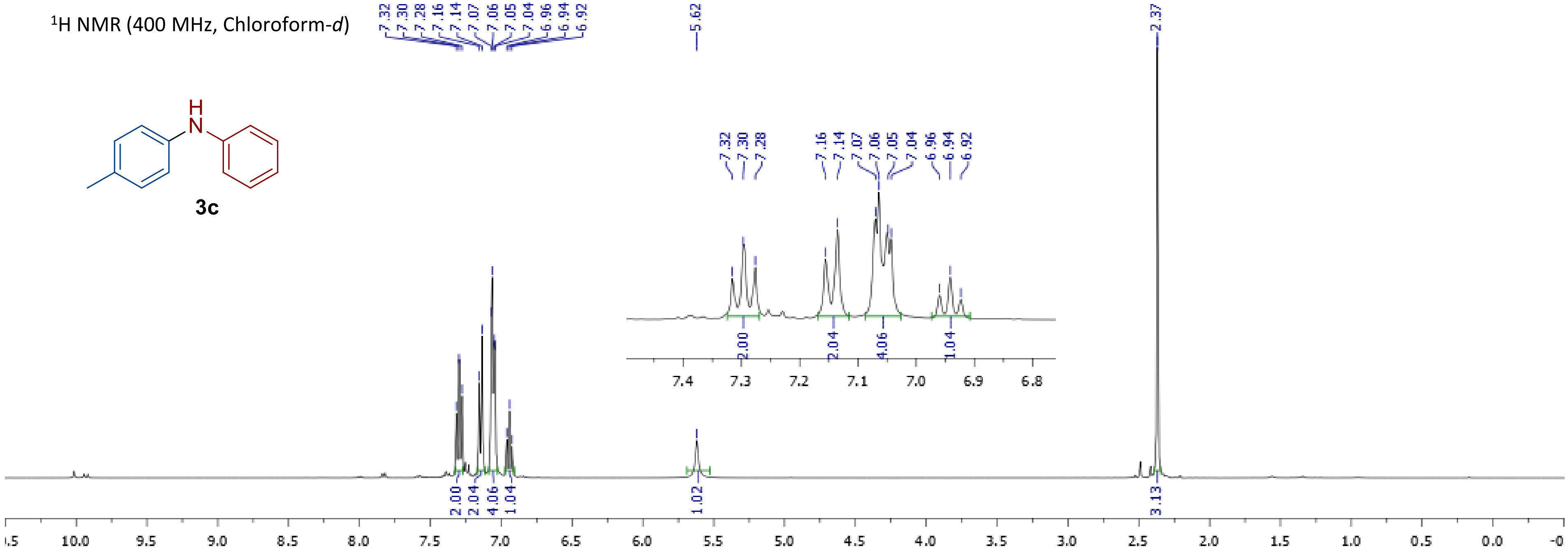

$^{13}C\{1H\}$ NMR (101 MHz, Chloroform-*d*)

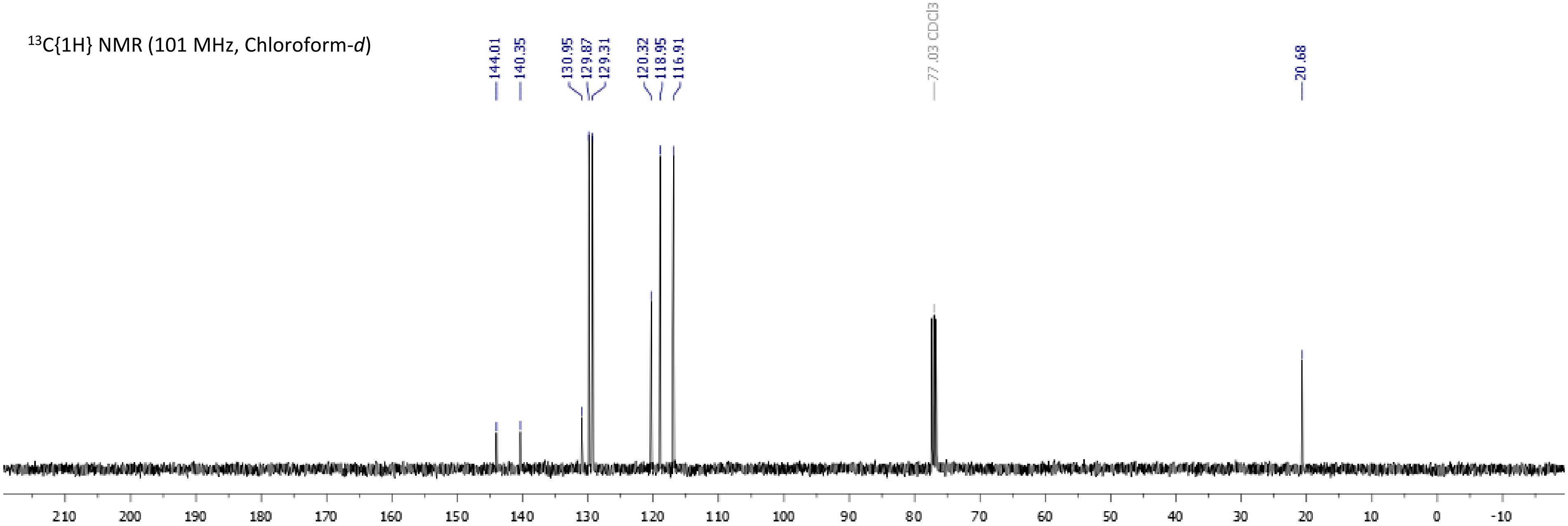

^{1}H NMR (400 MHz, Chloroform-*d*)

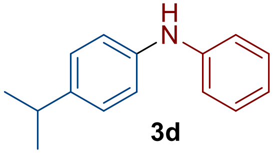

3d

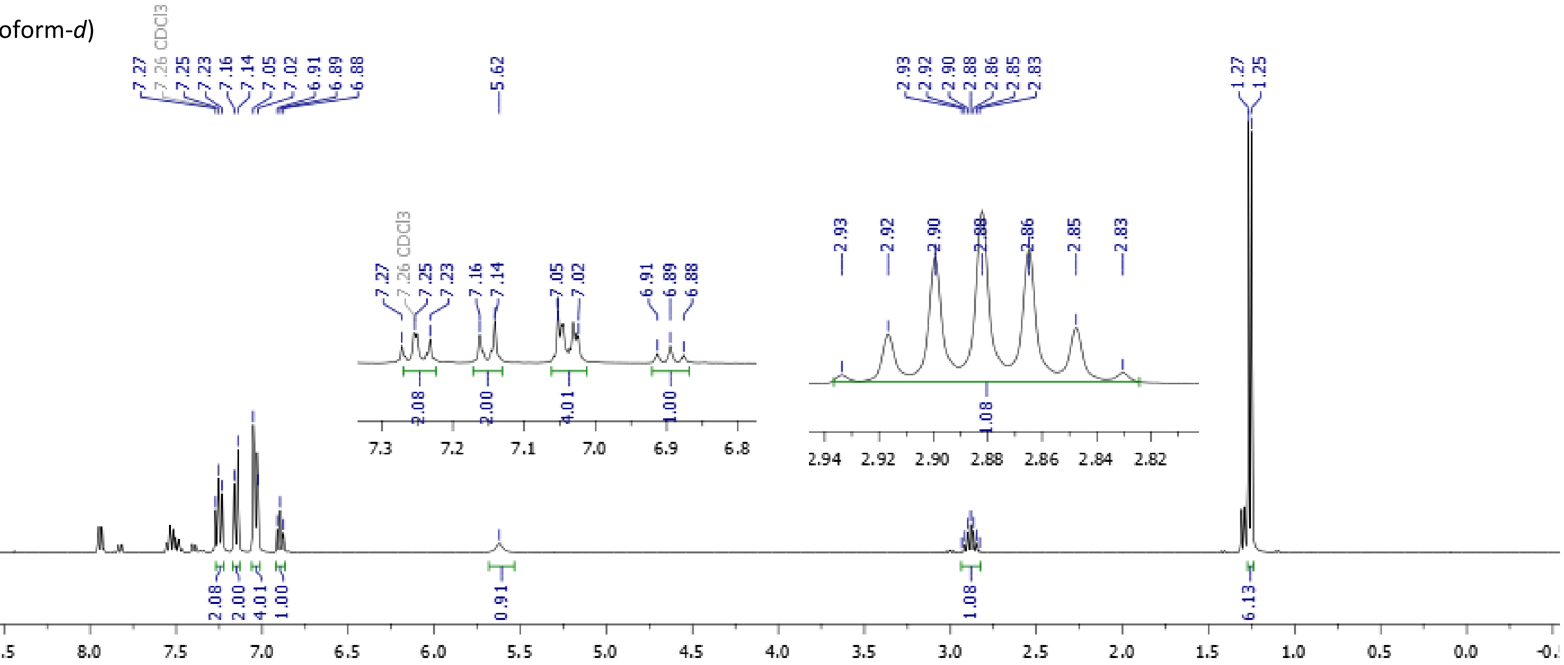

^{13}C{1H} NMR (101 MHz, Chloroform-*d*)

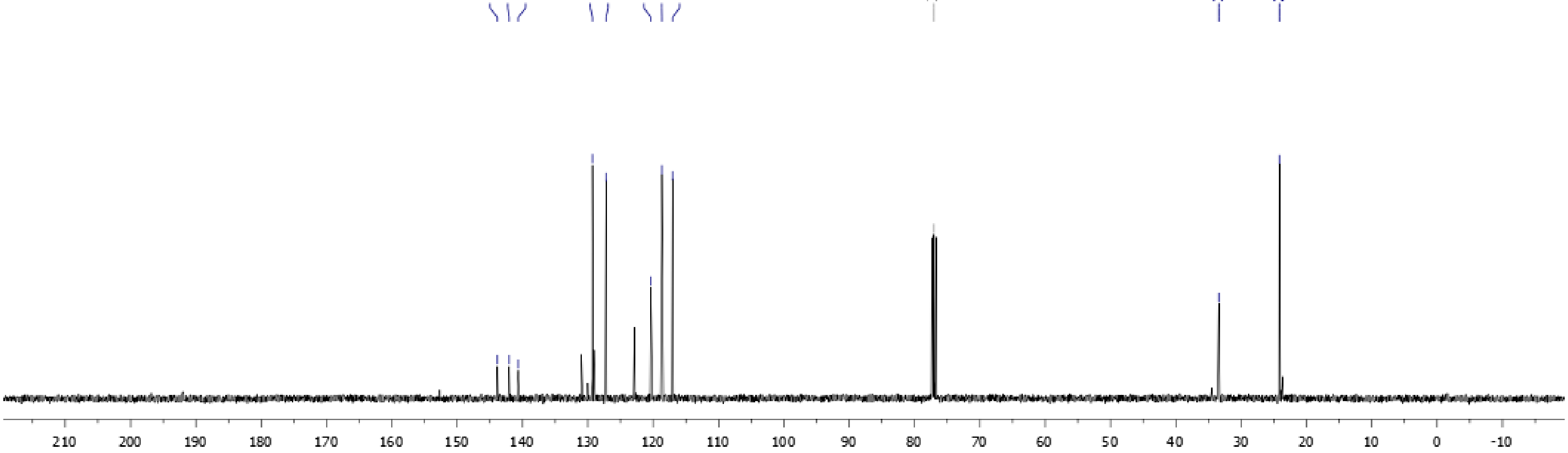

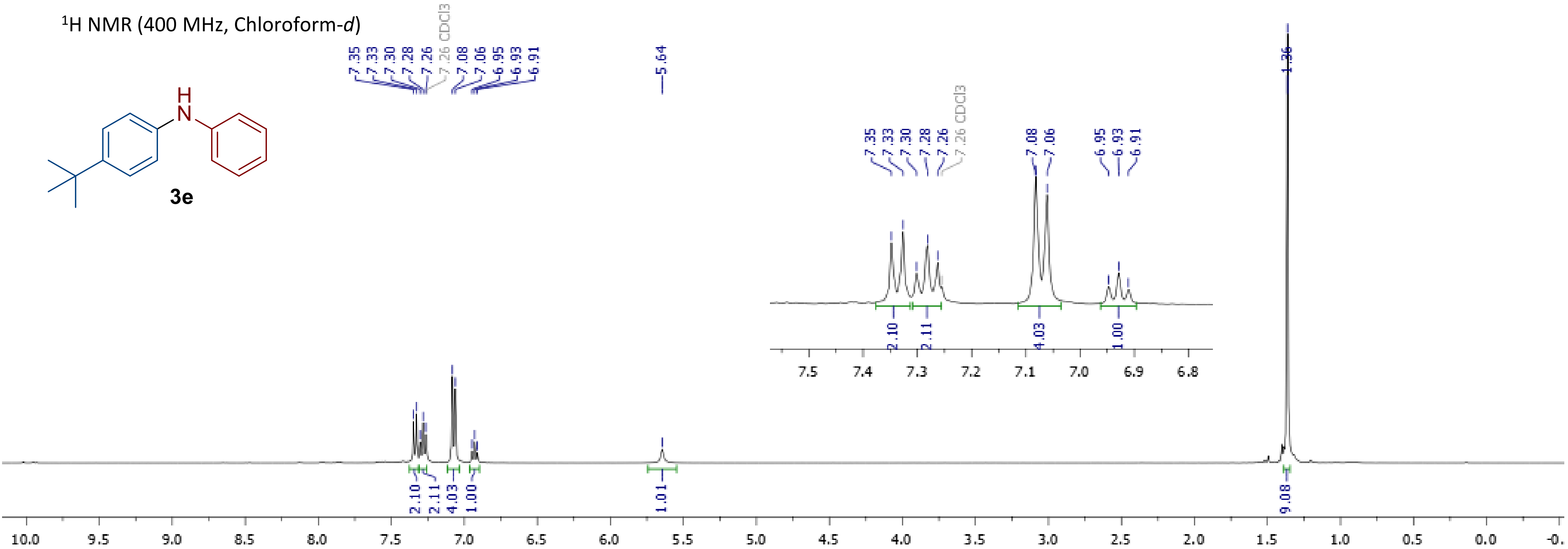

3e

1H NMR (400 MHz, Chloroform-d)
7.35
7.33
7.30
7.28
7.26
7.26 CDCl3
7.08
7.06
6.95
6.93
6.91
5.64
1.36
2.10
2.11
4.03
1.00
1.01
9.08
10.0 9.5 9.0 8.5 8.0 7.5 7.0 6.5 6.0 5.5 5.0 4.5 4.0 3.5 3.0 2.5 2.0 1.5 1.0 0.5 0.0
7.5 7.4 7.3 7.2 7.1 7.0 6.9 6.8

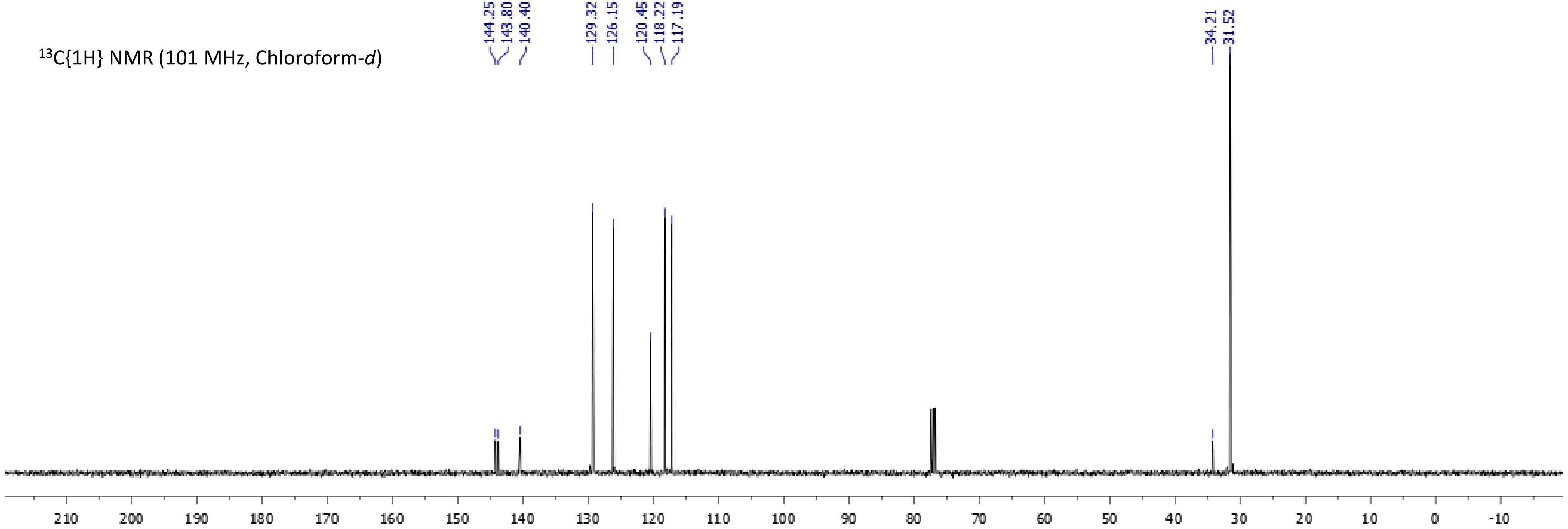

13C{1H} NMR (101 MHz, Chloroform-d)
144.25
143.80
140.40
129.32
126.15
120.45
118.22
117.19
34.21
31.52
210 200 190 180 170 160 150 140 130 120 110 100 90 80 70 60 50 40 30 20 10 0 -10

^{1}H NMR (400 MHz, Chloroform-*d*)

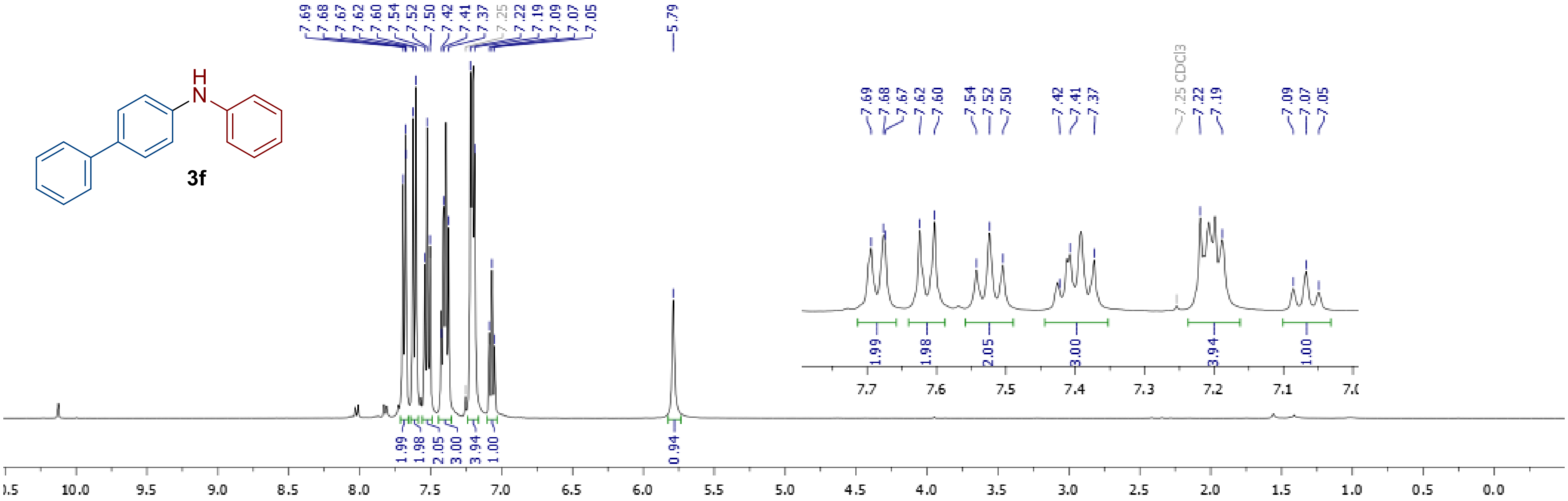

^{13}C{1H} NMR (101 MHz, Chloroform-*d*)

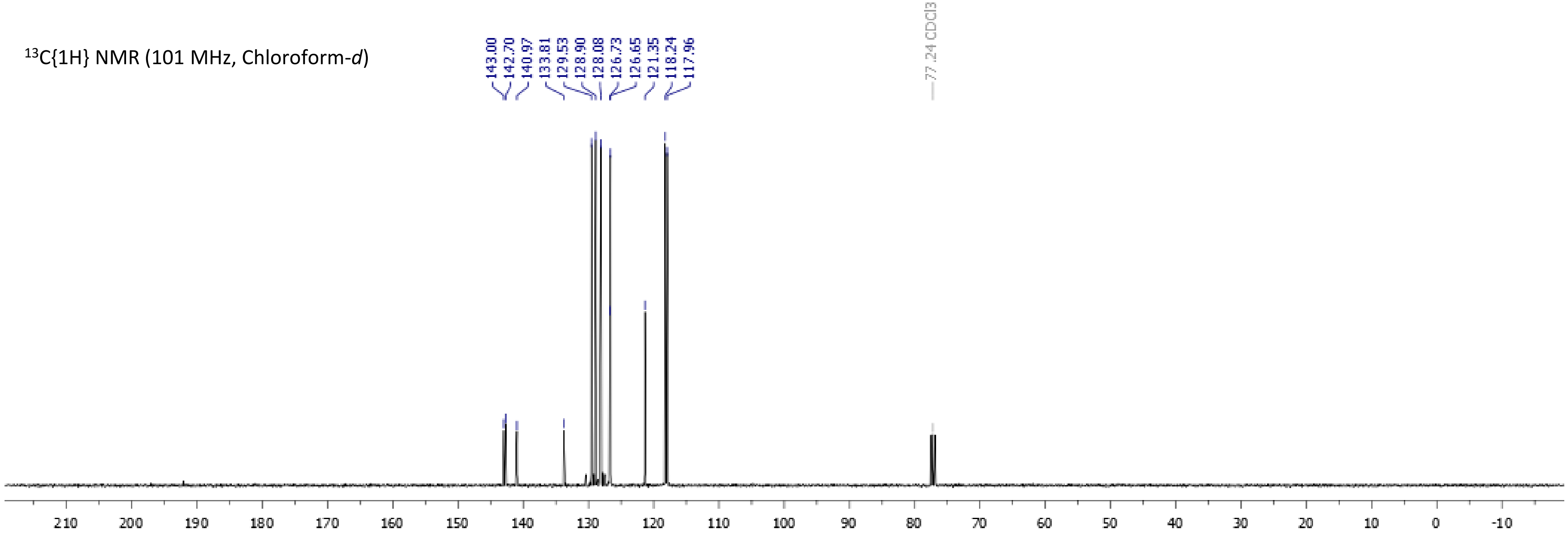

^{1}H NMR (400 MHz, Chloroform-*d*)

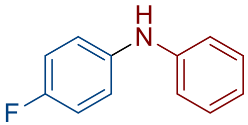

3g

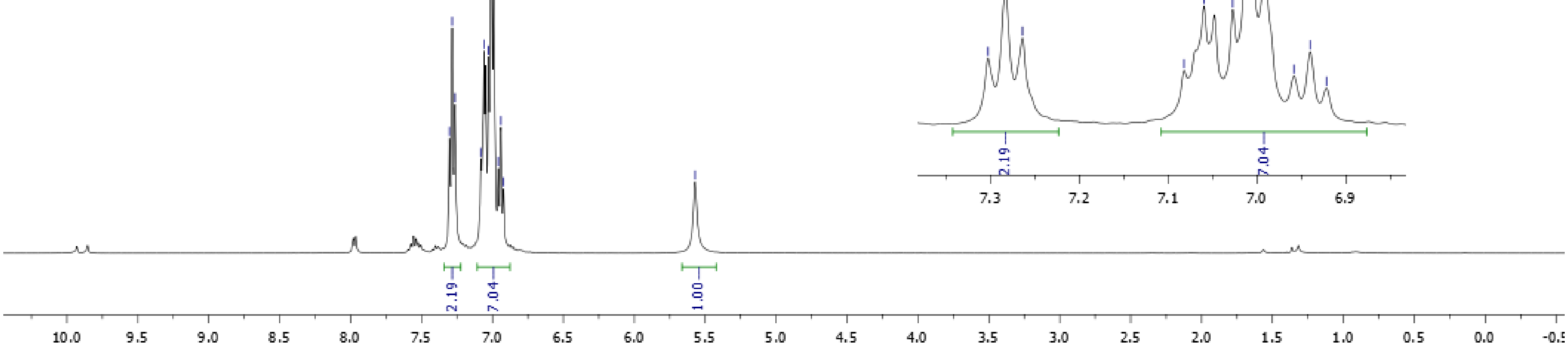

^{13}C{1H} NMR (101 MHz, Chloroform-*d*)

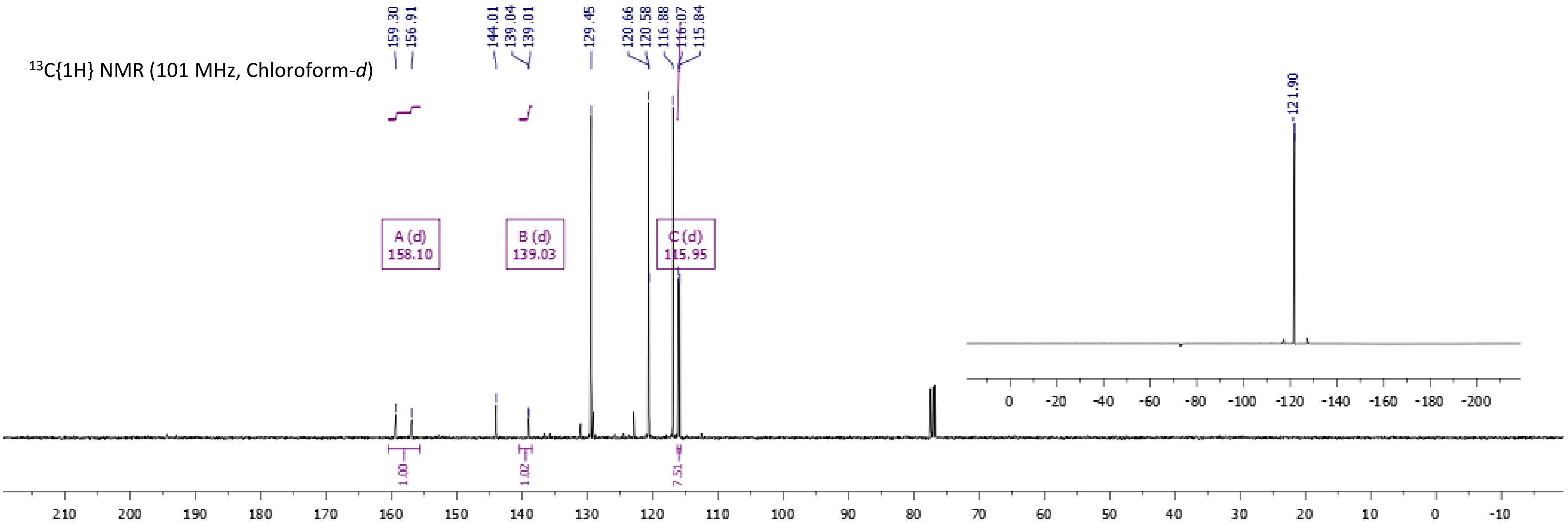

^{1}H NMR (400 MHz, Chloroform-*d*)

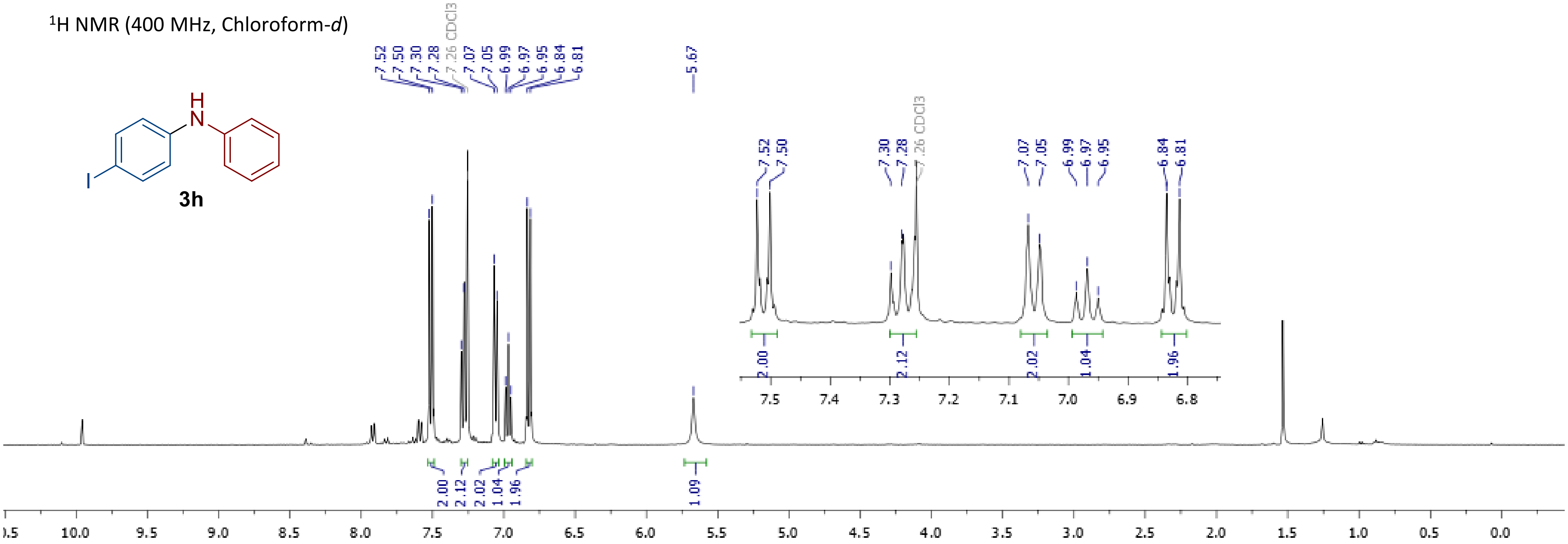

^{13}C{1H} NMR (101 MHz, Chloroform-*d*)

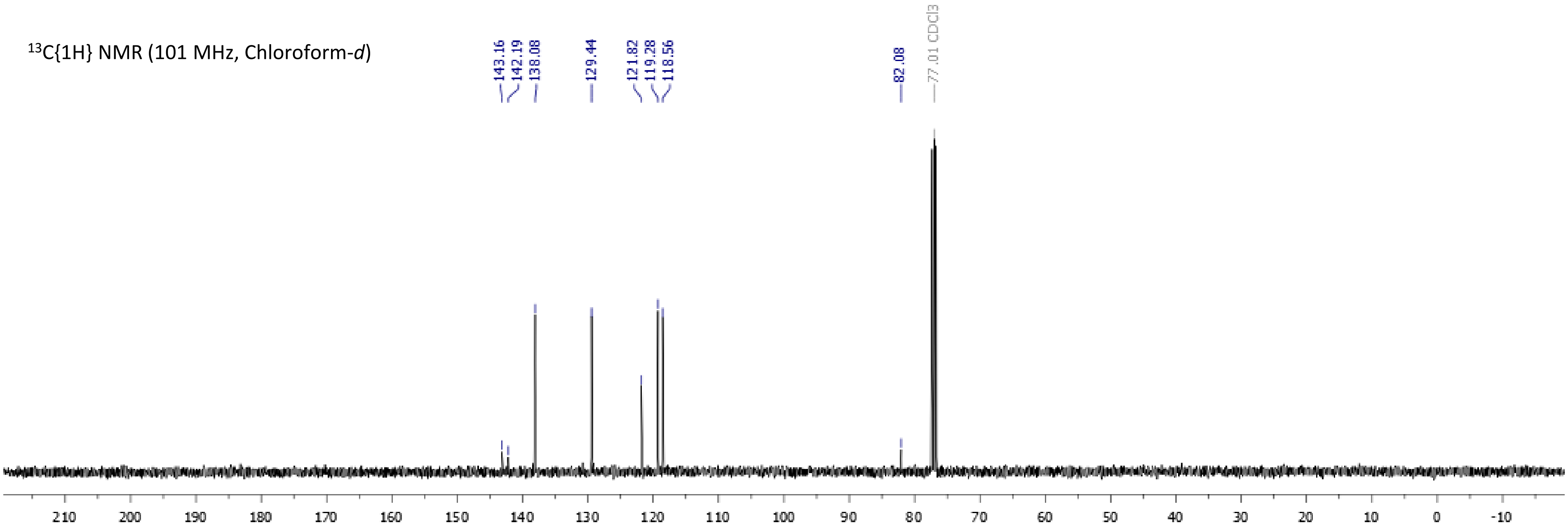

^{1}H NMR (400 MHz, Chloroform-*d*)

3i

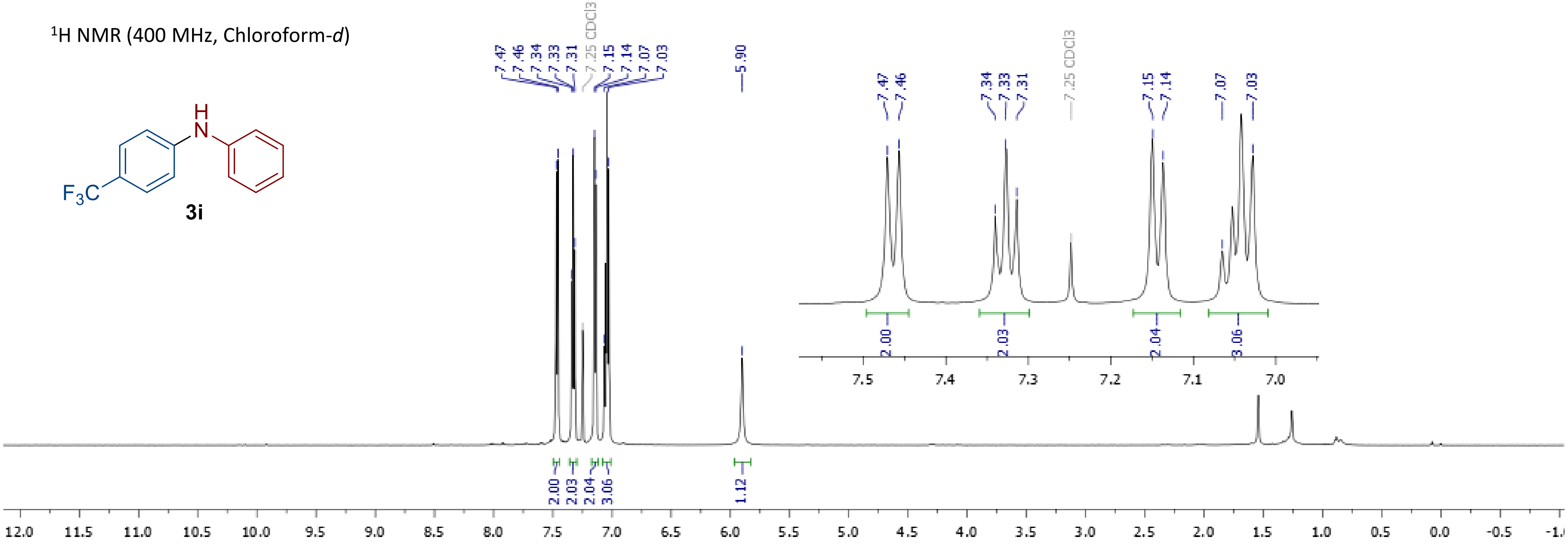

^{13}C{1H} NMR (101 MHz, Chloroform-*d*)

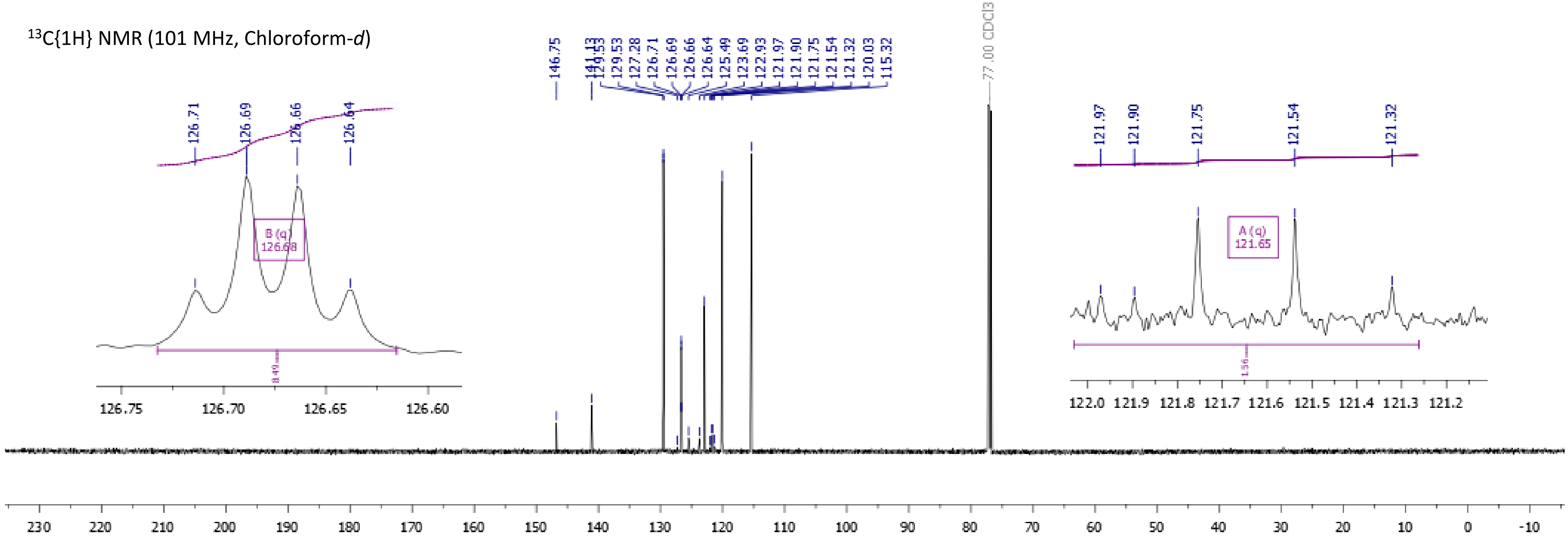

^{19}F NMR (376 MHz, Chloroform-*d*)

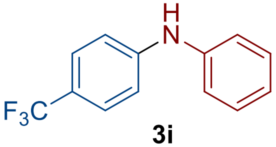

3i

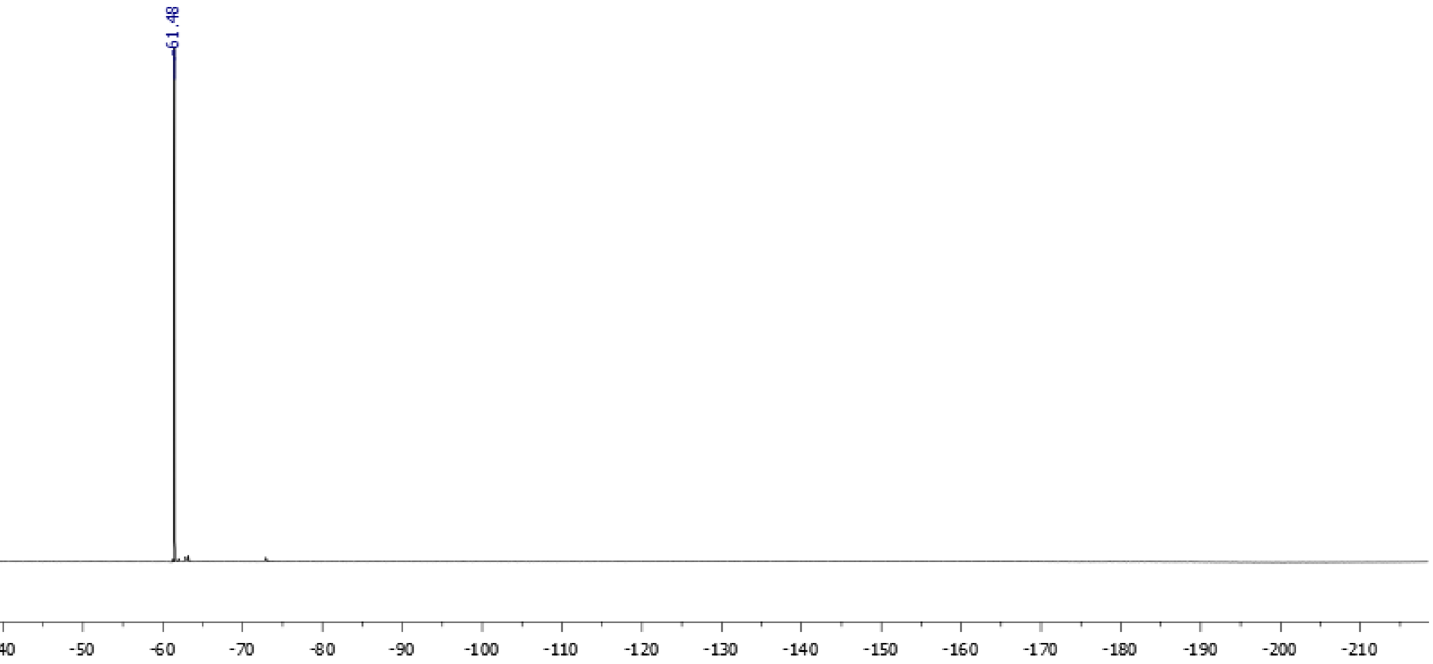

^{13}C{1H} NMR (101 MHz, Chloroform-*d*)

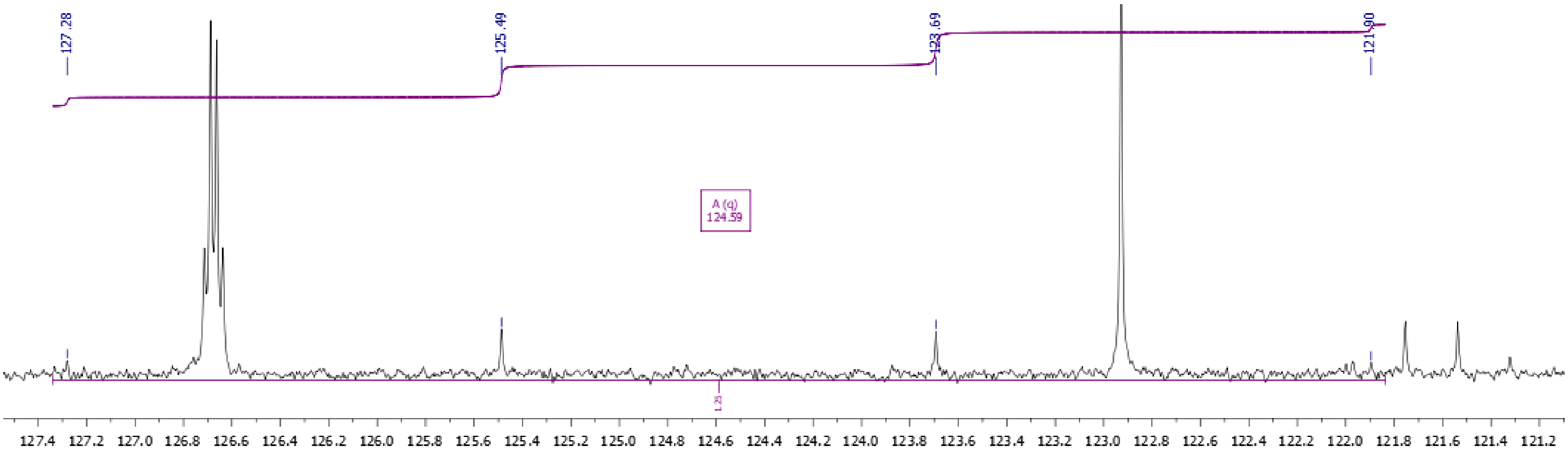

^{1}H NMR (400 MHz, Chloroform-*d*)

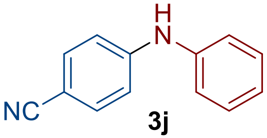

3j

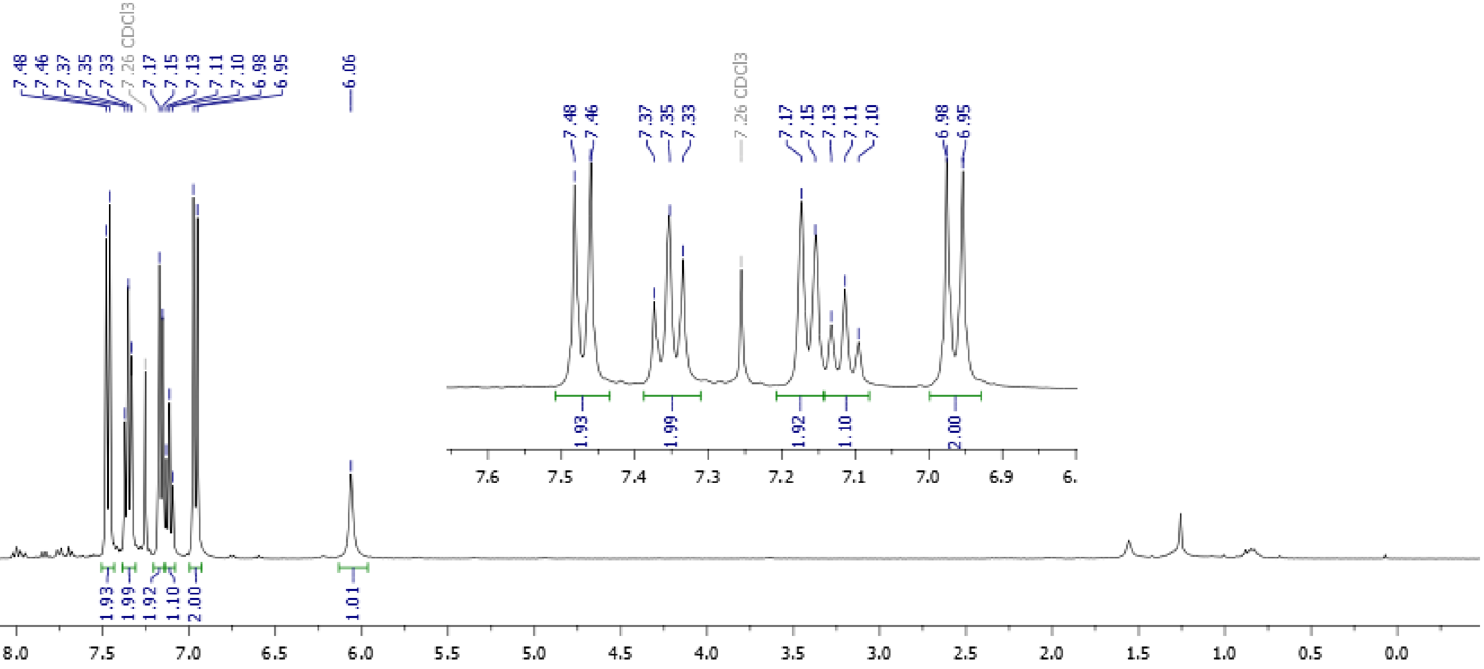

$^{13}C\{1H\}$ NMR (101 MHz, Chloroform-*d*)

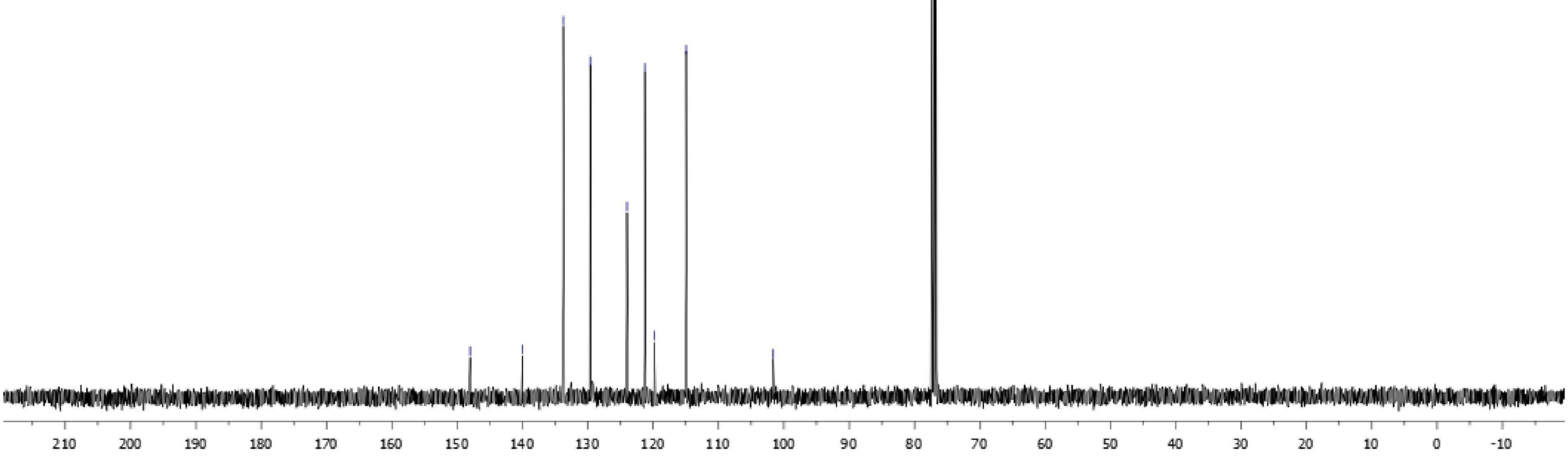

^{1}H NMR (400 MHz, Chloroform-*d*)

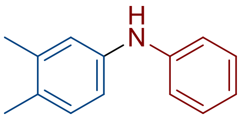

3k

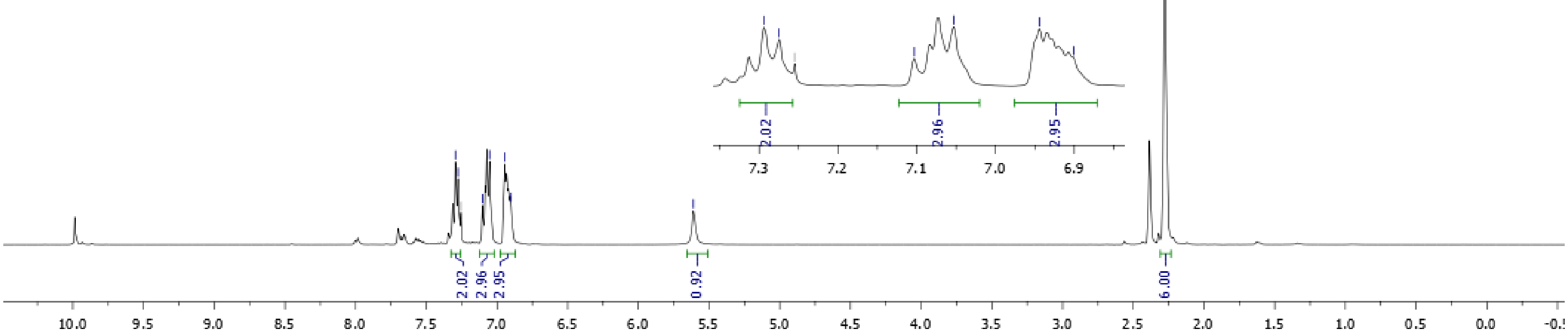

$^{13}C\{^{1}H\}$ NMR (101 MHz, Chloroform-*d*)

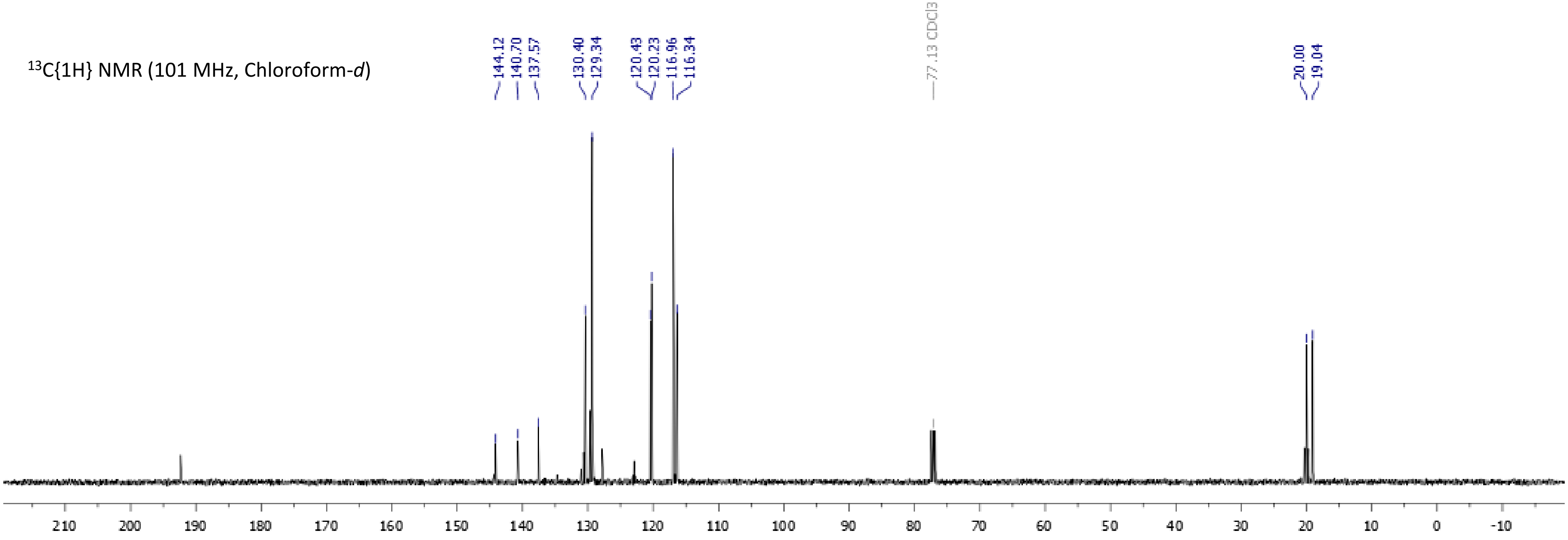

^{1}H NMR (400 MHz, Chloroform-*d*)

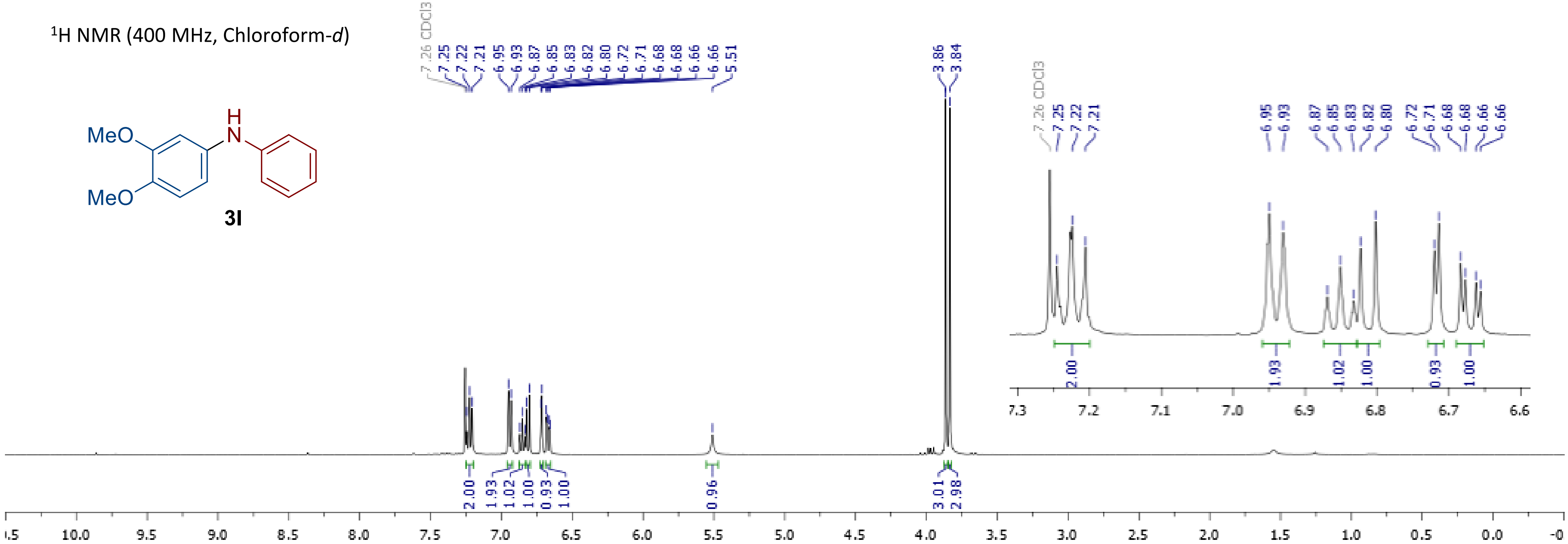

$^{13}C\{^1H\}$ NMR (101 MHz, Chloroform-*d*)

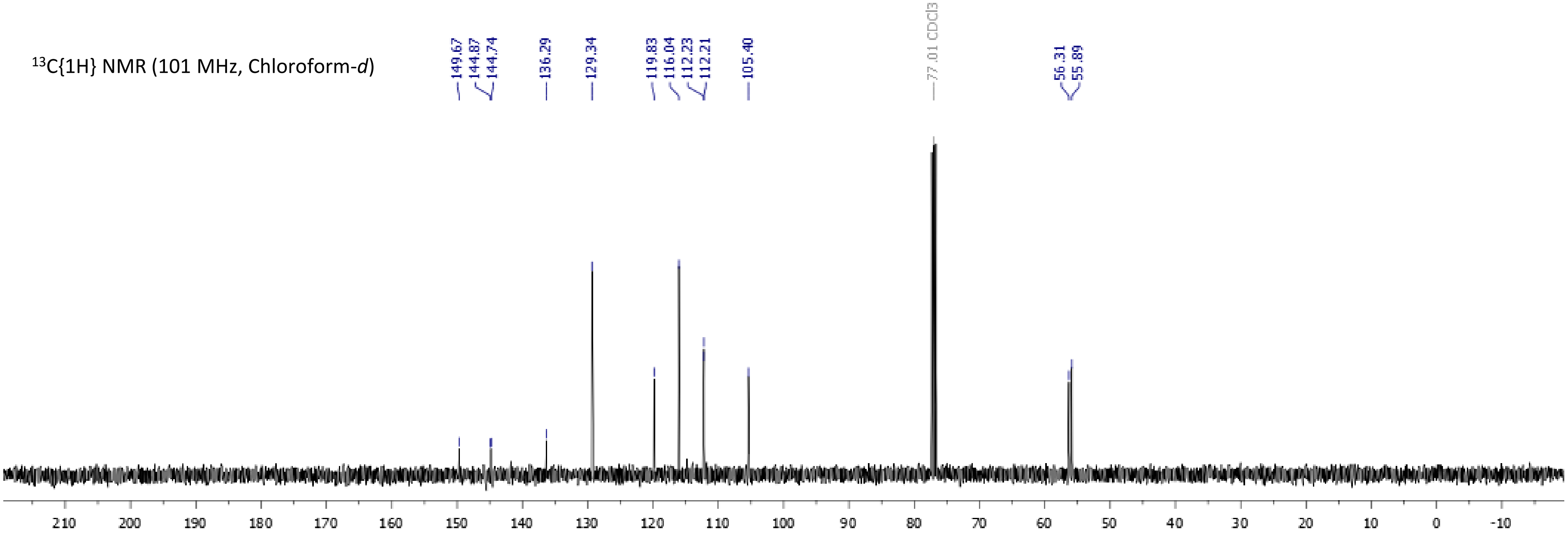

^{1}H NMR (400 MHz, Chloroform-*d*)

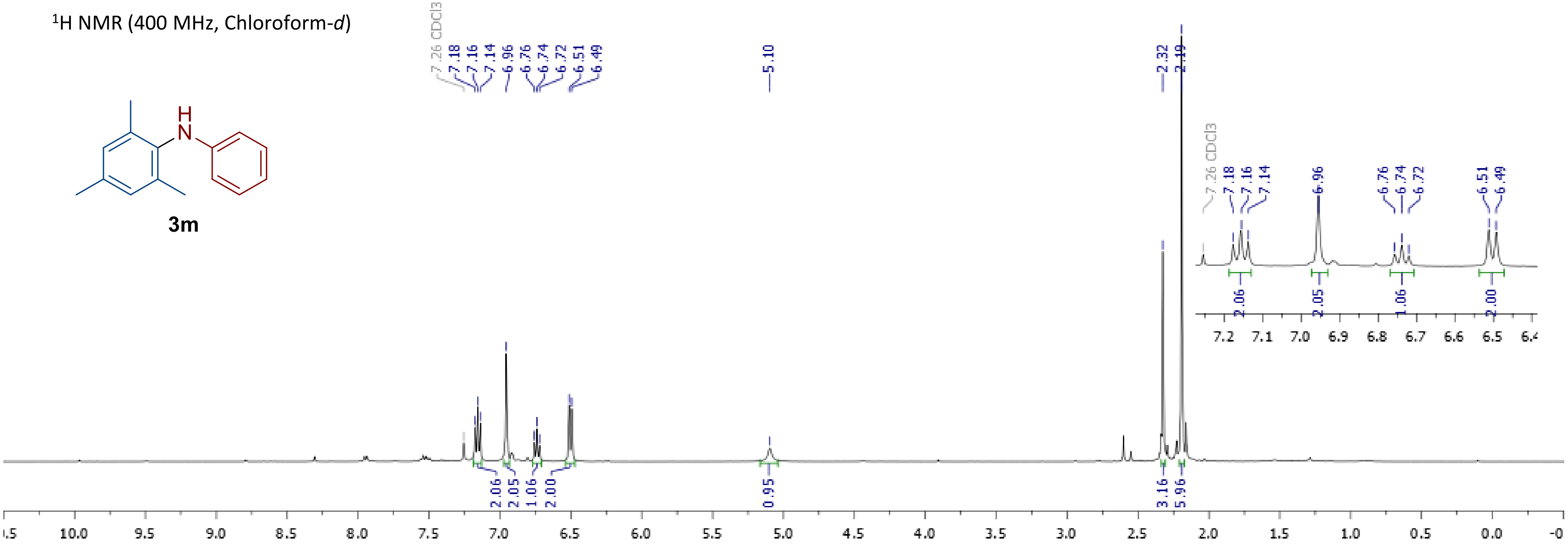

^{13}C{1H} NMR (101 MHz, Chloroform-*d*)

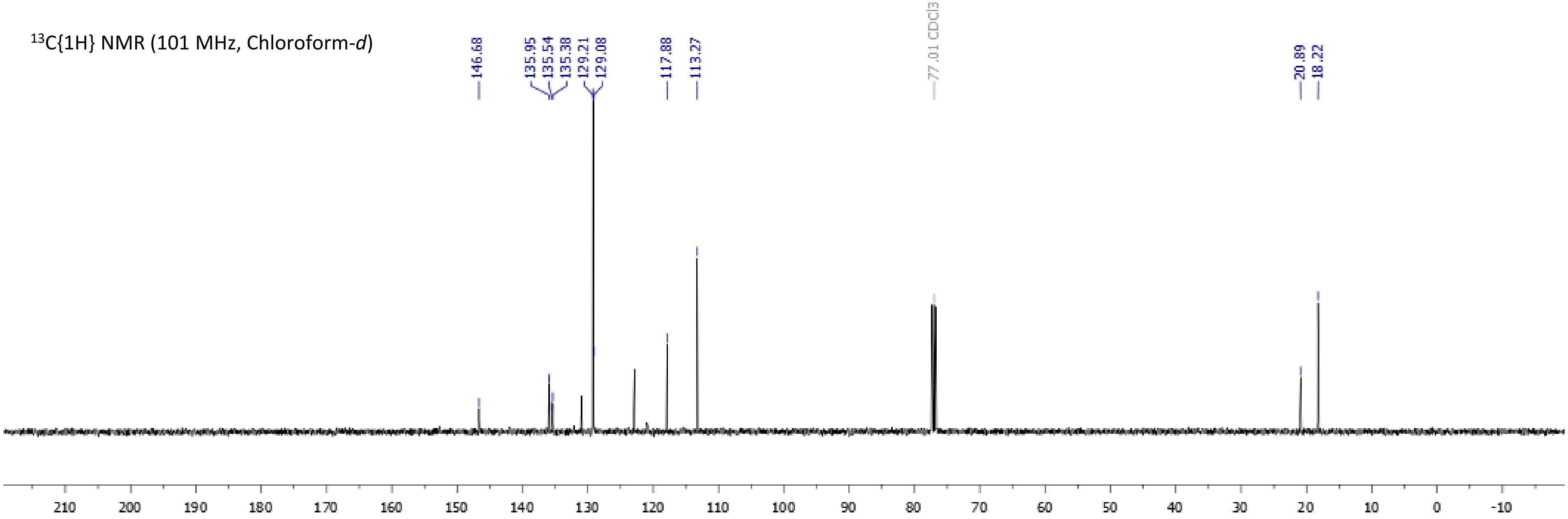

^{1}H NMR (400 MHz, Chloroform-*d*)

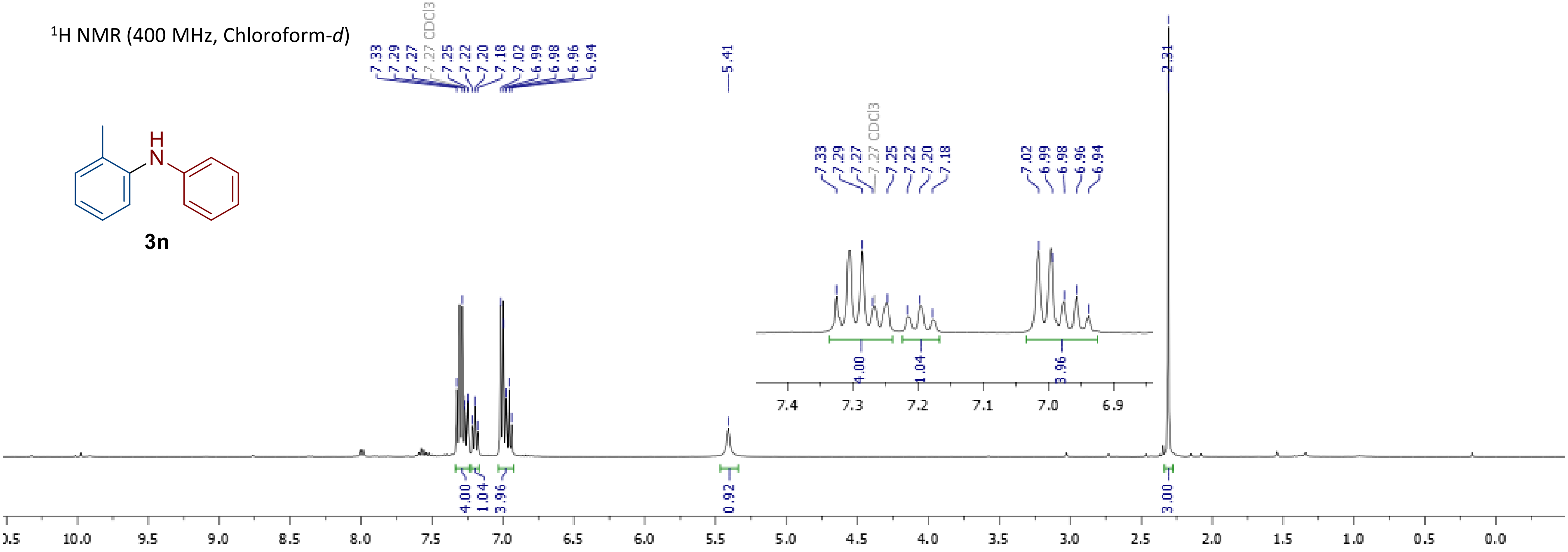

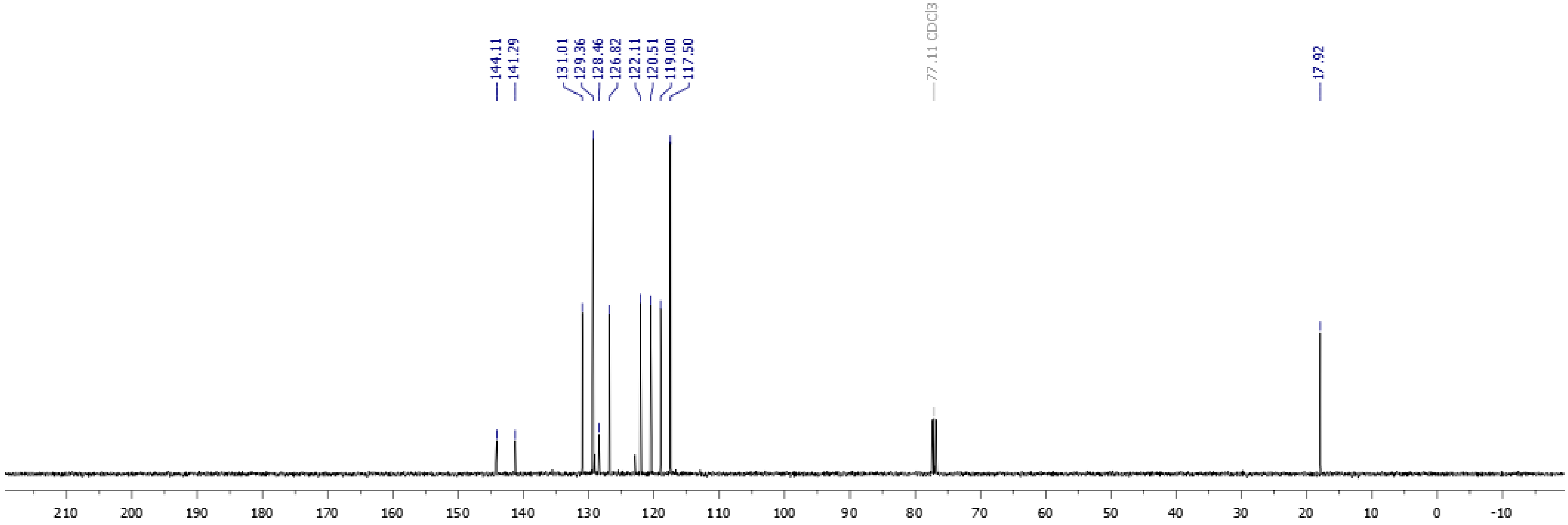

^{1}H NMR (400 MHz, Chloroform-*d*)

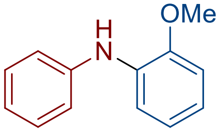

3o

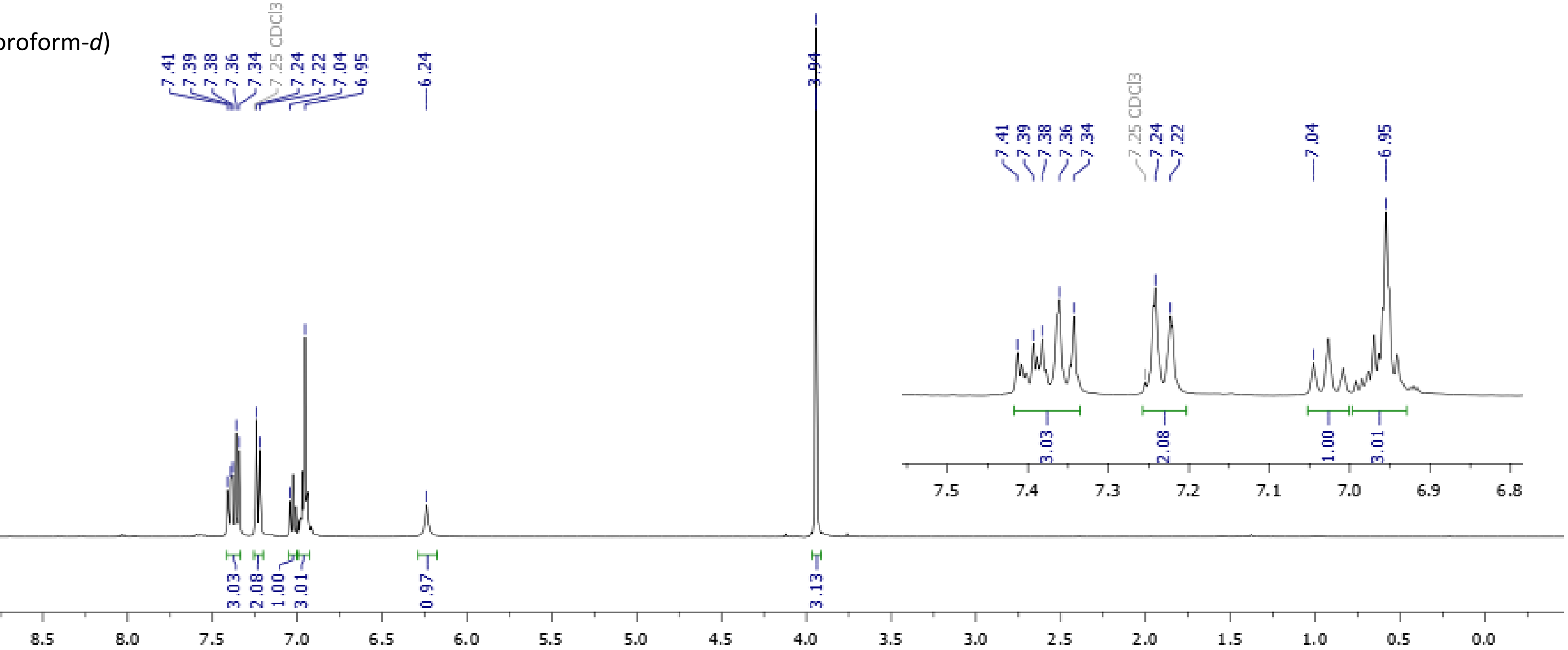

$^{13}C\{^{1}H\}$ NMR (101 MHz, Chloroform-*d*)

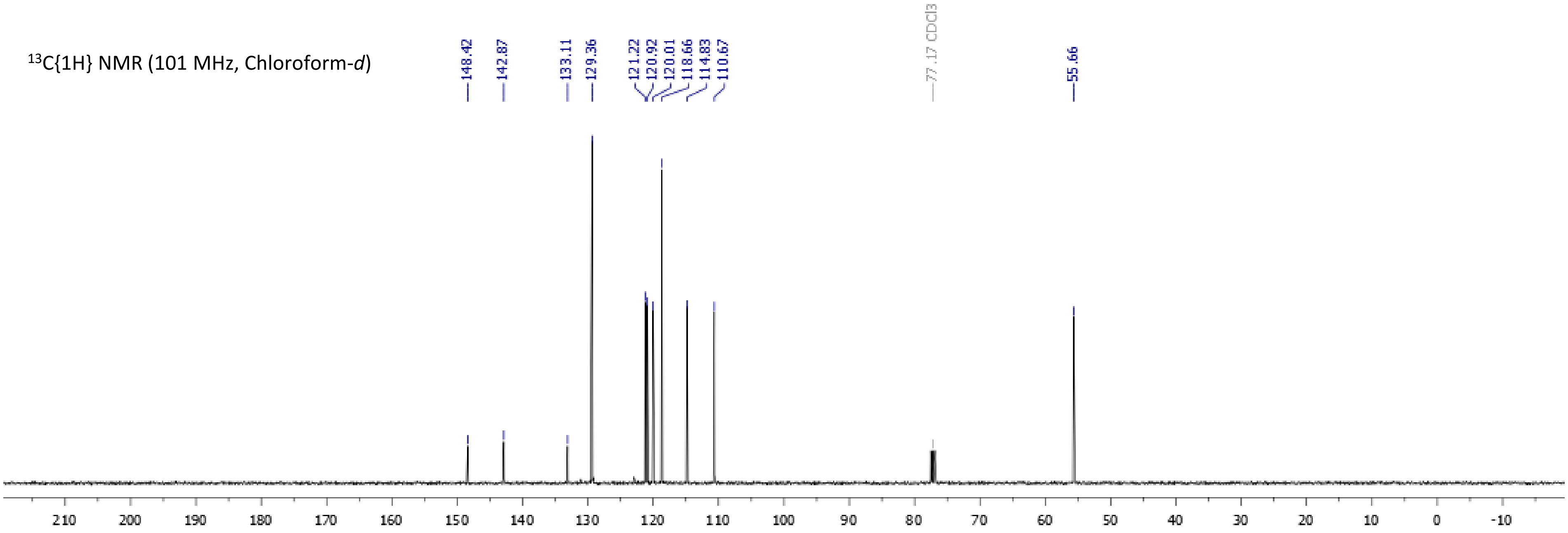

^{1}H NMR (400 MHz, Chloroform-*d*)

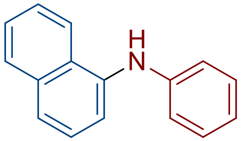

3p

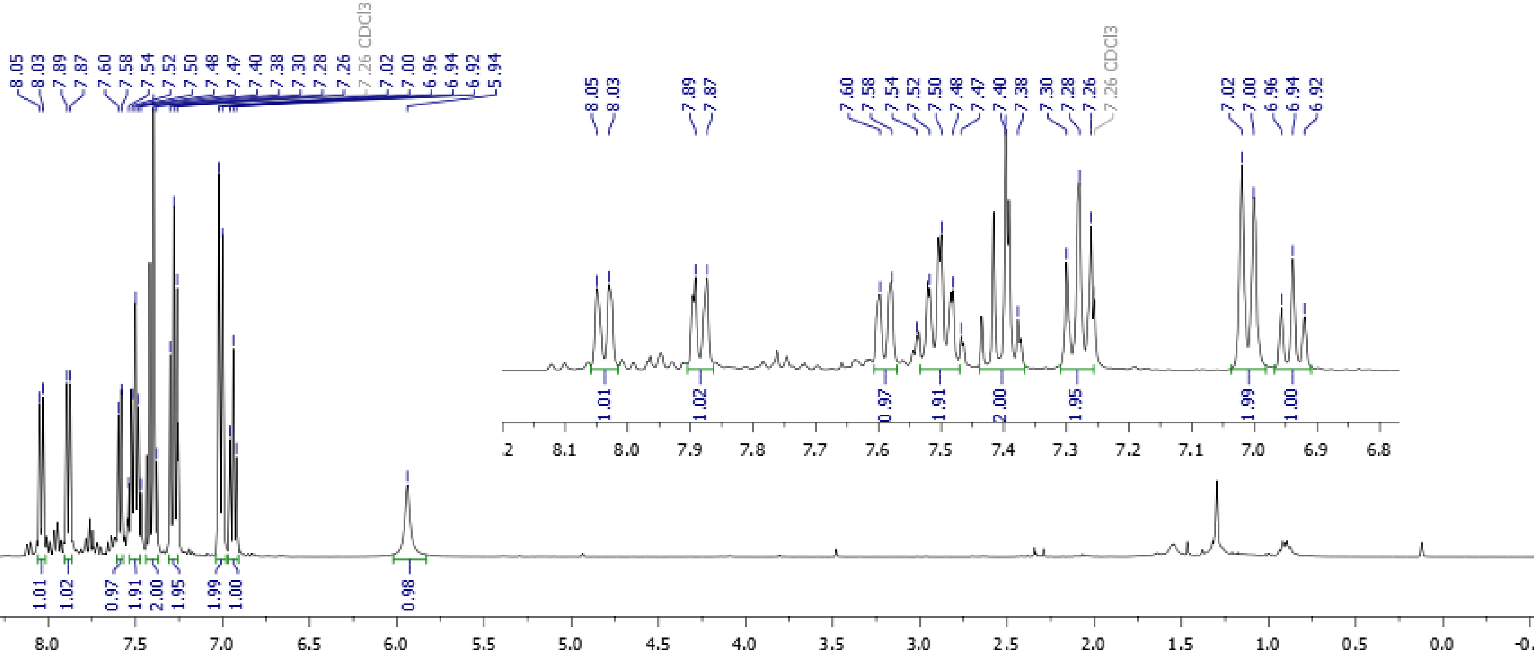

^{13}C{1H} NMR (101 MHz, Chloroform-*d*)

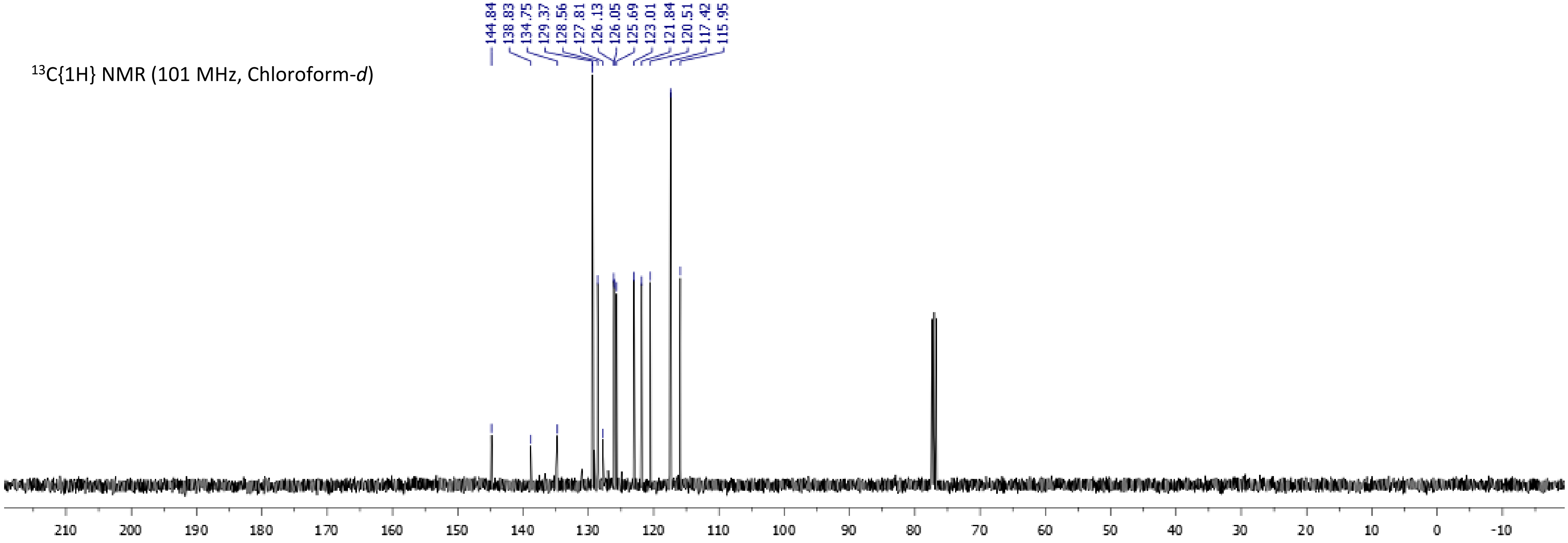

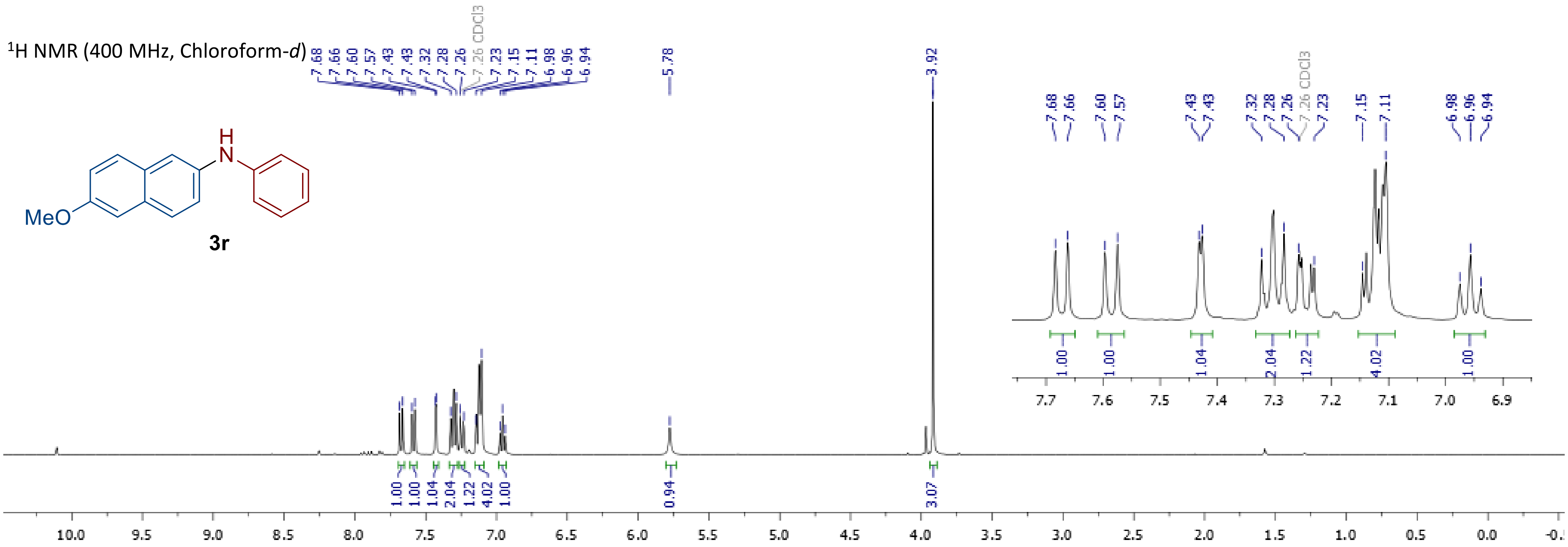

3r
1H NMR (400 MHz, Chloroform-d)
7.68 7.66 7.60 7.57 7.43 7.43 7.32 7.28 7.26 7.26 CDCl3 7.23 7.15 7.11 6.98 6.96 6.94
5.78
3.92
1.00 1.00 1.04 2.04 1.22 4.02 1.00
0.94
3.07

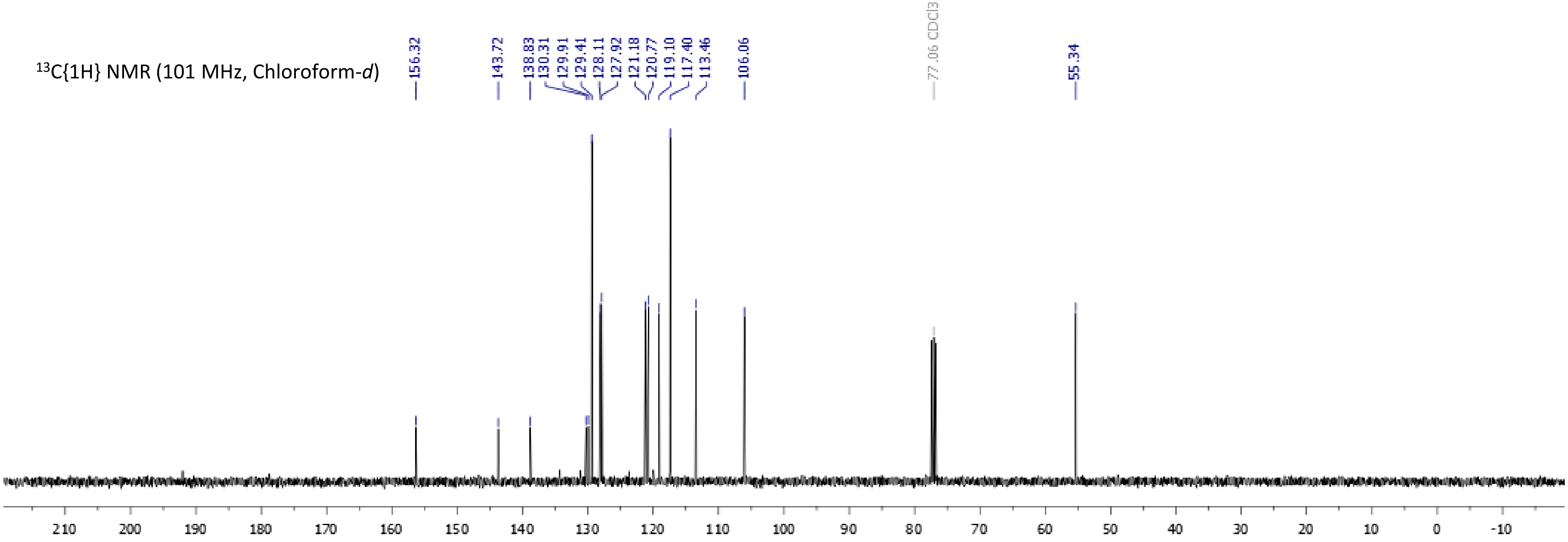

13C{1H} NMR (101 MHz, Chloroform-d)
156.32
143.72
138.83 130.31 129.91 129.41 128.11 127.92
121.18 120.77 119.10 117.40 113.46
106.06
77.06 CDCl3
55.34

^{1}H NMR (400 MHz, Chloroform-*d*)

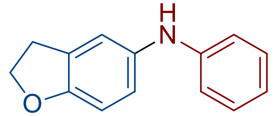

3s

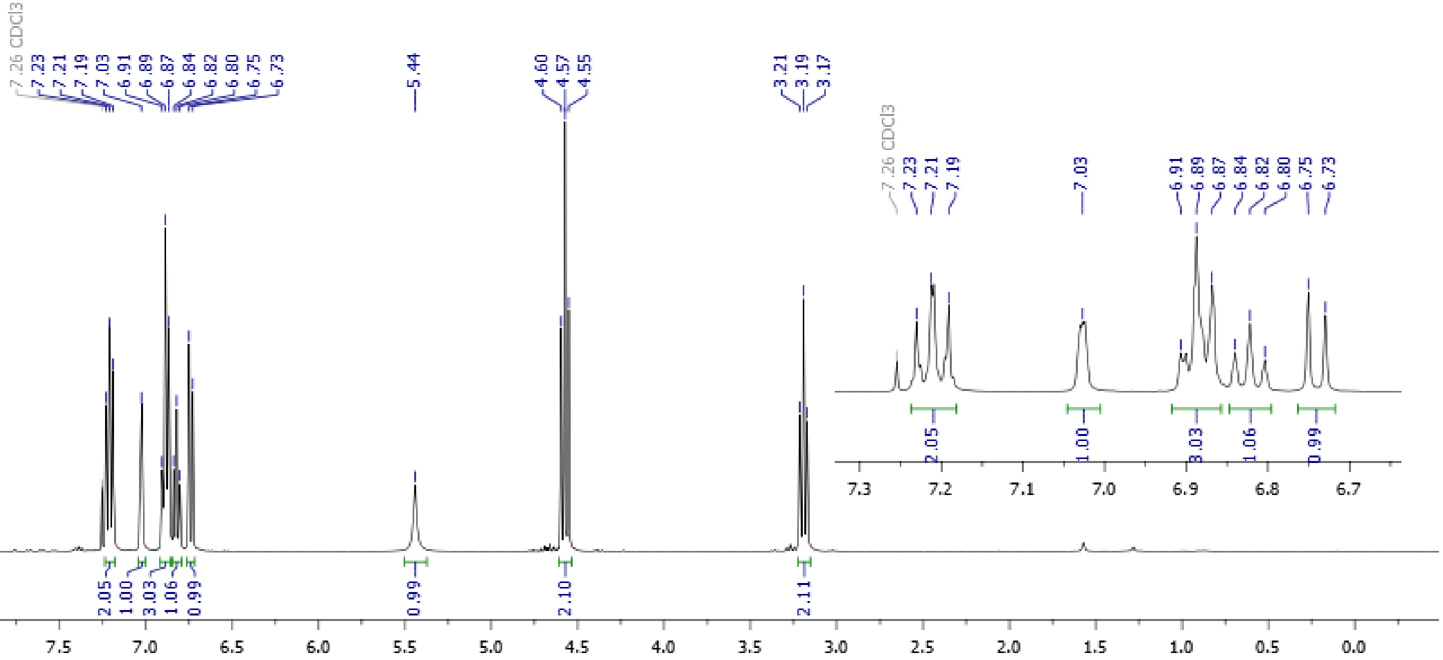

^{13}C{1H} NMR (101 MHz, Chloroform-*d*)

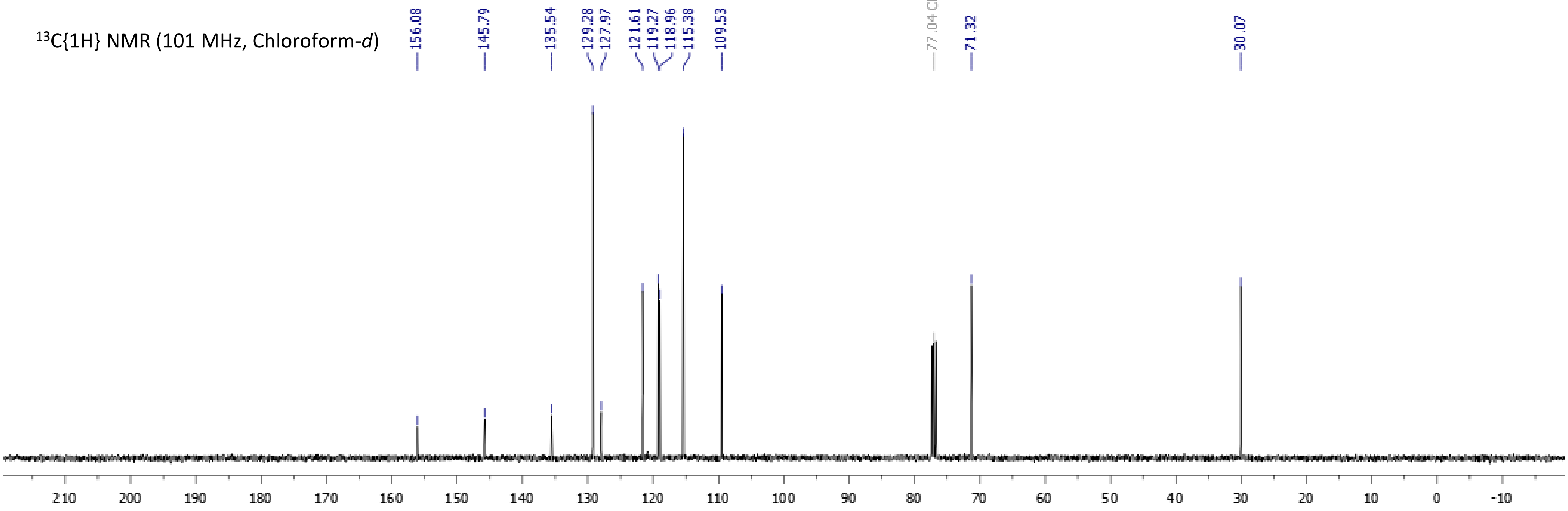

^{1}H NMR (400 MHz, Chloroform-*d*)

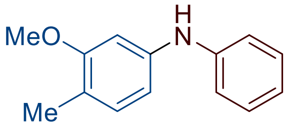

3t

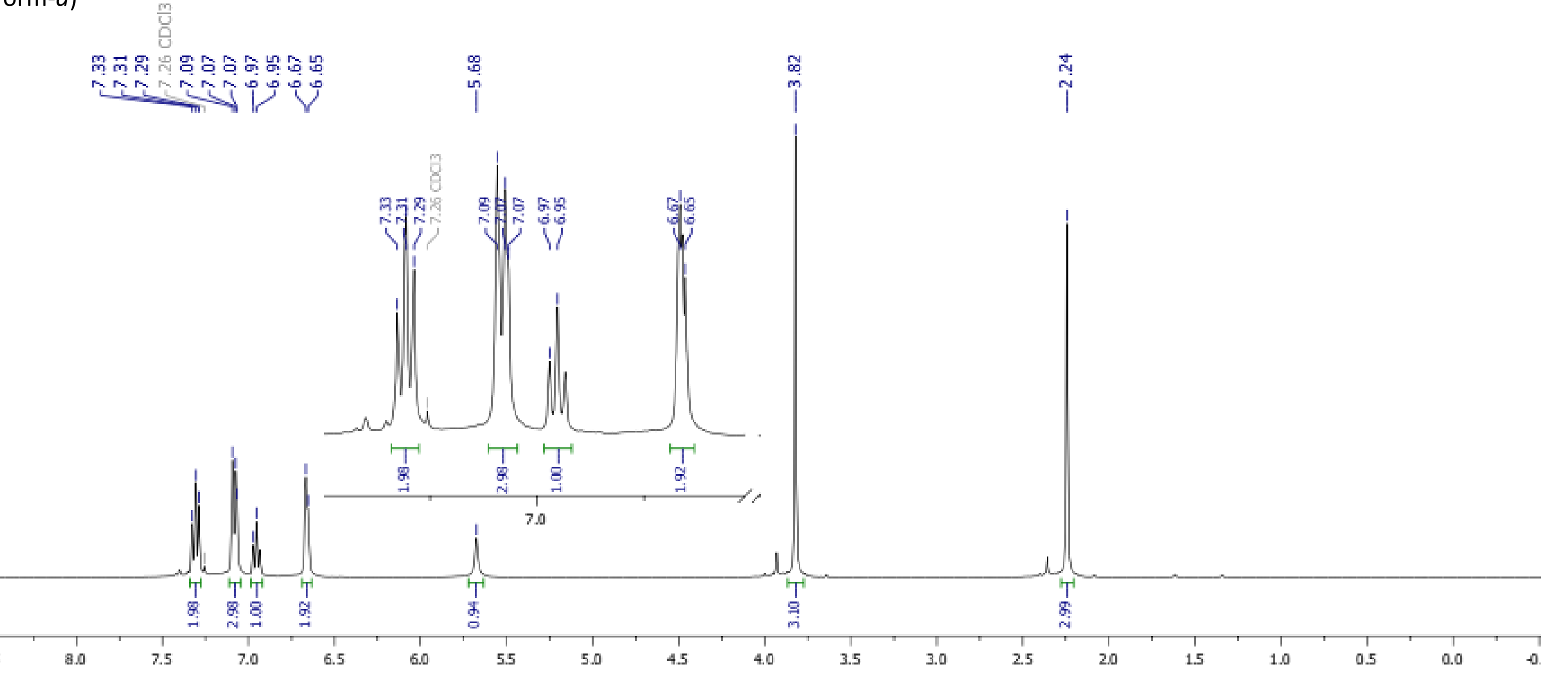

$^{13}C\{1H\}$ NMR (101 MHz, Chloroform-*d*)

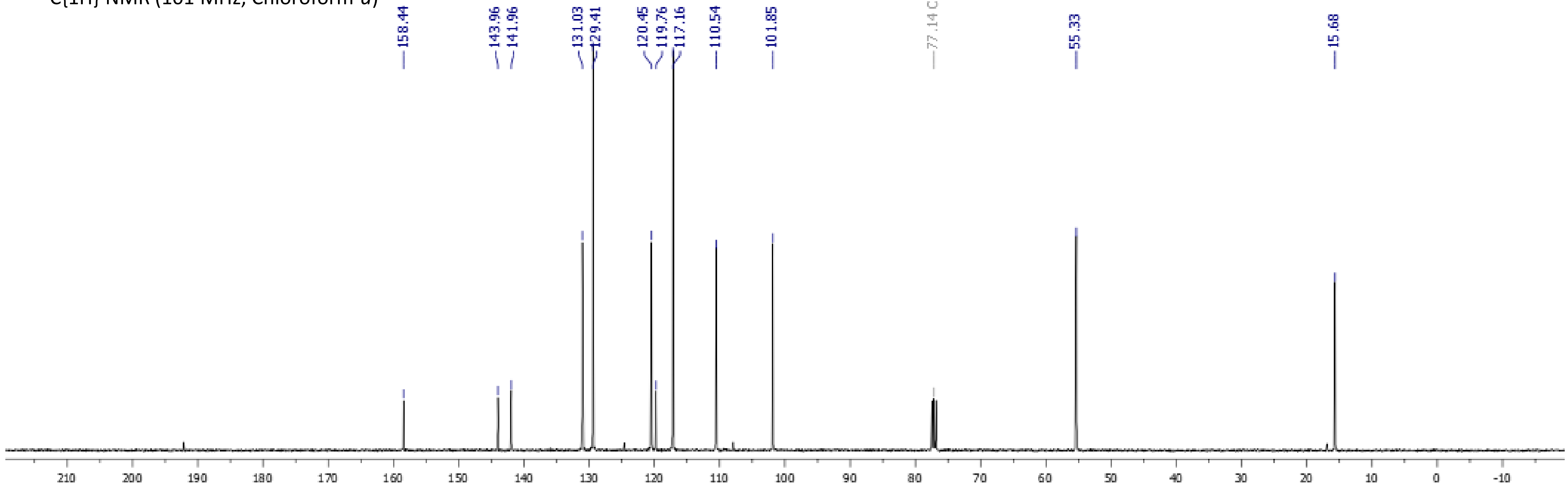

^{1}H NMR (400 MHz, Chloroform-*d*)

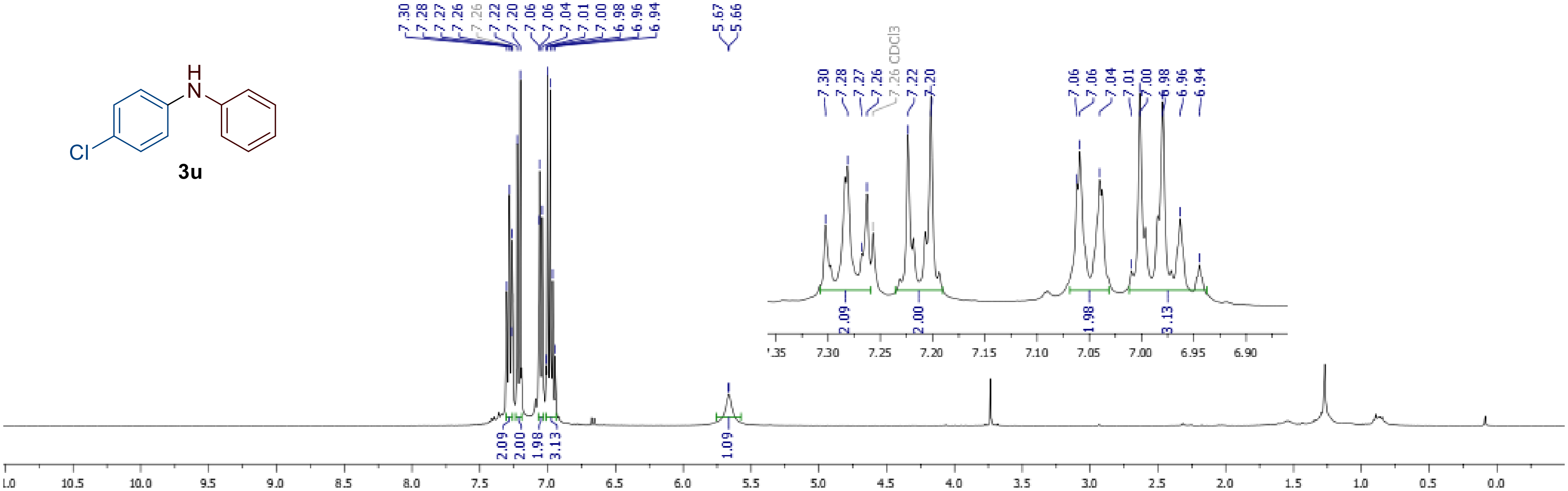

3u

^{13}C NMR (101 MHz, Chloroform-*d*)

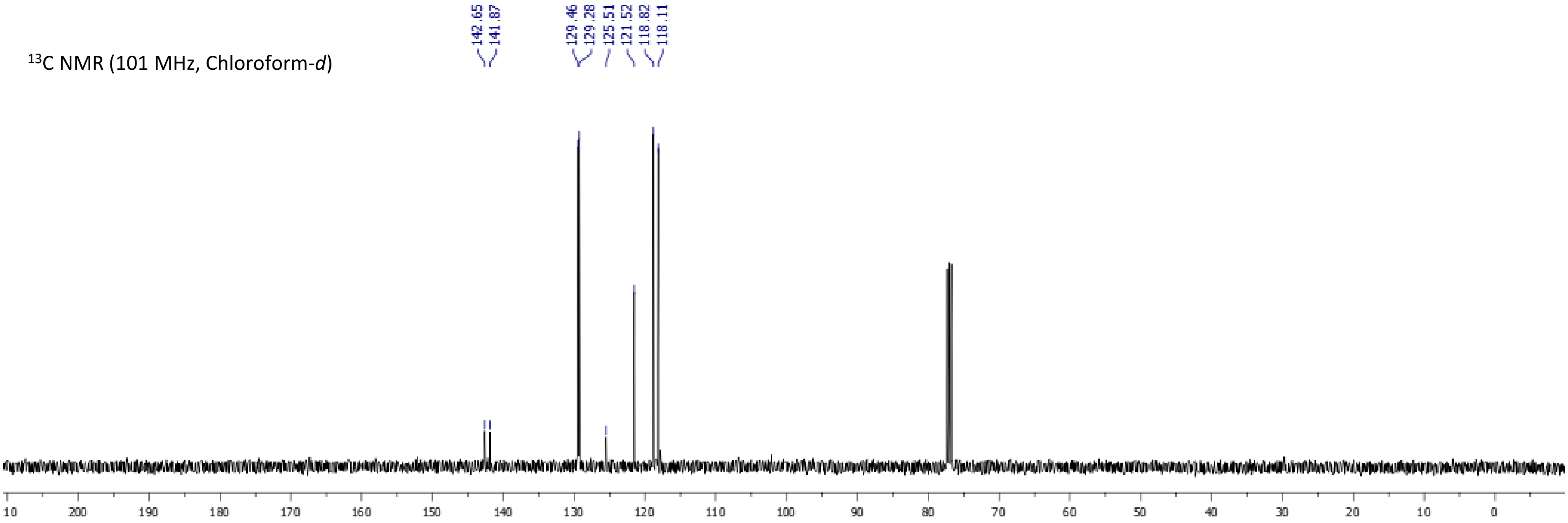

^{1}H NMR (400 MHz, Chloroform-*d*)

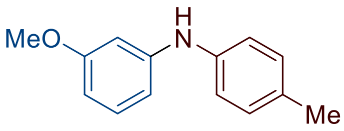

3w

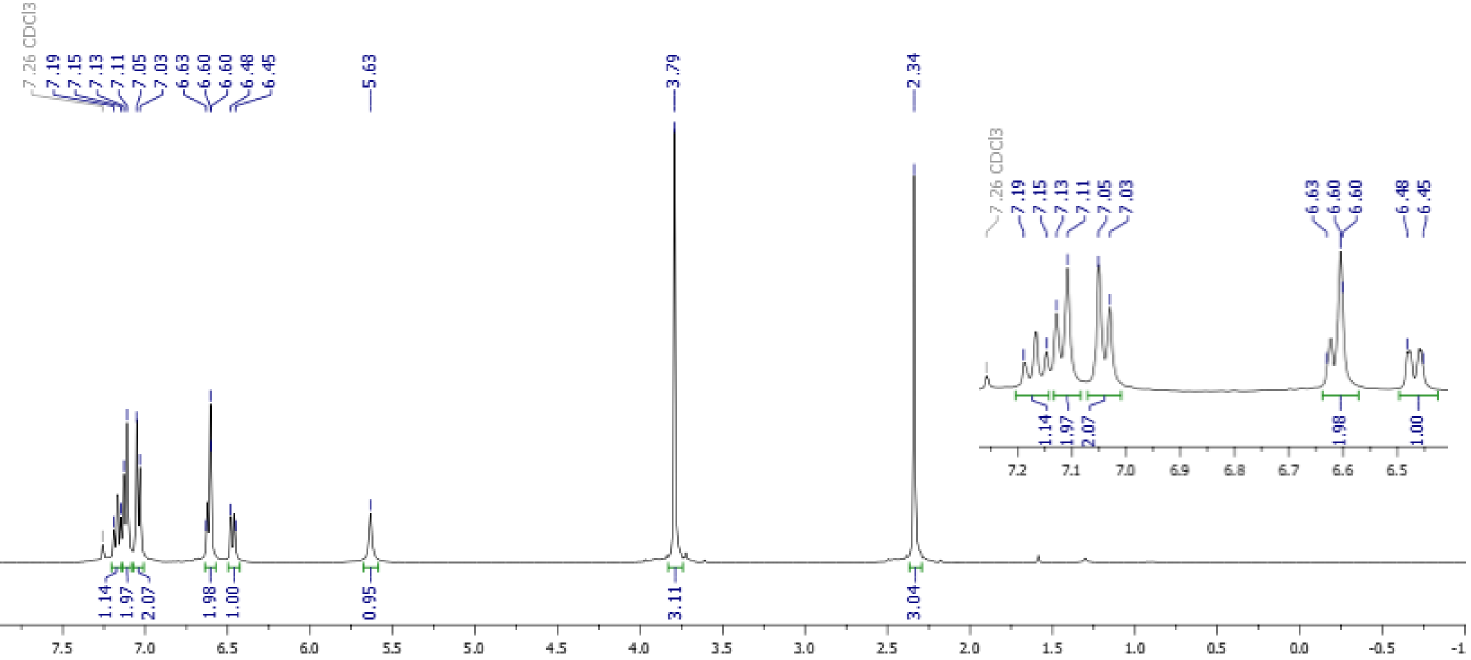

^{13}C{1H} NMR (101 MHz, Chloroform-*d*)

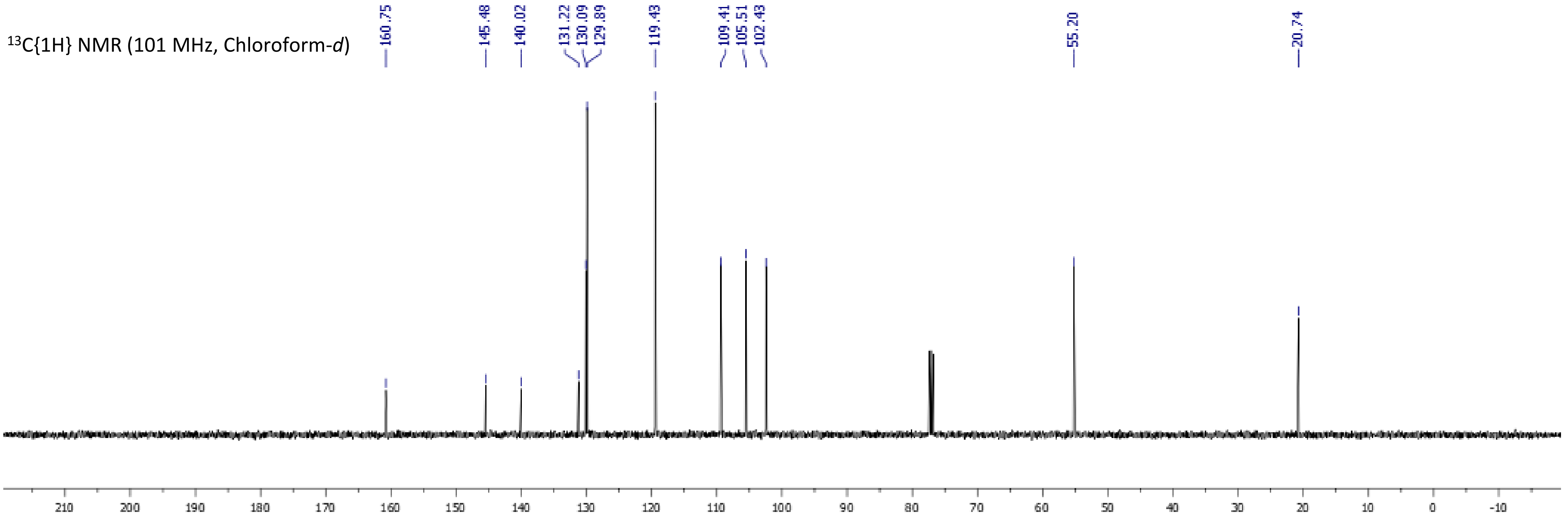

^{1}H NMR (400 MHz, Chloroform-*d*)

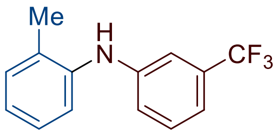

3x

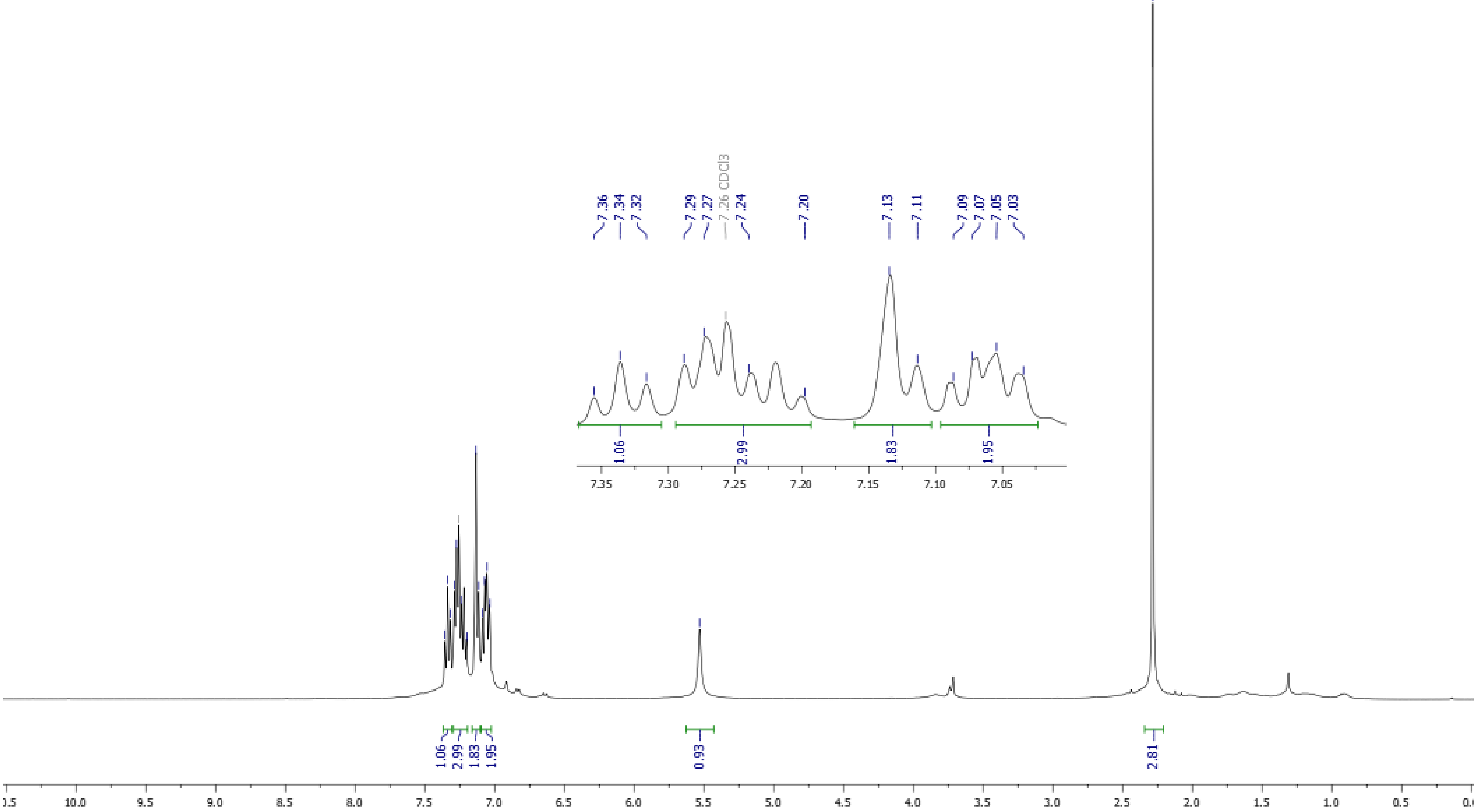

$^{13}C\{^1H\}$ NMR (101 MHz, Chloroform-*d*)

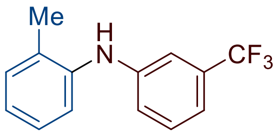

3x

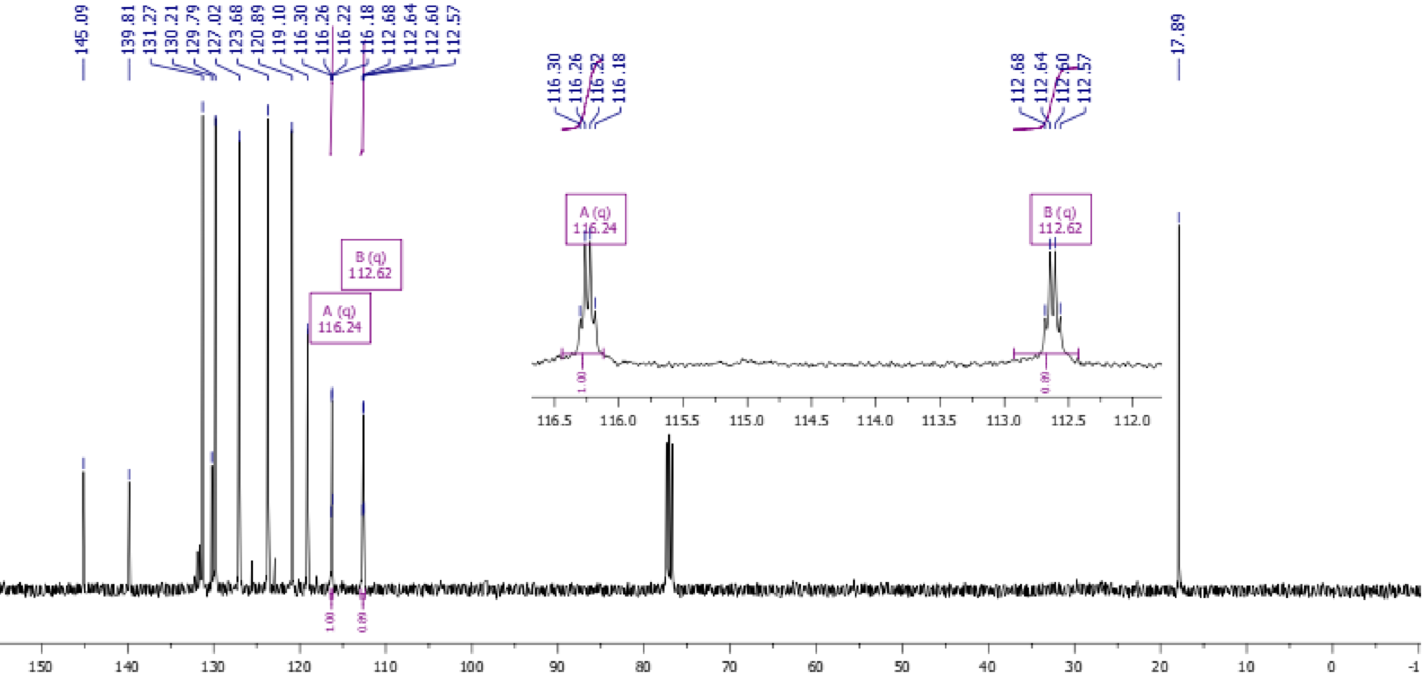

^{19}F NMR (376 MHz, Chloroform-*d*)

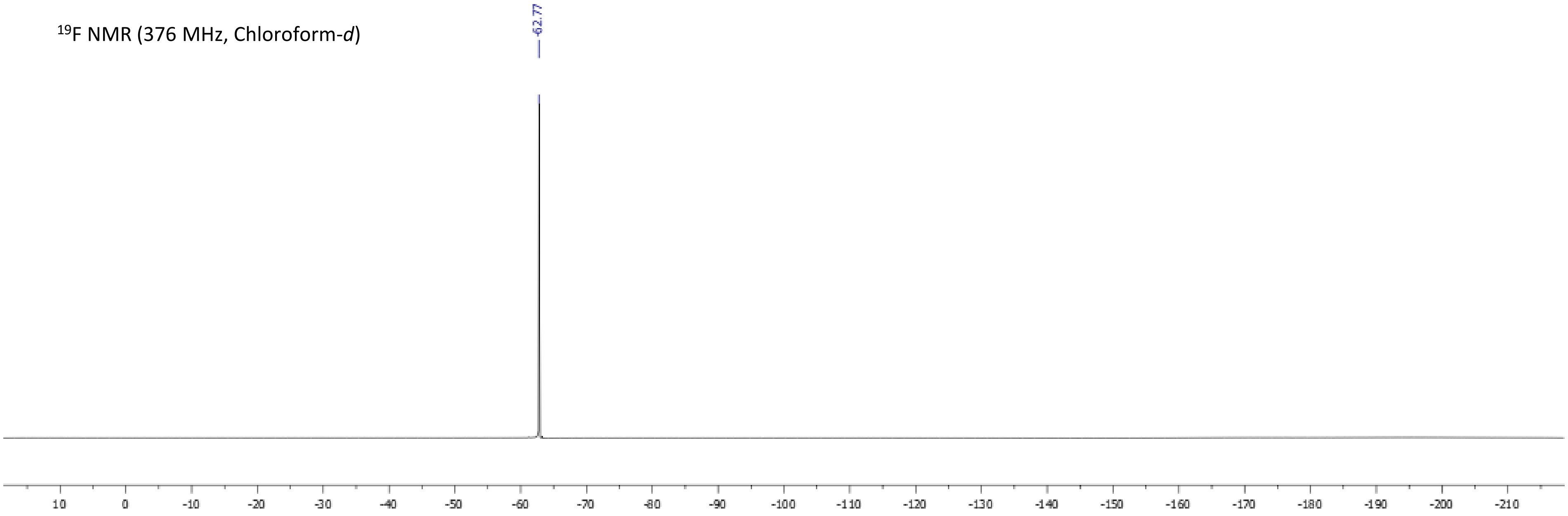

^{1}H NMR (400 MHz, Chloroform-*d*)

3y

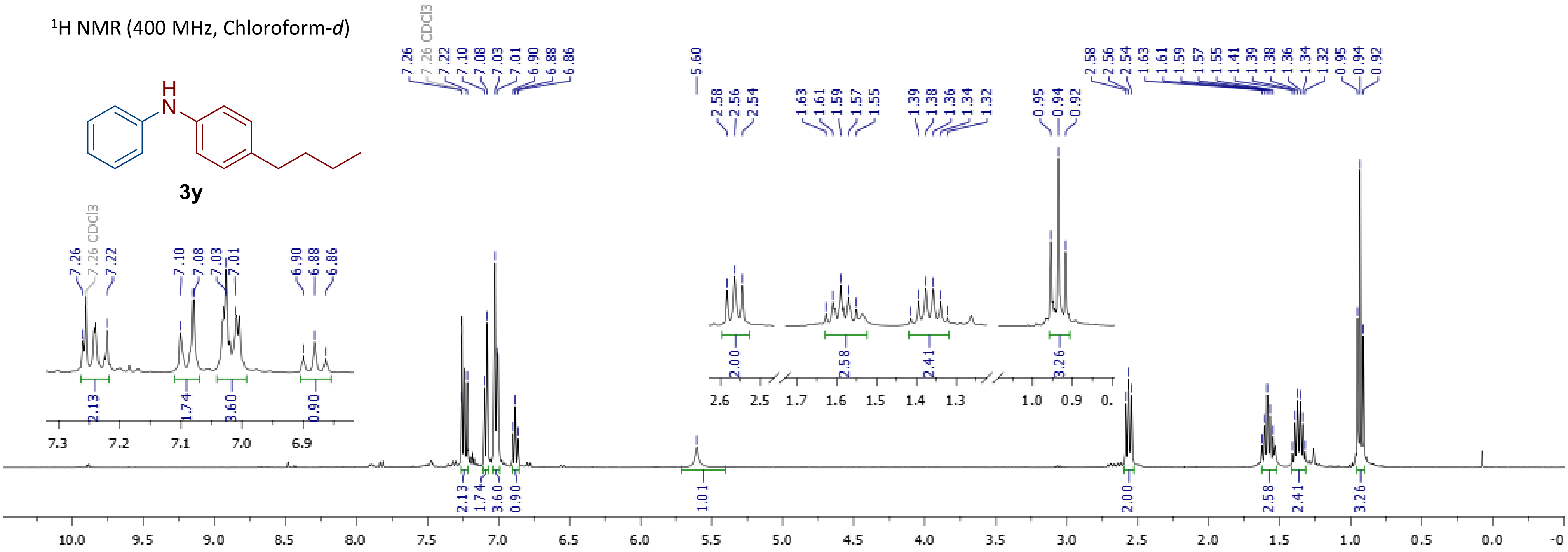

^{13}C NMR (101 MHz, Chloroform-*d*)

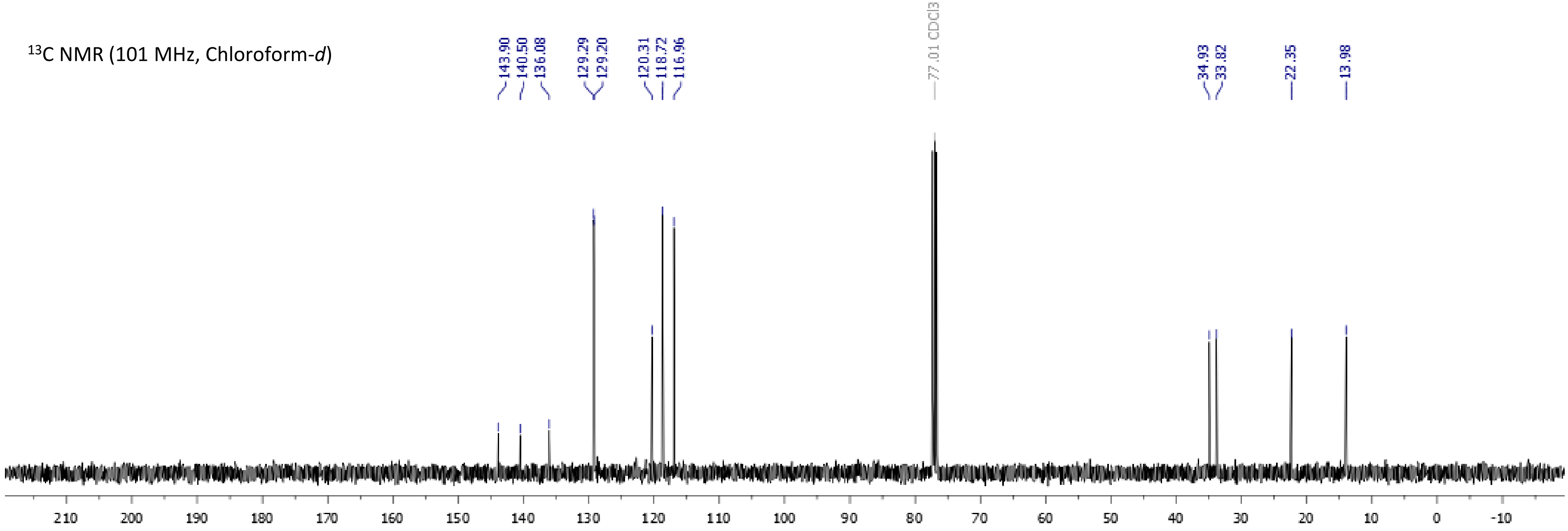

^{1}H NMR (400 MHz, Chloroform-*d*)

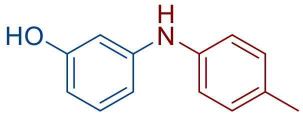

4

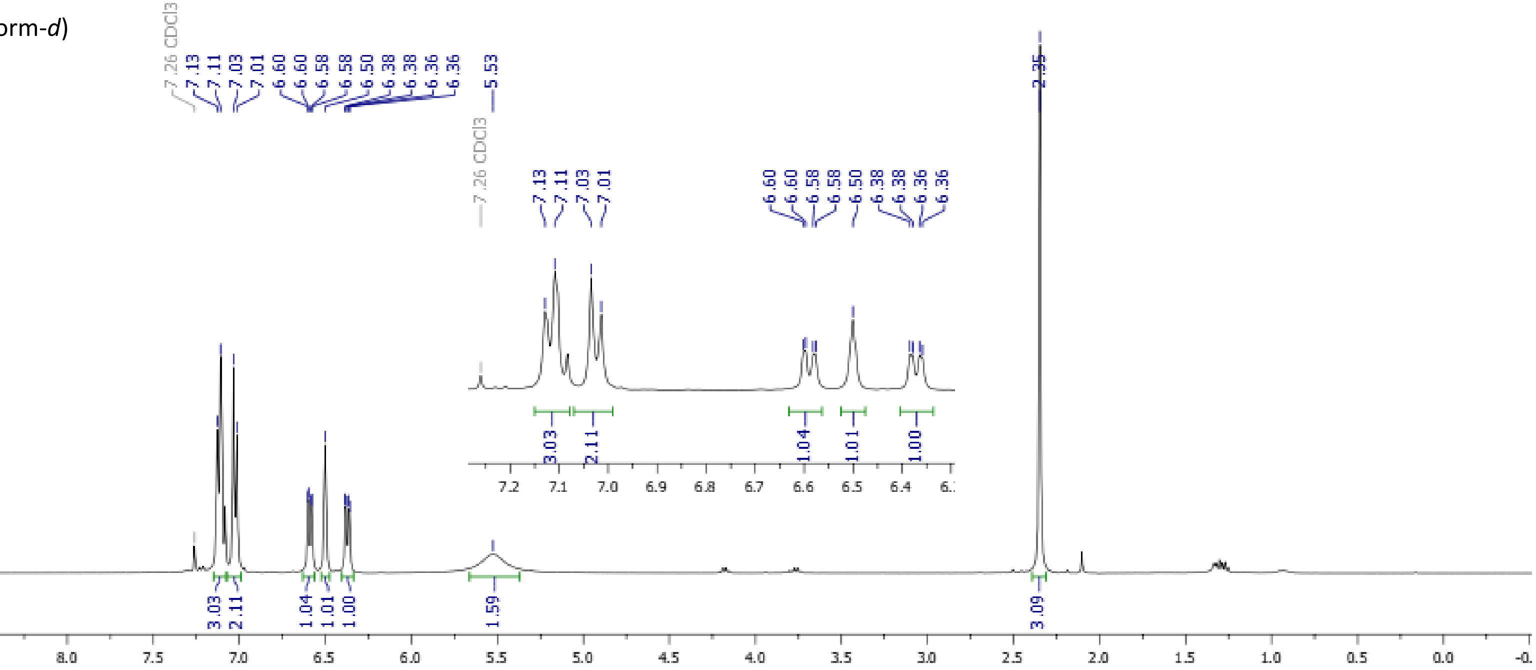

^{13}C{1H} NMR (101 MHz, Chloroform-*d*)

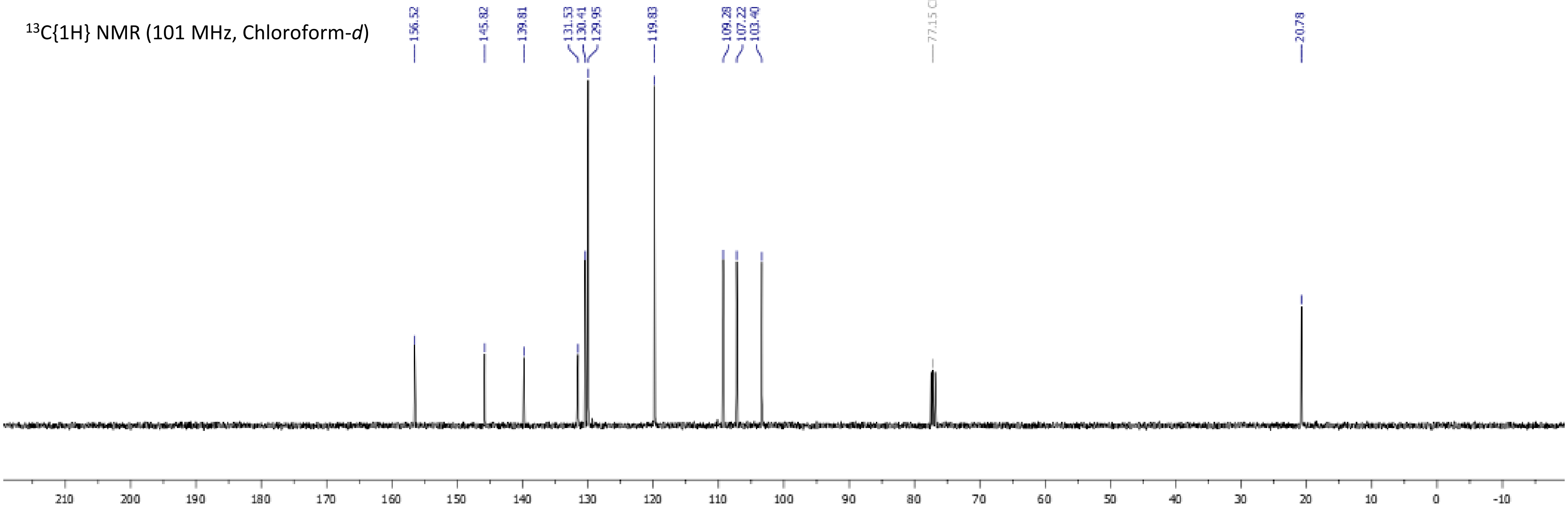

^{1}H NMR (500 MHz, DMSO-d_6)

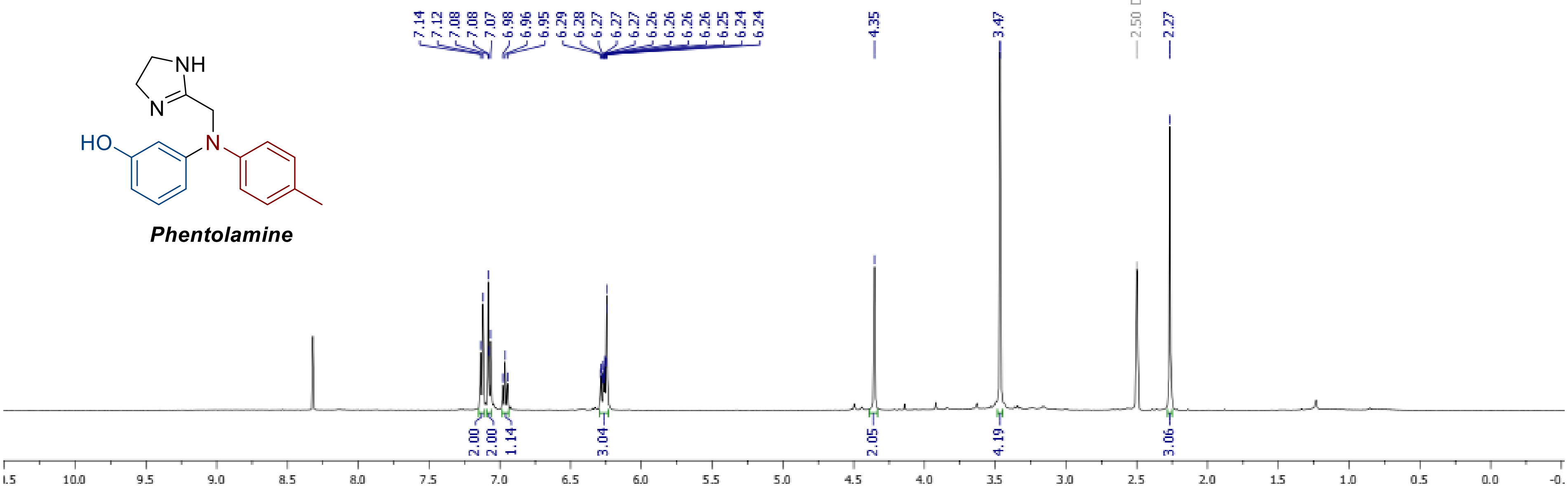

$^{13}C\{1H\}$ NMR (101 MHz, Chloroform-*d*)

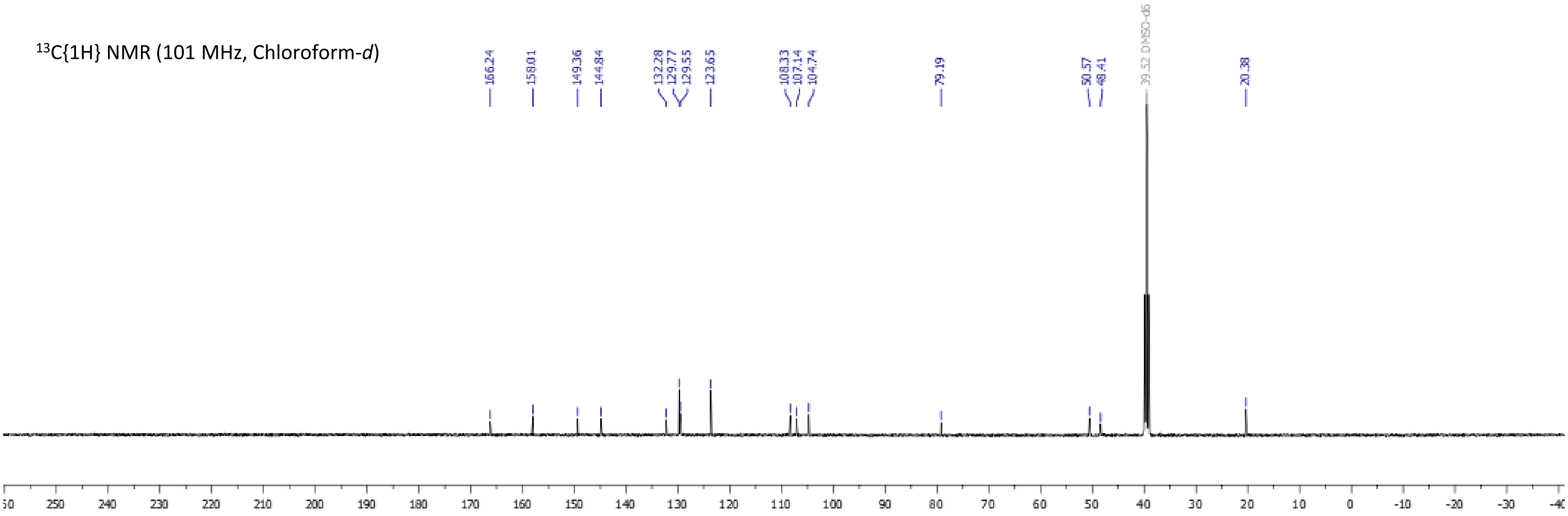

Supplement: Supplementary file 1 [file jo5c01253_si_001.pdf]
